# High‐Performance Thermoelectric SnSe: Aqueous Synthesis, Innovations, and Challenges

**DOI:** 10.1002/advs.201902923

**Published:** 2020-02-13

**Authors:** Xiao‐Lei Shi, Xinyong Tao, Jin Zou, Zhi‐Gang Chen

**Affiliations:** ^1^ Centre for Future Materials University of Southern Queensland Springfield Central Brisbane Queensland 4300 Australia; ^2^ College of Materials Science and Engineering Zhejiang University of Technology Hangzhou 310014 China; ^3^ School of Mechanical and Mining Engineering The University of Queensland Brisbane Queensland 4072 Australia; ^4^ Centre for Microscopy and Microanalysis The University of Queensland Brisbane Queensland 4072 Australia

**Keywords:** aqueous solution, characterization, performance, SnSe, thermoelectrics

## Abstract

Tin selenide (SnSe) is one of the most promising candidates to realize environmentally friendly, cost‐effective, and high‐performance thermoelectrics, derived from its outstanding electrical transport properties by appropriate bandgaps and intrinsic low lattice thermal conductivity from its anharmonic layered structure. Advanced aqueous synthesis possesses various unique advantages including convenient morphology control, exceptional high doping solubility, and distinctive vacancy engineering. Considering that there is an urgent demand for a comprehensive survey on the aqueous synthesis technique applied to thermoelectric SnSe, herein, a thorough overview of aqueous synthesis, characterization, and thermoelectric performance in SnSe is provided. New insights into the aqueous synthesis‐based strategies for improving the performance are provided, including vacancy synergy, crystallization design, solubility breakthrough, and local lattice imperfection engineering, and an attempt to build the inherent links between the aqueous synthesis‐induced structural characteristics and the excellent thermoelectric performance is presented. Furthermore, the significant advantages and potentials of an aqueous synthesis route for fabricating SnSe‐based 2D thermoelectric generators, including nanorods, nanobelts, and nanosheets, are also discussed. Finally, the controversy, strategy, and outlook toward future enhancement of SnSe‐based thermoelectric materials are also provided. This Review guides the design of thermoelectric SnSe with high performance and provides new perspectives as a reference for other thermoelectric systems.

## Graphical abstract

Herein, aqueous‐solution‐based synthesis, characterizations, and thermoelectric performance in SnSe thermoelectrics are thoroughly reviewed by providing new insights including vacancy synergy, crystallization design, doping limit breakthrough, local lattice imperfection engineering, and a promising flexible thermoelectric generator based on appropriate synthesis design.

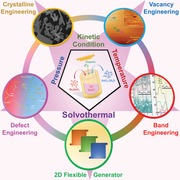

## 1 Introduction

With increasing the interest in global energy dilemma, space exploration, medical physics advances, and resource exploration, to develop a power system that can supply itself from waste heat is highly needed, and thermoelectric power generation is particularly suitable for these applications.^1^ Thermoelectric materials can realize the mutual conversion between thermal and electric energies through thermoelectric effects. Harvesting electricity from temperature difference is a potential energy utilization method to meet the challenge of the traditional fuel resource depletion in recent years, making thermoelectrics be a research topic with significant attentions for broad application prospects.^2^ To evaluate the thermoelectric efficiency, a dimensionless figure‐of‐merit ZT is described as^3^

(1) $$\text{ZT}\;=\;\;\frac{S^{2}\sigma }{\kappa }T$$

where *S*, σ, κ, and *T* are the Seebeck coefficient, the electrical conductivity, the thermal conductivity, and the absolute temperature, respectively.^3^ *S* ^2^σ is the power factor to evaluate the thermopower.^4^ κ is described as^5^

(2) $$\kappa \;=\;D\;\rho C_{\text{p}}=\;\;\kappa_{\text{e}}+\;\;\kappa_{\text{l}}$$

where *D*, ρ, *C*_p_, κ_e_, and κ_l_ are the thermal diffusivity, mass density, specific heat, and electronic and lattice thermal conductivities, respectively.^5^ In order to achieve high ZT, both high *S* ^2^σ and low κ are needed.^6^ To meet this goal, computational studies indicate that appropriately tuning the band structure can effectively improve *S* ^2^σ,^7^ and further strengthening the phonon scattering can reduce κ_l_,^8^ contributing a significant ZT improvement.

**Figure 1:**
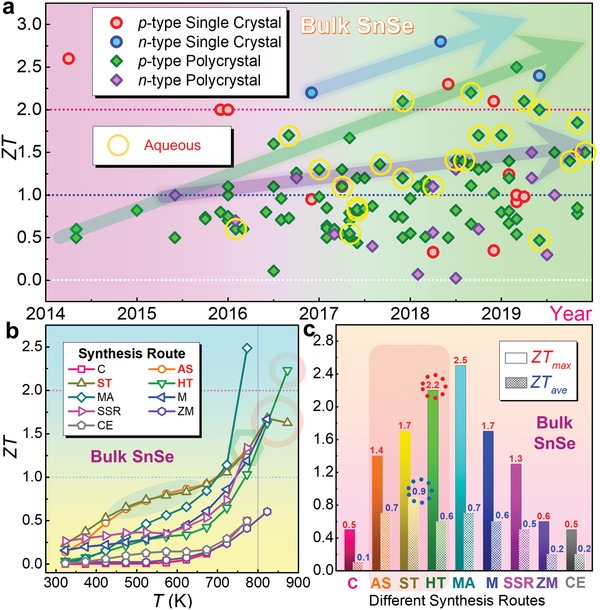
A summary of ZTs for SnSe‐based thermoelectric materials. a) The timeline for state‐of‐the‐art SnSe bulks thermoelectric materials,^11^, ^12^, ^13^, ^14^, ^15^, ^16^, ^17^, ^18^, ^19^, ^20^, ^21^, ^22^, ^23^, ^24^, ^25^, ^26^, ^27^, ^28^, ^29^, ^30^, ^31^, ^32^, ^33^, ^34^, ^35^, ^36^, ^37^, ^38^, ^39^, ^40^, ^41^, ^42^, ^43^, ^44^, ^45^, ^46^, ^47^, ^48^, ^49^, ^50^, ^51^, ^52^, ^53^, ^54^, ^55^, ^56^, ^57^, ^58^, ^59^, ^60^, ^61^, ^62^, ^63^, ^64^, ^65^, ^66^, ^67^, ^68^, ^69^, ^70^, ^71^, ^72^, ^73^, ^74^, ^75^, ^76^, ^77^, ^78^, ^79^, ^80^, ^81^, ^82^, ^83^, ^84^, ^85^, ^86^, ^87^, ^88^, ^89^, ^90^, ^91^, ^92^, ^93^, ^94^, ^95^, ^96^, ^97^, ^98^, ^99^, ^100^, ^101^, ^102^, ^103^, ^104^, ^105^, ^106^, ^107^, ^108^, ^109^, ^110^, ^111^, ^112^, ^113^, ^114^, ^115^, ^116^, ^117^, ^118^, ^119^, ^120^, ^121^, ^122^, ^123^, ^124^, ^169^, ^170^, ^171^, ^172^, ^173^, ^174^, ^175^, ^176^, ^177^, ^178^, ^179^, ^180^, ^181^, ^182^ the performance achieved by solution route are circled by yellow. b) Temperature‐dependent ZT and c) corresponding peak and average ZT values for polycrystalline SnSe through different fabrication techniques.^13^, ^16^, ^22^, ^46^, ^58^, ^62^, ^95^, ^99^, ^101^ Here, melting, arc‐melting, mechanical alloying, solid‐state reaction, combustion, aqueous solution, hydrothermal, solvothermal, and chemical exfoliation are abbreviated as M, AM, MA, SSR, C, AS, HT, ST, and CE, respectively.

Among the state‐of‐the‐art thermoelectric materials, tin selenide (SnSe) is one of the most promising candidates to apply to thermoelectric devices due to its environmentally friendly feature, high cost‐effectiveness, and outstanding thermoelectric performance derived from its appropriate bandgap of ≈0.9 eV and intrinsic low κ_l_.^9^, ^10^ **Figure** **1**a shows the development timeline for all SnSe‐based bulk thermoelectric materials,^11^, ^12^, ^13^, ^14^, ^15^, ^16^, ^17^, ^18^, ^19^, ^20^, ^21^, ^22^, ^23^, ^24^, ^25^, ^26^, ^27^, ^28^, ^29^, ^30^, ^31^, ^32^, ^33^, ^34^, ^35^, ^36^, ^37^, ^38^, ^39^, ^40^, ^41^, ^42^, ^43^, ^44^, ^45^, ^46^, ^47^, ^48^, ^49^, ^50^, ^51^, ^52^, ^53^, ^54^, ^55^, ^56^, ^57^, ^58^, ^59^, ^60^, ^61^, ^62^, ^63^, ^64^, ^65^, ^66^, ^67^, ^68^, ^69^, ^70^, ^71^, ^72^, ^73^, ^74^, ^75^, ^76^, ^77^, ^78^, ^79^, ^80^, ^81^, ^82^, ^83^, ^84^, ^85^, ^86^, ^87^, ^88^, ^89^, ^90^, ^91^, ^92^, ^93^, ^94^, ^95^, ^96^, ^97^, ^98^, ^99^, ^100^, ^101^, ^102^, ^103^, ^104^, ^105^, ^106^, ^107^, ^108^, ^109^, ^110^, ^111^, ^112^, ^113^, ^114^, ^115^, ^116^, ^117^, ^118^, ^119^, ^120^, ^121^, ^122^, ^123^, ^124^ from which a record high ZT of ≈2.8 at 773 K was found in the n‐type SnSe single crystal,^11^ derived from its ultralow κ_l_ of ≈0.18 W m^−1^ K^−1^ and high *S* ^2^σ of ≈9.0 µW cm^−1^ K^−2^ at this temperature.^125^ Such a high ZT is also very competitive to other state‐of‐the‐art thermoelectric systems which possess ZTs > 2, such as PbTe,^126^, ^127^, ^128^, ^129^, ^130^, ^131^, ^132^, ^133^, ^134^ GeTe,^135^, ^136^, ^137^, ^138^, ^139^, ^140^, ^141^, ^142^, ^143^, ^144^, ^145^, ^146^, ^147^ Cu_2_Se/Cu_2_S,^148^, ^149^, ^150^, ^151^, ^152^, ^153^, ^154^, ^155^, ^156^, ^157^ and AgSbTe_2_.^158^ Inspired by the full potentials for further improving ZT via reducing κ_l_ and tuning carrier concentration n (for electrons) and/or p (for holes), SnSe‐based thermoelectric materials have attracted much attentions in recent years.^159^, ^160^

SnSe single crystals have been reported to have outstanding thermoelectric properties.^5^ However, due to their poor mechanical properties,^3^, ^9^ rigid crystal growth conditions,^3^, ^9^, ^161^, ^162^, ^163^, ^164^, ^165^ and high cost for production, SnSe single crystals are limited for their industrial scale‐up.^3^, ^9^, ^78^ To solve this challenge, polycrystalline SnSe as an alternative has become a research topic,^3^ and significant progress has been made for enhancing their ZT values, as shown in Figure 1a.^3^ Considering that the performance of polycrystalline SnSe is still lower than its single crystal counterparts due to its relatively high κ and low σ,^166^ simultaneously achieving appropriate *n*/*p* and low κ_l_ is urgently needed.

SnSe is a layered orthorhombic‐structured materials with strong anisotropy.^3^, ^12^ Historically, an ideal SnSe‐based thermoelectric material should be polycrystalline SnSe composed by grains with single‐crystal like anisotropy and optimized *n*/*p*.^3^ However, traditional fabrication techniques such as melting and mechanical alloying are difficult to realize morphology control of the fabricated SnSe products,^3^ thus there are limitations for these synthesis routes to achieve high thermoelectric performance in polycrystalline SnSe. To solve this issue, as one of the most convenient methods to fabricate SnSe crystals, advanced aqueous synthesis‐based solution routes have been constructively employed to achieve high thermoelectric performance in polycrystalline SnSe.^3^ Compared with traditional melting and mechanical alloying routes,^167^, ^168^ advanced aqueous synthesis‐based solution routes possess unique advantages, including convenient morphology control to achieve high anisotropy,^25^ exceptional high doping solubility to tune the *n*/*p*,^48^, ^71^ intensive local lattice imperfections to reduce κ_l_,^48^, ^71^ special doping behaviors,^22^, ^71^ and distinctive vacancy engineering for property synergy.^22^, ^24^, ^25^ Figure 1b,c shows temperature‐dependent ZT and corresponding peak and average ZT values for polycrystalline SnSe fabricated through different techniques,^13^, ^16^, ^22^, ^46^, ^58^, ^62^, ^95^, ^99^, ^101^ for each technique we have chosen the reported highest performance. As can be clearly seen, aqueous solution routes (including solvothermal, hydrothermal and traditional aqueous route) can achieve both high peak and average ZTs, indicating considerable potentials of the solution routes possess for achieving high thermoelectric performance in polycrystalline SnSe.

Although the aqueous solution route has distinctive features, there is still lack of a comprehensive Review to summarize these unique features on enhancing the thermoelectric performance of polycrystalline SnSe. Based on this urgent demand, in this article, we provide a thorough overview toward an integrated understanding of synthesis, characterizations, and performance in polycrystalline SnSe. We provide new insights into the strategies of aqueous synthesis route, including vacancy synergy, crystallization design, solubility breakthrough, and local lattice imperfection engineering, to build the inherent links between the aqueous synthesis‐induced nanostructural characteristics and the excellent thermoelectric performance. Besides, the advances of solution route on fabricating SnSe nanocrystal‐based 2D thermoelectric generators are discussed in detail, and the controversy, strategy, and outlook toward future enhancements of SnSe‐based thermoelectric materials are also provided. This Review will provide guidance in the design of SnSe‐based thermoelectric materials with high performance and robust stability, and provide new perspectives as reference for other thermoelectric system.

## 2 Fundamental

Aqueous solution synthesis refers to the synthesis through chemical reactions in aqueous solution above the boiling point of the solvent,^183^ which is particularly suitable for fabricating SnSe crystals.^3^ In this section, the fundamental mechanisms of solution‐based synthesis on SnSe are discussed, followed by recent advances in SnSe, including thermodynamics, crystal characteristics, band structures, evaluation of thermoelectric performance through modelling, anharmonic bonding, and potential phonon scattering occurred in SnSe. The kinetic conditions for synthesizing SnSe crystals are also comprehensively summarized.

### 2.1 Solution‐Based Synthesis

#### 2.1.1 Hydrothermal

Hydrothermal synthesis is an important branch of inorganic synthesis, which has a history of more than 100 years.^183^ Hydrothermal refers to a method of preparing materials in a sealed pressure vessel using water as a solvent, in which precursors can be dissolved, reacted, and crystallized, similar to traditional aqueous solution route. The difference is that hydrothermal reaction occurs under high‐temperature and high‐pressure conditions, which are usually above the boiling temperature of water to achieve a high vapor pressure and in turn to meet specific critical conditions. Therefore, the synthesized products are subject to post‐treatment, including separation, washing, and drying. Compared with other material fabrication techniques, the products prepared by the hydrothermal method have complete crystal growth, controllable crystal size, uniform distribution, weak agglomeration, considerable efficiency, competitive productivity, and more cost‐effectiveness derived from using relatively cheap raw materials, and are easy to achieve suitable stoichiometry and desired crystal form at a much lower temperature. For SnSe, hydrothermal has been demonstrated as a convenient route to achieve SnSe micro/nanocrystals with controllable size and yielding rate,^93^, ^184^, ^185^, ^186^ thus has been considered as a promising approach to fabricate polycrystalline SnSe for subsequently sintering them into bulks for property measurements or device assembly. In particular, the preparation of SnSe crystals by the hydrothermal method avoids inducing impurity during synthesis, contributing to a high sintering activity. In addition, driven by the layered crystal structure, hydrothermal‐grown SnSe are more likely to form 2D structures, which have been widely observed in other layered materials grown by the hydrothermal method.^60^

**Figure 2:**
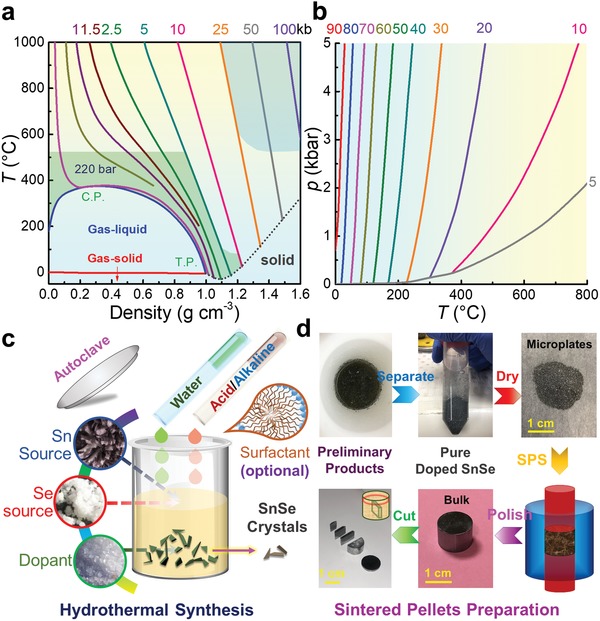
Illustrations of hydrothermal synthesis for fabrication SnSe crystals and sintered polycrystalline bulks. a) Plots of temperature–density of water with pressure as a parameter.^187^ b) Plots of dielectric constant of water versus temperature and pressure. Reproduced with permission.^188^ Copyright 1981, Elsevier. c) Illustration of typical hydrothermal synthesis, and d) obtained SnSe products and subsequent sintering and cutting processes for thermoelectric performance evaluation. c,d) Reproduced with permission.^22^ Copyright 2019, Wiley.

In a typical hydrothermal synthesis, a water solution is used as a reaction medium in a special closed reaction vessel (autoclave) to create a high‐temperature (>100 °C), high‐pressure (1–100 MPa) reaction environment by heating the reaction vessel, making the normally insoluble precursors be dissolved, reacted, and crystallized. **Figure** **2**a plots the relationship of temperature–density of water under different pressure,^187^ from which it is clearly seen that, with increasing the pressure and temperature, the ion products of water rapidly increase. When under significantly high pressure and high temperature (15–20 GPa and 1000 °C), the density of water is in the range of 1.7–1.9 g cm^−3^. In this condition, water can be completely dissociated into OH^−^ and H_3_O^+^, behaved as a molten salt. Besides, the water viscosity decreases with rising the temperature. At 500 °C and under 0.1 GPa, the water viscosity is only ≈10% of its value under normal condition. In this situation, the mobilities of ions and molecules are much higher than under normal condition, so that many chemical reactions can take place in this environment. Besides, for the chemical reactions happened during hydrothermal synthesis, water is also used as a solvent in some occasions, in which the dielectric constant of water is one of the key factors, as shown in Figure 2b. With increasing the temperature and pressure, the dielectric constant decreases,^188^ indicating that temperature and pressure play dominant roles in the dielectric constant of water. Based on these fundamentals, hydrothermal syntheses have many advantages as hydrothermal syntheses can produce crystalline phases that are not stable at the melting point, and fabricate materials which have a high vapor pressure near their melting points. Meanwhile, hydrothermal synthesis can maintain the designed composition of synthesized materials, exhibiting high operability and adjustability. Besides, hydrothermal synthesis is environmentally safe and cheap, thus is a particularly suitable route to fabricate SnSe with high productivity and high efficiency.

Figure 2c illustrates a typical hydrothermal synthesis of SnSe crystals, in which the solvent is water, the Sn sources are SnCl_2_ and SnCl_2_·2H_2_O,^91^, ^121^ and the Se sources are Se and SeO_2_.^88^, ^189^ If doping processes are needed, dopant sources are needed to further modify the composition of the synthesized products and in turn their performances.^49^ Therefore, high purities (>99%) of all precursors are needed to avoid potential impurities and/or doping errors. Taking SnCl_2_·2H_2_O and Se for examples, in a typical synthesis, SnCl_2_·2H_2_O is dissolved in water and kept stirring for a certain time at room temperature to ensure a complete dissolve of following reaction

(3) $${\text{SnCl}}_{2}\cdot 2{\text{H}}_{2}\text{O}\;\;\to \;\;{\text{Sn}}^{2+}+\;\;2{\text{Cl}}^{-}+\;\;2{\text{H}}_{\text{2}}\text{O}$$

To dissolve Se, alkaline such as NaOH or KOH is used in the solution to ensure the reaction of

(4) $$3\text{Se}\;\;+\;\;6{\text{OH}}^{-}\to \;\;2{\text{Se}}^{2-}+\;\;{\text{SeO}}_{3}^{2-}+\;\;{\text{3H}}_{\text{2}}\text{O}$$

The solution is then sealed in a polytetrafluoroethylene‐lined stainless‐steel autoclave. The autoclave is heated above 100 °C (boiling temperature of water to provide sufficient vapor pressure for ensuring chemical reactions) in an oven, maintained for a certain time, followed by naturally cooled to room temperature. Sometimes surfactant such as polyvinylpyrrolidone (PVP) is used to contribute to a better crystallization, and catalyst can also be used to accelerate either precursors dissolving or chemical reactions.^190^ During this stage, a reaction can take place

(5) $${\text{Sn}}^{2+}+\;\;{\text{Se}}^{2-}\to \;\;\text{SnSe}$$

To achieve SnSe crystals with high purity, post‐treatments, including separation, washing, and drying, are needed, as illustrated in Figure 2d. Traditionally, the synthesized SnSe products are collected by centrifugation and washed by deionized water for several times before drying in the oven at temperature below 70 °C for a certain time (usually 12 h). At this stage, it is important to avoid oxidization on the surface of synthesized products because oxidization can significantly increase the thermal conductivity of SnSe,^3^ resulting in a low ZT. A vacuum oven is generally recommended with controllable synthesis temperature and time. Finally, the dried products can be conveniently sintered via either traditional hot‐pressing or spark plasma sintering (SPS) to achieve polycrystalline SnSe bulks for thermoelectric performance evaluation and/or their device assembling, as shown in Figure 2d.

#### 2.1.2 Solvothermal

Compared with hydrothermal, solvothermal‐based solution methods use nonaqueous rather than water, and the syntheses are always carried out at a relative high temperature,^183^, ^191^ thus hydrothermal can be treated as a class of solvothermal method. Since different solvents possess different characteristics, such as boiling point, vapor pressure, and solubility of precursors, there exist full potentials for applying different solvents for synthesizing SnSe crystals with distinctive features, including tuning vacancy concentration to achieve higher electrical transport performance,^22^, ^25^ crystallization design to fabricate large crystals for high anisotropy,^25^ solubility breakthrough for increase doping potentials,^22^, ^48^, ^71^ and local lattice imperfection inducing to strengthen the phonon scattering,^22^, ^48^, ^71^ making solvothermal synthesis a “wondrous magic” with considerable variation and full potentials for SnSe‐based thermoelectric material design. **Table** **1** lists the key physical parameters of the solvents used in solvothermal synthesis (including hydrothermal when the solvent is water),^183^, ^191^ including molecular weight Mr, ρ, melting point *T*_m_, boiling point *T*_b_, dielectric constant ε, dipole moment *µ*_d_, and solvent polarity *E* ^T^_N_, respectively. Among these parameters, *E* ^T^_N_, defined as the sum of the interaction of solvent and solute including Coulomb force, induction force, dispersion force, H‐bond, and charge transport force, is the key factor to describe the solvation property of solvent.

**Table 1:** Physical parameters of the solvents used in solvothermal synthesis.^183^, ^191^ Here Mr, ρ, *T*_m_, *T*_b_, ε, *µ*_d_, and *E* ^T^_N_ are molecular weight, mass density (g cm^−3^), melting point (°C), boiling point (°C), dielectric constant (C^2^ N^−1^ M^−2^), dipole moment (D), and solvent polarity, respectively

Solvent	Mr	*D*	*T*_m_	*T*_b_	ε	*µ*_d_	*E* ^T^_N_
Water	18.01	1	0	100	80.4	1.94	1
Methyl alcohol	32.04	0.791	−98	65	32.6	1.7	0.762
Ethyl alcohol	46.07	0.785	−130	78	24.3	1.69	0.654
Propanol	60.1	0.804	−127	97	20.1	1.66	0.602
2‐Propanol	60.1	0.785	−90	82	18.3	1.66	0.546
Butanol	74.12	0.81	−90	118	17.1	1.66	0.602
2‐Butanol	74.12	0.807	−115	98	15.8		0.506
2‐Methyl‐1‐propanol	74.12	0.802	−10	108	17.7	1.64	0.552
2‐Methyl‐2‐propanol	74.12	0.786	25	83			0.389
Pentanol	88.15	0.811	−78	137	13.9	1.8	0.568
2‐Pentanol	88.15	0.809		120	13.8	1.66	–
3‐Methyl‐1‐butanol	88.15	0.809	−11	130	14.7	1.82	0.565
2‐Methyl‐2‐butanol	88.15	0.805	−12	102	7	1.7	0.321
Hexyl alcohol	102.18	0.814	−52	157	13.3		0.559
1‐Heptanol	116.2	0.822	−36	176	12.1		0.549
2‐Methyl‐2‐hexanol	116.2	0.8119		139.4	–	–	–
Tetradecanol	214.39	0.823	39	289		–	–
Cyclohexanol	100.16	0.963	21	160	15	1.9	0.5
Benzyl alcohol	108.14	1.045	−15	205	13.1	1.7	0.608
Ethylene glycol	62.07	1.109	−11	199	37.7	2.28	0.79
1,3‐Propanediol	76.1	1.053	−27	214	35	2.5	0.747
1,2‐Propanediol	76.1	1.036	−60	187	32	2.25	0.722
1,4‐Butanediol	90.12	1.017	16	230	31.1	2.4	0.704
1,3‐Butanediol	90.12	1.004	−50	207			0.682
Diethylene glycol	106.12	1.118	−10	245			0.713
Triethylene glycol	150.18	1.123	−7	287	23.7	5.58	0.704
Tetraethylene glycol	194.23	1.125	−6	314			0.664
Glycerol	92.09	1.261	20	180	42.5		0.812
Diglycerol	166.18	1.3					

For solvothermal‐based synthesis of SnSe crystals, the process is similar to hydrothermal, as shown in Figure 2c. Taking ethylene glycol (EG, C_2_H_6_O_2_) as solvent for an example, Sn sources are SnCl_2_ and SnCl_2_·2H_2_O,^26^, ^192^ similar to hydrothermal,^91^, ^121^ and Se sources are Se,^62^ SeO_2_,^26^ and Na_2_SeO_3_.^71^ Sometimes dopant sources are needed to further modify the performance of synthesized products.^48^ Taking SnCl_2_ and Na_2_SeO_3_ for examples, in a typical solvothermal synthesis, SnCl_2_ is dissolved in EG and kept stirring for a certain time at room temperature to ensure a complete dissolve through a reaction as

(6) $${\text{SnCl}}_{2}\overset{C_{2}H_{6}O_{2}}{\to }{\text{Sn}}^{2+}+\;\;2{\text{Cl}}^{-}$$

To produce Se^2−^, EG acts as the precursor,^25^ and alkaline such as NaOH or KOH is used in the solution, acting as both pH adjuster and precursor to benefit the chemical reactions as^25^

(7) $${\text{Na}}_{2}{\text{SeO}}_{3}\overset{C_{2}H_{6}O_{2}}{\to }\;\;2{\text{Na}}^{+}+\;\;{\text{SeO}}_{3}^{2-}$$

(8) $${\text{SeO}}_{3}^{2-}+\;\;{\text{C}}_{\text{2}}{\text{H}}_{\text{6}}{\text{O}}_{\text{2}}\to \;\;\text{Se}+\;\;{\text{C}}_{\text{2}}{\text{H}}_{\text{2}}{\text{O}}_{\text{2}}+\;\;{\text{H}}_{\text{2}}\text{O}+\;\;{\text{2OH}}^{-}$$

(9) $$\text{3Se}+{\text{6OH}}^{-}\to \;\;2{\text{Se}}^{2-}+\;\;{\text{SeO}}_{3}^{2-}+\;\;{\text{3H}}_{\text{2}}\text{O}$$

The solution is then sealed in a polytetrafluoroethylene‐lined stainless‐steel autoclave. The autoclave is heated above 200 °C (boiling temperature of EG to provide sufficient vapor pressure for ensuring chemical reactions) in an oven, maintained for a certain time, followed by naturally cooled to room temperature. Sometimes surfactants (such as PVP) are used to ensure the better crystallization,^62^ and catalyst can also be used to accelerate either precursors dissolving or chemical reactions.^89^ During this stage, reactions (2) and (3) take place.

After the synthesis, the post‐treatments may be needed, similar to hydrothermal. The only difference is that the synthesized SnSe products should be collected by centrifugation and washed by both deionized water to remove all salts and ions remained in the solution and ethanol to remove the un‐reacted solvent and organic byproducts.

#### 2.1.3 Microwave Assistance

Occasionally, to increase the density of grain boundaries in sintered polycrystalline SnSe for strengthening the mechanical properties (such as hardness and compressing strength) and/or reduce κ_l_, fabricating smaller SnSe crystals are needed, where traditional solvothermal route is difficult to achieve such a goal. To meet this requirement, microwave‐assisted solvothermal synthesis can be a key resolution. Different from the traditional solvothermal route needing conventional oven heating, microwave‐assisted solvothermal receives heat from microwave radiation, thus the heat is much more homogeneous with a high heating efficiency. Besides, considering that many chemical reactions show significant differences under microwave heating conditions in aspects of the reaction rate and product selectivity, it is important to understand the principle of microwave radiation.

**Figure 3:**
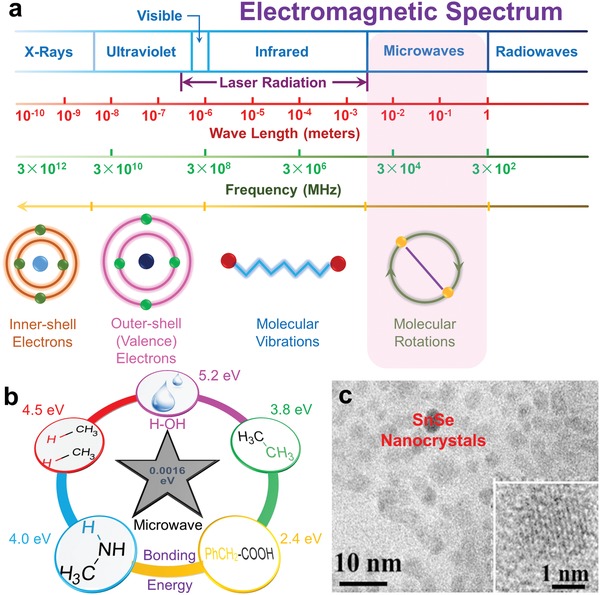
Illustrations of microwave‐assisted solvothermal synthesis. a) A typical spectrum of common electromagnetic radiations.^183^, ^191^ b) Energy of microwave and common bonding in solvents. c) TEM image with inset HRTEM image of SnSe nanocrystals synthesized via microwave‐assisted solvothermal synthesis. Reproduced with permission.^193^ Copyright 2016, American Chemical Society.

**Figure** **3**a shows a typical spectrum of common electromagnetic radiations.^183^, ^191^ The basic principle of microwaves can be described as

(10) $$\lambda_{0}=\;\;\frac{c}{f}$$

where λ_0_ is the microwave wavelength, *c* is the microwave velocity, and *f* is the microwave frequency, which is defined as the numbers of oscillations of the electric or magnetic field in 1 s. From Figure 3a, it is clear that microwave lies in the electromagnetic spectrum between infrared waves and radio waves with the wavelength between 0.01 and 1 m, and the frequency ranges between 0.3 and 30 GHz.

Generally, microwave is a typical low energy radiation with quantum energy of only ≈1.6 × 10^−3^ eV at 2450 MHz, which is difficult to break any chemical bond (such as hydrogen bond with a bonding energy of 5.2 eV),^183^, ^191^ as illustrated in Figure 3b. However, microwave energy can be absorbed by dielectric solvent (such as water) and in turn heat is generated, which is critical for solvothermal syntheses. There are two dominant microwave energy absorption mechanisms, namely dipole rotation and ionic conduction, respectively. For dipole rotation, it refers to dielectric heating. When applying an electromagnetic field, molecular rotation occurs in solvent containing polar molecules with an electrical dipole moment, which will completely or partially align themselves. When this electromagnetic field alternates, the directions of molecules change, resulting in rotation of molecules and colliding between adjacent molecules. However, the rotation cannot fully follow the field changes since it depends upon the size and the dielectric coefficient of the molecules, thus the field energy is converted to kinetic and thermal energy. Simply, such heating can be described as the molecular friction. For ionic conduction, it refers to ion movements with the electronic field. When this field changes, ions with the same charge migrate away from the field, which will affect the chemical reaction in different ways as compared with conventional heating.

Based on these unique features, microwave‐assisted solvothermal synthesis is particularly suitable for produce SnSe nanoscale crystals with both high efficiency and low crystal dimension, thus is promising for the fabrication of 2D flexible thermoelectric generator. Figure 3c shows a typical example, in which a TEM image with inset HRTEM image shows SnSe nanocrystals with averaged size of ≈5 nm, synthesized by microwave‐assisted solvothermal synthesis.

### 2.2 SnSe

#### 2.2.1 Thermodynamics

**Figure 4:**
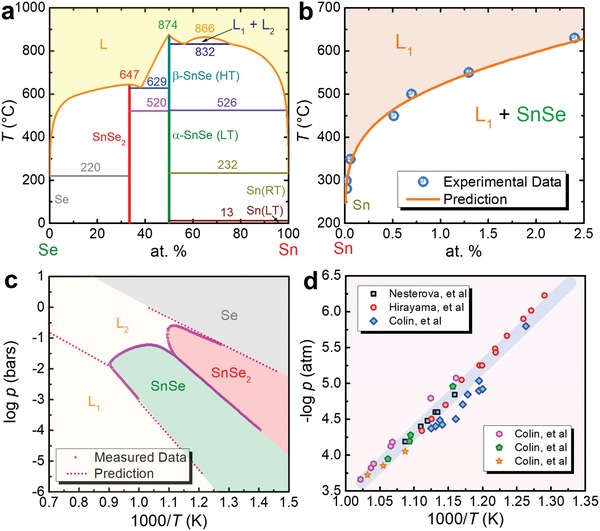
Thermodynamics of SnSe. a) The phase diagram of Sn–Se.^154^, ^195^, ^196^, ^197^ b) Sn‐rich region of the Sn–Se phase diagram.^196^ c) 1000/*T*‐dependent log *p* for Sn–Se system.^196^ d) Vapor pressure of SnSe.^198^, ^199^, ^200^

To successfully synthesize SnSe crystals using aqueous solution method (especially for the advanced solvothermal route), a key factor is that the kinetic conditions of synthesis, such as boiling point and vapor pressure, should strictly align to the thermodynamic conditions of the growth of SnSe crystals, such as formation energy and crystallization temperature. Therefore, understanding the fundamental thermodynamics of SnSe are needed in order to the design of synthesis parameters, including temperature, pressure, precursor amount, type of solvent, pH, and time. **Figure** **4**a shows the Sn–Se binary phase diagram, shows a high melting point of 1134 K for SnSe. Figure 4b shows a liquid miscibility gap, a monotectic reaction, and a eutectic reaction in Sn–SnSe region. The eutectic point of L1 $\Leftrightarrow^{\;}$ (Sn) + SnSe is very close to pure Sn, and this should be one of the reasons that occasionally Sn secondary phase is formed in aqueously synthesized SnSe. Besides, SnSe has two phases, namely α‐SnSe (*T* < ≈800 K) with lattice parameters of *a* = 11.37 Å, *b* = 4.19 Å, and *c* = 4.44 Å and a space group of *Pnma* and β‐SnSe (*T* > ≈800 K) with lattice parameters of *a* = 4.31 Å, *b* = 11.71 Å, and *c* = 4.32 Å and a space group of *Cmcm*.^3^, ^194^ In this Review, we focus on α‐SnSe phase because it has a much stable performance than β‐SnSe.

In traditional melting and/or solid‐state reaction routes, to synthesize SnSe solid products, the enthalpy change Δ*H* ^o^_298,f_ of SnSe is −21.5 ± 1.7 K cal mol^−1^ for the solid reaction as^198^, ^200^

(11) $$\text{Se}\left(\text{s}\right)\;\;+\;\;\text{Sn}\left(\text{s}\right)\;\;\to \;\;\text{SnSe}\left(\text{s}\right)$$

which is essential for guiding the synthesis of SnSe products. To explore the thermodynamic conditions of SnSe, Figure 4c shows 1000/*T*‐dependent log *p* for the Sn–Se system,^196^ where the unit of pressure *p* is bars. As can be clearly seen, appropriate temperature and pressure are both needed to achieve pure SnSe and avoid to produce Se and/or SnSe_2_ second phases because these two secondary phases show typical n‐type feature,^201^, ^202^, ^203^, ^204^ which harm the performance of p‐type SnSe. Besides, to avoid the evaporation of fabricated SnSe, the temperature and pressure should be controlled within a reasonable range, and the measured vapor pressure of SnSe can be referred as shown in Figure 4d,^198^, ^199^, ^200^ which shows a linear relationship between 1000/*T* and log *p*.

#### 2.2.2 Crystal Structure

**Figure 5:**
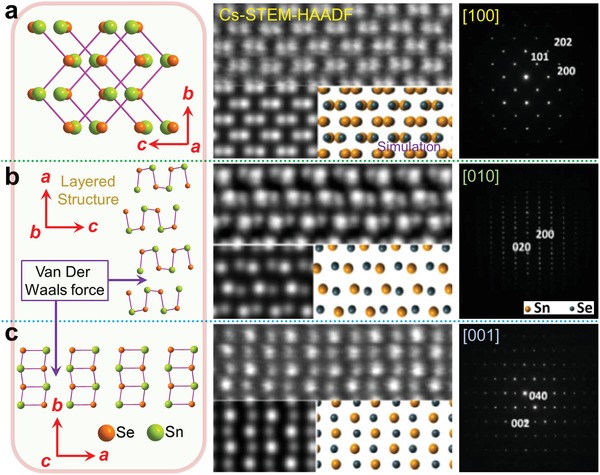
Crystal structure of SnSe (α‐SnSe). Crystal structures, Cs‐HAADF‐STEM images with multislice simulations, and SAED patterns of SnSe viewed along a) the *a*‐axis, b) the *b*‐axis, and c) the *c*‐axis. Reproduced with permission.^206^ Copyright 2017, Elsevier.

The grown SnSe crystals via advanced solution route mostly have plate or belt shapes, which can be described as micro/nanoplates or micro/nanobelts. This interesting phenomenon is mainly due to the nature of SnSe layered orthorhombic crystal structure, as shown in **Figure** **5**. The low temperature α‐SnSe has the orthogonal structure with lattice parameters of *a* = 11.37 Å, *b* = 4.19 Å, and *c* = 4.44 Å and a space group of *Pnma*.^205^ Figure 5a–c shows the projected crystal structures, high‐resolution spherical aberration corrected high‐angle annular dark‐field scanning transmission electron microscopy (Cs‐HAADF‐STEM) images with multislice simulations, and corresponding selected area electron diffraction (SAED) patterns of SnSe viewed along the *a*‐, *b*‐ and *c*‐axes,^206^ respectively. SnSe possesses a typical double‐layered structure,^10^ similar to SnS and black phosphorus.^207^, ^208^ In a unit cell of SnSe, there are eight atoms joined with strong hetero‐polar bonds, consisting of two planes of zigzag‐like chain.^209^, ^210^ The adjacent layers are mainly bound by a combination of van der Waals forces and a long‐range electrostatic attractions,^209^, ^211^ making it easy to crack along {100} atomic planes when shear force applied,^212^ and this is also the reason why the growth SnSe single crystals through the solution route are mainly plate‐ or belt‐like.

#### 2.2.3 Electronic Structure

Advanced aqueous solution route has been demonstrated to have full potentials for further improving doping limit and/or increasing doping possibility for specific dopants to enhance ZT,^48^, ^71^ which is a distinctive advantage compared with traditional melting and mechanical alloying routes. To improve the electrical transport of SnSe‐based thermoelectric materials, appropriate tuning of *n*/*p* is needed, guided by the following rules (here taking *n* for example)^6^

**Figure 6:**
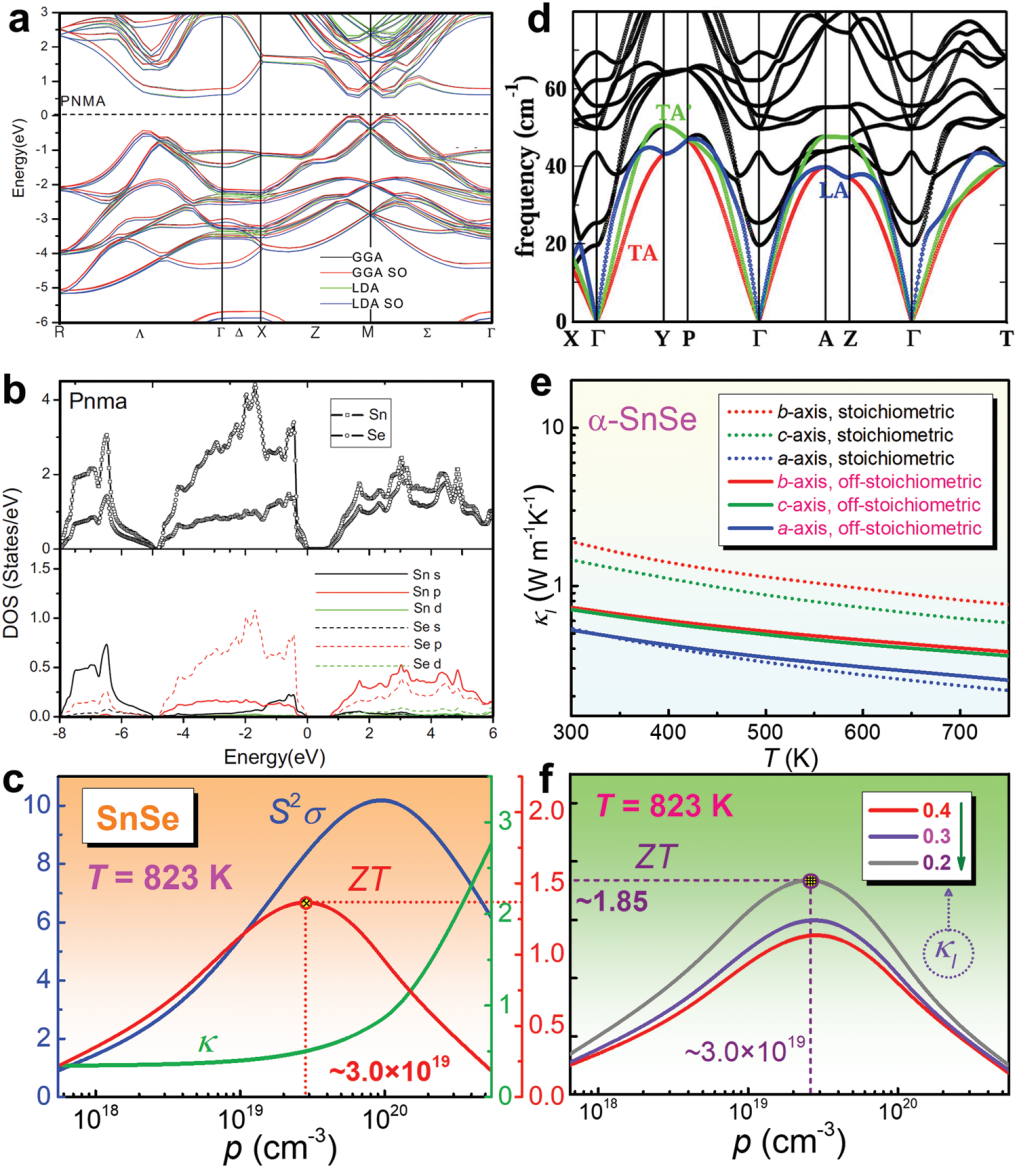
Computational advances in SnSe. a) Band structure of SnSe with GGA, GGA with spin–orbit coupling, LDA, and LDA with spin–orbit coupling for *Pnma* phase (α‐SnSe). b) total (up) and partial (down) DOS for *Pnma* phase. a,b) Reproduced with permission.^213^ Copyright 2016, Springer. c) *p*‐dependent *S^2^σ*, κ, and ZT of SnSe at *T* = 823 K with a fixed κ_l_ of 0.4 W m^−1^ K^−1^ through SPB model. Reproduced with permission.^22^ Copyright 2019, Wiley. d) Phonon dispersions of α‐SnSe, the red, green, and blue lines highlight TA, TA′, and LA modes, respectively. Reproduced with permission.^214^ Copyright 2016, Royal Society of Chemistry. e) Calculated κ_l_ of SnSe along different axes via first‐principles (stoichiometric) and Debye–Callaway model (off‐stoichiometric),^206^, ^215^ respectively. f) *p*‐dependent ZT via reducing κ_l_ by defect engineering. Reproduced with permission.^22^ Copyright 2019, Wiley.

(12) σ  =  neμ

(13) $$S\;=\frac{8\pi^{2}k_{\text{B}}^{2}}{3e\hbar^{2}}\;m^{\text{*}}T{\left(\frac{\pi }{3n}\right)}^{2/3}$$

(14) $$\kappa_{\text{e}}=\;\;L\sigma T$$

where *e* is the electrical charge, μ is the carrier mobility, *k*_B_ is the Boltzmann constant, *ħ* is the Planck constant, m* is the carrier effective mass, and *L* is the Lorenz number. *S*, σ, and κ strongly couple with each other via tuning *n*/*p*. To understand the behaviors of *n*/*p* and *S* in SnSe, the band structure and its density of states (DOS) are introduced. **Figure** **6**a shows a typical calculated band structure of intrinsic α‐SnSe along high‐symmetry points with the generalized gradient approximation (GGA), GGA with spin–orbit coupling (GGA SO), the local‐density approximation (LDA), and LDA with spin–orbit coupling (LDA SO),^213^ respectively. The calculations are based on full‐potential linearized augmented plane wave (FPLAPW) method derived from first principles and semi‐classical Boltzmann theory.^3^ Compared with its simple crystal structure shown in Figure 5, the band structure of SnSe is much complicated, and the overall profiles of the band structures are rather similar with different exchange–correlation functions. The calculated bandgaps from these four functions are ≈0.75, ≈0.71, ≈0.67, and ≈0.63 eV, respectively, indicating that SnSe is a typical semiconductor with a narrow bandgap, which is promising for band manipulation to further improve the electric transport.

To further understand the plotted band structure of SnSe, Figure 6b shows corresponding DOS for both total (up) and partial (down) types,^213^ in which a significantly high DOS can be seen close to the Fermi level (*E*_F_), mainly derived from the generally heavy bands near the band edge. Overall dispersion of both valence and conduction bands nearest the gap can be seen, indicating high *S* potentials can be achieved if *E*_F_ can be appropriately moved via doping, which can contribute to high thermopower. These results confirm that SnSe possesses intrinsic high *S*, and has full potentials to tune *n*/*p* to further achieve a high *S* ^2^σ.

#### 2.2.4 Carrier Tuning

Computational results shown in Figure 6a,b based on first principles can obviously benefit to guiding the design of SnSe‐based thermoelectric materials. However, these calculations are complex and time‐consuming to some extent. To simplify the process of exploring appropriate *n*/*p* to achieve peak ZTs, modellings such as the Kane band (KB) model and single parabolic band (SPB) model are introduced.^216^ For the KB model, it is often used to explore the multiband effects and the charge carriers are assumed mainly scattered by acoustic phonons, which is particularly suitable for SnSe. Taking n‐type SnSe as an example, *S* can be described as^14^, ^217^, ^218^

(15) $$S_{i}=\frac{k_{\text{B}}}{e}\;\left[\frac{{}_{\;}^{1}F_{-2}^{1}\left(\eta_{i},\beta_{i}\right)}{{}_{\;}^{0}F_{-2}^{1}\left(\eta_{i},\beta_{i}\right)}-\eta_{i}\right]$$

where indices *i* refer to different bands, η is the reduced Fermi level, and ${}_{\;}^{n}F_{k}^{m}(\eta ,\beta )$ is the generalized Fermi function, expressed as^14^, ^217^, ^218^

(16) $${\;}^{n}F_{k}^{m}\;\left(\eta ,\;\beta \right)=\;\;\int_{0}^{\infty }\left(-\frac{\partial f}{\partial \varepsilon }\right)\varepsilon^{n}{\left(\varepsilon +\varepsilon \beta^{2}\right)}^{m}{\left[{\left(1+2\beta \varepsilon \right)}^{2}+2\right]}^{\frac{k}{2}}\text{d}\varepsilon$$

*L* can be described as^14^, ^217^, ^218^

(17) $$L_{i}={\left(\frac{e}{k_{\text{B}}}\right)}^{2}\;\left[\frac{{}_{\;}^{2}F_{-2}^{1}\left(\eta_{i},\beta_{i}\right)}{{}_{\;}^{0}F_{-2}^{1}\left(\eta_{i},\beta_{i}\right)}-{\left(\frac{{}_{\;}^{1}F_{-2}^{1}\left(\eta_{i},\beta_{i}\right)}{{}_{\;}^{0}F_{-2}^{1}\left(\eta_{i},\beta_{i}\right)}\right)}^{2}\right]$$

Electron concentration *n* can be described as^14^, ^217^, ^218^

(18) ni=Ai−1 Nv2mbi*kBT3/22π2ℏ3 F003/2ηi,βi

where *N*_v_ is the band degeneracy and *A* is the Hall factor expressed as^14^, ^217^, ^218^

(19) $$A_{i}=\frac{3G_{i}\left(G_{i}+2\right)}{{\left(2G_{i}+1\right)}^{2}}\;\frac{{}_{\;}^{0}F_{-4}^{1/2}{\left(\eta_{i},\beta_{i}\right)}_{\;}^{0}F_{0}^{3/2}\left(\eta_{i},\beta_{i}\right)}{{\left[{}_{\;}^{0}F_{-2}^{1}\left(\eta_{i},\beta_{i}\right)\right]}^{2}}$$

where *G* is calculated by^14^, ^217^, ^218^

(20) $$G\;=m_{kx}^{\text{*}}\;/{\left(m_{ky}^{\text{*}}m_{kz}^{\text{*}}\right)}^{1/2}$$

μ can be described as^14^, ^217^, ^218^

(21) $$\mu_{i}=A_{i}\;\frac{2\pi \hbar^{4}eC_{l}}{m_{I_{i}}^{\text{*}}{\left(2m_{b_{i}}^{\text{*}}k_{\text{B}}T\right)}^{3/2}E_{\text{def}}^{2}}\frac{3_{\;}^{0}F_{-2}^{1}\left(\eta_{i},\beta_{i}\right)}{{}_{\;}^{0}F_{0}^{3/2}\left(\eta_{i},\beta_{i}\right)}$$

where *E*_def_ is the deformation potential coefficient and *C*_l_ is the elastic constant for longitudinal vibrations, determined by^14^, ^217^, ^218^

(22) $$C_{\text{l}}=\;\;v_{\text{l}}{}^{2}\rho$$

where *v*_l_ = 2730 m s^−1^ is the longitudinal speed of sound for SnSe.^24^

Although the KB model has a relative high accuracy for predicting the appropriate *n*/*p*, there are considerable pre‐conditions needed for taking calculations, making the KB model very complicated and limiting its applications. As an alternative, the SPB model is more frequently used. For the SPB model, taking p‐type SnSe as an example, *S* can be described as^22^, ^24^, ^48^, ^197^

(23) $$S\;\left(\eta \right)=\;\;\frac{k_{\text{B}}}{e}\cdot \left[\frac{\left(r+\frac{5}{2}\right)\cdot F_{r+\frac{3}{2}}\left(\eta \right)}{\left(r+\frac{3}{2}\right)\cdot F_{r+\frac{1}{2}}\left(\eta \right)}\;\;-\;\;\eta \right]$$

where *r* is the carrier scattering factor and *r* = −1/2 for acoustic phonon scattering and *F*_i_ *(η)* is the Fermi integral expressed as^22^, ^24^, ^48^, ^197^

(24) $$F_{\text{i}}\;\left(\eta \right)=\;\int_{0}^{\infty }\frac{x^{i}}{1+{\text{e}}^{\left(x-\eta \right)}}\;\text{d}x$$

Hole concentration *p* can be described as^22^, ^24^, ^48^, ^197^

(25) $$p\;=\frac{1}{e\cdot R_{\text{H}}}\;=\frac{{\left(2m^{\text{*}}\cdot k_{\text{B}}T\right)}^{\frac{3}{2}}}{3\pi^{2}\hbar^{3}}\;\cdot \frac{{\left(r+\frac{3}{2}\right)}^{2}\cdot F_{r+\frac{1}{2}}^{2}\left(\eta \right)}{\left(2r+\frac{3}{2}\right)\cdot F_{2r+\frac{1}{2}}\left(\eta \right)}$$

where *R*_H_ is the Hall coefficient. μ can be determined by^22^, ^24^, ^48^, ^197^

(26) $$\mu \;=\left[\frac{e\pi \hbar^{4}}{\sqrt{2}{\left(k_{\text{B}}T\right)}^{\frac{3}{2}}}\frac{C_{\text{l}}}{E^{2}{}_{\text{def}}{\left(m^{\text{*}}\right)}^{\frac{5}{2}}}\right]\;\frac{\left(2r+\frac{3}{2}\right)\cdot F_{2r+\frac{1}{2}}\left(\eta \right)}{{\left(r+\frac{3}{2}\right)}^{2}\cdot F_{r+\frac{1}{2}}\left(\eta \right)}$$

*L* is expressed by^22^, ^24^, ^48^, ^197^

(27) $$L\;={\left(\frac{k_{\text{B}}}{e}\right)}^{2}\;\cdot \left\{\frac{\left(r+\frac{7}{2}\right)\cdot F_{r+\frac{5}{2}}\left(\eta \right)}{\left(r+\frac{3}{2}\right)\cdot F_{r+\frac{1}{2}}\left(\eta \right)}\;\;-{\left[\frac{\left(r+\frac{5}{2}\right)\cdot F_{r+\frac{3}{2}}\left(\eta \right)}{\left(r+\frac{3}{2}\right)\cdot F_{r+\frac{1}{2}}\left(\eta \right)}\right]}^{2}\right\}$$

To exemplify, Figure 6c shows *p*‐dependent *S^2^σ*, κ, and ZT of polycrystalline SnSe at 823 K with a fixed κ_l_ of 0.4 W m^−1^ K^−1^ through the SPB model, from which a *p* of ≈3.0 × 10^19^ cm^−3^ can contribute to a peak ZT of ≈1.4, which is competitive to the currently reported polycrystalline SnSe.^3^ It is clear that in order to further enhance the peak ZT, a lower κ_l_ is needed, which is not affected by tuning *n*/*p* to a great extent (only affecting the carrier‐phonon scattering, which is not a dominant factor to determine κ_l_ in SnSe case).^3^, ^219^

#### 2.2.5 Anharmonicity

For further reducing κ_l_, understanding the anharmonic bonding in SnSe becomes critical since it plays a significant role in determining low κ_l_ with strong anisotropy. The anharmonic bonding can be described when an atom is forced to deviate from its equilibrium position during the phonon transportation, and the applied force is no longer proportional to its displacement, thus resulting in imbalanced phonon transport and in turn a strengthening of phonon scattering,^220^ which overall contributes to a low κ_l_.^194^, ^215^, ^221^, ^222^, ^223^, ^224^, ^225^, ^226^, ^227^ It should be noted that all bonds are anharmonic in most of the materials, but the degree of anharmonicity varies from different materials.^228^, ^229^, ^230^, ^231^, ^232^, ^233^ In this regard, SnSe has the high anharmonic degree, which can be used to further reduce κ_l_. For example, inducing point defects (either vacancies or heteroatoms) into the matrix can strengthen the anharmonic bonding, and can apply for appropriate pressure to alter the anharmonicity.^214^, ^234^, ^235^, ^236^ Other calculation work indicated that the coupled instability of electronic orbitals and lattice dynamics is the origin of the strong anharmonicity in SnSe, which causes the ultralow κ_l_.^237^

To verify the strengthen of anharmonicity, since the heat transfer is mainly contributed by the acoustic modes in SnSe,^214^ Figure 6d shows phonon dispersions of α‐SnSe, the red, green and blue lines highlight TA, TA′, and LA modes, respectively, calculated via the Vienna Ab Initio Simulation Package (VASP) with the projector augmented wave (PAW) scheme based on the density functional theory (DFT) in combination with the semiclassical Boltzmann transport theory. Here TA and TA′ refer to the two transverse modes, and LA refers to the longitudinal mode. From the phonon dispersion results, the Grüneisen parameter γ can be achieved and described as^238^

(28) $$\gamma \;=\;\frac{3\beta BV_{\text{m}}}{C_{\text{v}}}$$

where β is the volume thermal expansion coefficient, *B* is the isothermal bulk modulus, *V*_m_ is the molar volume, and *C*_v_ is the isochoric specific heat (mol^−1^), respectively. Generally, large γ represents a strong anharmonicity, and γ = 0 indicates a harmonic bonding, which is an ideal condition. For SnSe, the calculated average γ values along the *a*‐, *b*‐ and *c*‐axes are 4.1, 2.1, and 2.3,^214^ respectively, indicating a significant difference of γ along different three axes, and the considerable anisotropy found in the thermoelectric performance of SnSe (mainly σ, κ, and μ). The much larger γ achieved along the *a*‐axis comes from the stacked SnSe layers bound by a combination of van der Waals forces and a long‐range electrostatic attractions,^209^, ^211^ making it difficult for either phonon or carrier transportations along this direction, which explain why σ and κ measured along this direction having both the lowest values in SnSe single crystals.^12^

To predict the theoretical κ_l_ in pure SnSe single crystals, the first‐principles calculations indicate that the calculated κ_l_ of stoichiometric SnSe at 300 K were 0.53, 1.88, and 1.44 W m^−1^ K^−1^ along the *a*‐, *b*‐ and *c*‐axes,^215^ respectively, as shown in Figure 6e.^215^ Although the calculated κ_l_ of stoichiometric SnSe at 750 K can be reduced to 0.22, 0.76, and 0.66 W m^−1^ K^−1^ along the a‐, b‐, and c‐axes,^215^ respectively, considering that these κ_l_ values are still high, further κ_l_ reductions are needed to achieve lower κ values as well as higher ZT values. Besides, to predict theoretical minimum κ_l_, a much simpler calculation can be expressed as^11^, ^239^

(29) $$\kappa_{\text{lmin}}=\;\;{\left(\frac{\pi }{6}\right)}^{\frac{1}{3}}k_{\text{B}}n_{\text{a}}{}^{\frac{2}{3}}\sum_{i}v_{i}{\left(\frac{T}{\Theta_{i}}\right)}^{2}\;\int_{0}^{\frac{\Theta_{i}}{T}}\frac{x^{3}{\text{e}}^{x}}{{\left({\text{e}}^{x}-1\right)}^{2}}\text{d}x$$

where *n*_a_ is the number density of atoms, *v_i_* is the sound velocity, and Θ*_i_* is Debye temperature calculated by^11^, ^239^

(30) $$\Theta_{i}=\;\;v_{i}\left(\frac{\hbar }{k_{\text{B}}}\right){\left(6\pi^{2}n_{\text{a}}\right)}^{\frac{1}{3}}$$

#### 2.2.6 Phonon Scattering

To reduce κ_l_ in SnSe, except the strengthening of the anharmonicity in SnSe, other phonon scattering sources should be considered, such as lattice strains. To achieve sufficient strains in a SnSe lattice, multidimensional lattice imperfections are needed in the SnSe matrix, acted as phonon scattering sources. Among these imperfections, point defects are typical 0D lattice imperfections, which play significant roles in both strengthening the anharmonic bonding and causing strain fields for phonon scattering.^220^ In fact, point defects can be treated as the fundamental lattice imperfections. For example, the dislocation as a typical 1D lattice imperfection can be treated as a linear disordered arrangement of point defects,^240^ and the grain boundary as a typical 2D lattice imperfection can be treated as a linear disordered arrangement of dislocations (for the case of small‐angle grain boundary mainly composed by edge dislocations).^241^, ^242^, ^243^, ^244^, ^245^, ^246^, ^247^, ^248^ All these lattice imperfections can produce strain fields in SnSe matrix with different degrees, making concerted efforts to scatter phonons and in turn reduce κ_l_.

To evaluate the effects on reducing κ_l_ by different lattice imperfections, a classical Debye–Callaway model is commonly used.^206^, ^249^ In the relaxation time approximation, it can be expressed as^35^, ^206^, ^250^

(31) $$\kappa_{\text{l}}=\;\frac{k_{\text{B}}}{2\pi^{2}v}{\left(\frac{k_{\text{B}}T}{\hbar }\right)}^{3}\;\int_{0}^{\frac{\theta_{D}}{T}}\tau_{\text{c}}\frac{\zeta^{4}{\text{e}}^{\zeta }}{{\left({\text{e}}^{\zeta }-1\right)}^{2}}\text{d}\zeta$$

Here, ζ is defined as

(32) $$\zeta \;=\;\;\frac{\hbar \omega }{k_{\text{B}}T}$$

where ω is the angular frequency. The total relaxation time τ_c_ consists of individual scattering mechanism via Matthiessen's rule^35^, ^206^, ^250^

(33) $$\tau_{\text{c}}=\;{\left(\tau_{\text{N}}{}^{-1}+\tau_{\text{U}}{}^{-1}+\tau_{\text{V}}{}^{-1}+\tau_{\text{I}}{}^{-1}+\tau_{\text{S}}{}^{-1}+\tau_{\text{D}}{}^{-1}+\tau_{\text{G}}{}^{-1}\right)}^{-1}$$

where τ_N_, τ_U_, τ_V_, τ_I_, τ_S_, τ_D_, and τ_G_ refer to normal process, Umklapp process, vacancy scattering, interstitial atom scattering, substitutional atom scattering, dislocation scattering, and grain boundary scattering, respectively. For normal process, there is^206^

(34) $$\tau_{\text{N}}{}^{-1}\approx \;\tau_{\text{U}}{}^{-1}\beta$$

where β is a fitting parameter for normal process. For Umklapp process, there is^35^, ^206^, ^250^

(35) $$\tau_{\text{U}}{}^{-1}\approx \;\frac{\hbar \gamma^{2}}{Mv^{2}\theta_{\text{D}}}\omega^{2}T\text{exp}\left(-\frac{\theta_{\text{D}}}{3T}\right)$$

where *M* is average molar mass of one atom and θ_D_ is the axial Debye temperature. Calculations indicated that there is a relationship between τ_U_^−1^ and phonon frequency parameter *f* as τ_U_^−1^ ∼ *f* ^2^, indicating the phonons in all crystalline materials are scattered through Umklapp scattering. For vacancy scattering, there is^206^

(36) $$\tau_{\text{V}}{}^{-1}\approx \;\frac{\omega^{4}\delta^{3}}{4\pi v^{3}}x\left(1-x\right){\left[-\frac{M_{\text{v}}}{M}-2\right]}^{2}$$

where δ is the dimension of an atom, *x* is the molar ratio of vacancies, and *M*_v_ is the molar mass of the missing atom (vacancy). For interstitial atom scattering, there is^206^

(37) $$\tau_{\text{I}}{}^{-1}\approx \frac{A\omega^{2}}{{\left(\omega^{2}-\omega_{0}^{2}\right)}^{2}}+\;\frac{2\omega^{4}\delta^{3}}{\pi v^{3}}y\left(1-y\right){\left[3.2\gamma \frac{\Delta \delta }{\delta }\right]}^{2}$$

where *A* is a fitting parameter containing the information of interstitial defects concentration and corresponding binding force with surrounding matrix atoms, ω_0_ is the intrinsic resonant frequency of interstitial defects, which is proportional to ≈$\sqrt{K\text{/}M}$ where *K* is the effective force constant, *y* is the molar ratio of interstitial point defects, and Δδ is the dimension change due to the introduction of a point defect. For substitutional atom scattering, there is^206^

(38) $$\tau_{\text{S}}{}^{-1}\approx \frac{\omega^{4}\delta^{3}}{4\pi v^{3}}z\left(1-z\right){\left[{\left(\frac{\Delta M}{M}\right)}^{2}+\varepsilon {\left(\frac{\Delta \delta }{\delta }\right)}^{2}\right]}^{\;}$$

where *z* is the molar ratio of substitutional point defects and ε is a phenomenological factor as a function of the Grüneisen parameter. Point defects scatter mostly high‐frequency phonons (τ_P_^−1^ ∼ *f* ^4^).^251^ As a typical pristine point defect in SnSe semiconductors, Sn vacancies are frequently found in SnSe matrix, making pure SnSe as an intrinsic p‐type semiconductor. Through solvothermal‐based solution route, the Sn vacancy concentration can be further improved, resulting in a composition of Sn_1−_ *_x_*Se, which is a typical off‐stoichiometric compound. Figure 6e shows the calculated κ_l_ of off‐stoichiometric SnSe, which are only 0.25, 0.38, and 0.36 W m^−1^ K^−1^ at 750 K along the *a*‐, *b*‐ and *c*‐axes, respectively. These values are much lower than that of their stoichiometric counterpart.^206^, ^215^ The reduced κ_l_ is mainly derived from the phonon scattering at the strain fields induced by high concentration Sn vacancies.

For grain boundary scattering, there is^35^

(39) $$\tau_{\text{G}}{}^{-1}\approx \frac{v}{L_{\text{G}}}$$

where υ is the Poisson ratio and *L*_G_ is the grain size. Grain boundaries scatter mostly low‐frequency phonons (τ_G_^−1^ ∼ *f* ^0^).^251^ For dislocation scattering, τ_D_ comes from three parts, namely, dislocation core‐induced strain field τ_Dc_, the strain screw dislocation‐induced strain field τ_Ds_, and the edge dislocation‐induced strain field τ_De_, as can be expressed as^252^

(40) $$\tau_{{\text{Dc}}_{}}{}^{-1}\approx \eta_{\text{D}}N_{\text{D}}\frac{V_{0}^{\frac{4}{3}}}{v_{\text{a}}{}^{2}}\omega^{3}$$

(41) $$\tau_{{\text{Ds}}_{}}{}^{-1}\approx \frac{2^{\frac{3}{2}}}{3^{\frac{7}{2}}}\eta_{\text{D}}N_{\text{D}}b^{2}\gamma^{2}\omega \left\{\frac{1}{2}+\frac{1}{24}{\left(\frac{1-2\nu }{1-\nu }\right)}^{2}{\left[1+\sqrt{2}{\left(\frac{v_{\text{T}}}{v_{\text{L}}}\right)}^{2}\right]}^{2}\right\}$$

(42) $$\tau_{{\text{De}}_{}}{}^{-1}\approx \frac{2^{\frac{3}{2}}}{3^{\frac{7}{2}}}\eta_{\text{D}}N_{\text{D}}b^{2}\gamma^{2}\omega$$

where η_D_ is a factor indicating the orientation of temperature gradient with respect to the dislocation line (1 for perpendicular; 0 for parallel; 0.55 for random), *N*_D_ is the dislocation density, *V*_0_ is the volume per atom, *v*_a_ is the average velocity, *b* is the magnitude of Burger's vector, *v*_T_ is the transverse and longitudinal velocity, and *v*_L_ is the longitudinal velocity, respectively. Considering that most of the remaining heat‐carrying phonons have intermediate frequency around 0.63 THz,^3^ These phonons avoid scattering from point defects and boundaries. Instead, dislocations target to scatter the mid‐frequency phonons with both dependences of τ_D_^−1^ ∼ *f* and τ_D_^−1^ ∼ *f* ^3^, which is between those for point defect and boundary scattering,^219^ thus play a dominant role in strengthening phonon scattering. Because dislocations always distribute at grain boundaries,^251^ increasing the boundary density is a suitable strategy to further reduce κ_l_. Considering that advanced solution route such as solvothermal can conveniently realize morphology control of synthesize SnSe crystals through adjusting appropriate kinetic conditions, it is a good choice to achieve this goal.

Introducing inclusions (especially for nanoinclusions acted as 3D lattice imperfections which have high interface density and nanoscale effects) can further reduce κ_l_ by strengthening phonon scattering at the strain fields induced by these inclusions, the inducing of nanoinclusions is a good strategy to further lower κ and in turn enhance ZT, which can be simply realized by solution route.^24^ When there are nanoinclusions in SnSe matrix, we have^100^, ^253^, ^254^

(43) $$\kappa_{\text{l}}=\kappa_{\text{lp}}\;\frac{\tan^{-1}u}{u}$$

where κ_lp_ is lattice thermal conductivity of parent sample (SnSe in this case). The parameter *u* is defined as^100^, ^253^, ^254^

(44) $$u\;={\left(\frac{\pi^{2}\theta_{\text{D}}\Omega }{\hbar v^{2}}\kappa_{\text{lp}}\Gamma \right)}^{\frac{1}{2}}$$

where Ω is the volume per atom and Γ is imperfection‐scaling parameter^100^, ^253^, ^254^

(45) $$\Gamma \;=\Gamma_{\text{M}}\;+\varepsilon \Gamma_{\text{S}}$$

where Γ_M_ is calculated by average sublattice mass and Γ_S_ is calculated by average sublattice ionic radius.

Except inclusions, inducing pores in SnSe is also an effective way to reduce κ_l_. This is because that both strengthened phonon scattering at the boundaries of pores and thermal radiation in these pores contribute to reduce κ_l_.^255^ In fact, for polycrystalline SnSe, porosity as a typical 3D lattice imperfection is commonly found in their structures due to the fact that the sintering techniques cannot perfectly sinter the raw materials into a bulk without any interspace between the grains, which can be seen from their measured mass density from ≈90% to ≈98%.^3^, ^25^ However, calculations indicate that the pores can contribute to the κ_l_ reduction only when the size of pores is reduced to a certain value. To determine the effective size of pores, a “gray medium” approximation under the assumption of complete phonon‐scattering on the interfaces of these interspaces can be described as^154^

(46) $$\kappa_{\text{l}}=\;\;\frac{1-\phi_{n}}{1+\Lambda_{\text{b}}\cdot \left[\frac{3\alpha_{n}\phi_{n}}{2\left(\alpha_{n}+2\right)\cdot d_{n}}\right]}\;\cdot \kappa_{\text{lb}}$$

where *α_n_* is the “shape” parameter of the gamma distribution,^256^ *d_n_* is the average diameter of interspaces, and κ_lb_ is the lattice thermal conductivity of bulk with 100% relative mass density, respectively. The parameter ϕ_*n*_ can be described as^154^

(47) $$\phi_{n}=\;\;\frac{1}{6}\;\pi c_{n}d_{n}^{3}\left[\frac{\left(\alpha_{n}+\;\;1\right)\left(\alpha_{n}+\;\;2\right)}{\alpha_{n}^{2}}\right]$$

where *c_n_* is the number density of interspaces and Λ_b_ is the phonon mean free path calculated by^154^

(48) $$\Lambda_{\text{b}}=\frac{3\cdot \kappa_{\text{lb}}}{C_{\text{v}}v_{\text{a}}}$$

where *C*_v_ is the volume heat capacity and *v*_a_ is the average sound velocity, taken as 1410 m s^−1^ for SnSe.^24^ The calculated results indicate that the κ_l_ can be significantly reduced when the pores are within an average size of <0 nm. If κ_l_ is 0.4 W m^−1^ K^−1^ for polycrystalline SnSe with a relative mass density of 100%, the induced 2% and 5% volume nanopores with an average size of 40 nm can further reduced κ_l_ to 0.3 and 0.2 W m^−1^ K^−1^, thus to design an appropriate nanoporosity structure in SnSe is critical for further improve the thermoelectric performance of SnSe.

Based on these strategies of reducing κ_l_, Figure 6f shows *p*‐dependent ZT at *T* = 823 K for pure polycrystalline SnSe with further reduced κ_l_ values via a combination of multiple strategies discussed above. A high peak ZT of ≈1.85 at 823 K can be obtained when κ_l_ is reduced to 0.2 W m^−1^ K^−1^, indicating that the further reduction of κ_l_ is crucial for achieving high ZT. However, it should be noticed that the predicted peak ZT of ≈1.85 is not its upper limit value due to that the SPB model is based on pure polycrystalline SnSe system, and it is still not accurate to some extent compared with computational calculations based on first principles, which possess a much higher precision. Thus, through appropriate band engineering such as doping and alloying with other compounds, the upper limit of ZT should be definitely enhanced.

### 2.3 Kinetic Condition

A successful synthesis of SnSe crystals through hydrothermal/solvothermal routes is mainly driven by the selection of appropriate kinetic conditions, as discussed above. However, there are various solvents, precursors, surfactants, and catalysts available for a design of hydrothermal/solvothermal routes to fabricate SnSe crystals, making it difficult to achieve targeted SnSe products, such as morphology control and/or doping level. To meet this challenge, an empirical table to summary these conditions is necessary. **Table** **2** provides a comprehensive overview on the synthesis of polycrystalline SnSe through solution‐based route applied to thermoelectrics, including product type, solvent type, Sn source, Se source, dopant and/or inclusion source, surfactant and/or catalyst, pH adjuster, synthesis temperature (K), synthesis time (h), sintering method, sinter pressure (MPa), sinter temperature (K), and sinter time (min), respectively. It is clear that water and EG are the two of the most frequently used solvents, and hydrazine with its hydrate compound are often used to accelerate the chemical reactions during synthesis.^88^, ^89^ NaBH_4_ can be used as subsolvent to dissolve Se when Se is chosen as Se source,^190^ and PVP and/or ethylenediaminetetraacetic acid (EDTA) are occasionally used as surfactant to ensure a high degree of crystallization in SnSe.^93^, ^257^ It should be noted that more attempts are needed to fill the gaps and further improve the table, thus there is full potentials for finding new phenomenon and/or exploring new mechanism when fabricating SnSe through hydrothermal/solvothermal‐based solution method.

**Table 2:** A comprehensive summary on the synthesis of polycrystalline SnSe via solution route. Abbreviations: E, ethanol; EG, ethylene glycol; EA, ethanolamine; EDA, ethylenediamine; EDTA, ethylenediaminetetraacetic; BA, benzyl alcohol; N_2_H_4_, hydrazine; TSC, trisodium citrate; AA, ascorbic acid; AAH, acetic anhydride; O, oleylamine; OA, oleic acid; PD, pentanediol; PT, 1,10‐phenanthroline; PVP, polyvinylpyrrolidone; TA, tartaric acid; CA, citric acid; BTBC, borane‐*tert*‐butylamine complex; TGA, thioglycolic acid; DMPU, 1,3‐dimethyl‐3,4,5,6‐tetrahydro‐2(1*H*)‐pyrimidinone; TOP, trioctylphosphine; SPS, spark plasma sintering; HP, hot‐pressing; CP, cold‐pressing; QDs, quantum dots. Microwave‐assisted synthesis is marked by ^#^

Product	Solvent	Sn source	Se source	Dopant/inclusion source	Surfactant/catalyst	pH adjuster	Synthesis temperature [K]	Synthesis time [h]	Sintering method	Sinter pressure [MPa]	Sinter temperature [K]	Sinter time [min]	Ref.
SnSe	H_2_O	SnCl_2_	Se	–	EDTA + NaBH_4_	KOH	423–443	6–12	CP + HP	2 tons	853	–	93
SnSe	H_2_O	SnCl_2_	SeO_2_	–	N_2_H_4_	NaOH	393, 413, 433, 453	12	SPS	45	673	15	88
SnSe	H_2_O	SnCl_2_·2H_2_O	NaHSe	–	–	NaOH	373	2	HP	60	773	20	91
SnSe	H_2_O	SnCl_2_·2H_2_O	Se	–	–	NaOH	453	5	–	–	–	–	189
SnSe	H_2_O	SnCl_2_·2H_2_O	Se	–	TSC	NaOH	298	–	–	–	–	–	258
SnSe	H_2_O	SnCl_2_·2H_2_O	Se	–	–	NaOH	403	36	SPS	50	693	7	121
Sn_1−_ *_x_*Se	H_2_O	SnCl_2_·2H_2_O	Se	–	–	NaOH	403	36	SPS	50	693	7	17
SnSe	H_2_O	SnCl_2_·2H_2_O	Se	–	–	TA	298	–	–	–	–	–	259
SnSe	H_2_O	SnCl_2_·2H_2_O	Se	–	NaBH_4_	AA	473	24	SPS	30	773	3.3	190
SnSe	H_2_O	SnCl_2_·2H_2_O	Se	–	NaBH_4_	CA	373	2	HP	≈60	773	20	68
SnSe	H_2_O	SnCl_2_·2H_2_O	Se	–	NaBH_4_	NaOH	373	2	SPS	50	from 573 to 923	5	172
SnSe	H_2_O + EG	SnCl_2_·2H_2_O	SeO_2_	–	N_2_H_4_·H_2_O	NaOH	298, 443	3, 12	–	–	–	–	260
SnSe	EG	SnCl_2_·2H_2_O	SeO_2_	–	N_2_H_4_·H_2_O	NaOH	393, 413, 433	12	SPS	50	773	5	89
SnSe	EG	SnCl_2_·2H_2_O	SeO_2_	–	N_2_H_4_·H_2_O	–	455	24	SPS	50	633, 773	6	26
SnSe	EG	SnCl_2_·2H_2_O	Na_2_SeO_3_	InCl_3_·4H_2_O	–	NaOH	503	36	SPS	60	950	5	25
Sn_0.98_Se	EG	SnCl_2_·2H_2_O	Na_2_SeO_3_	–	–	NaOH	503	36	SPS	60	850	5	25
Sn_0.98_Se	EG	SnCl_2_·2H_2_O	Na_2_SeO_3_	–	–	NaOH	503	36	SPS	70	866	5	171
Sn_0.975_Se	EG	SnCl_2_·2H_2_O	Na_2_SeO_3_	–	–	NaOH	503	36	SPS	70	856	5	170
SnSe_1−_ *_x_*	EG	SnCl_2_	CH_4_N_2_Se	–	–	–	453	24	–	–	–	–	192
SnSe	E	SnCl_2_	Se	–	N_2_	–	273	–	–	–	–	–	261
SnSe	EA	SnCl_2_·2H_2_O	Se	–	–	–	473	24	HP	50	923	10	90
SnSe	EDA	SnCl_2_·2H_2_O	Se	–	AAH	–	453	168	–	–	–	–	262
SnSe	PD + TOP ^#^	SnCl_2_·2H_2_O	Se	–	TGA	–	453	0.5	–	–	–	–	263
SnSe	DMPU + TOP	SnCl_2_	Se	–	BTBC	–	513	0.5	–	–	–	–	264
SnSe	BA	SnCl_2_·2H_2_O	SeO_2_	–	PVP + N_2_	–	473	12	–	–	–	–	257
SnSe	OA + TOP	SnCl_4_·5H_2_O	Se	–	N_2_	–	453, 523	1.25	–	–	–	–	265
SnS_0.1_Se_0.9_	H_2_O	SnCl_2_·2H_2_O	Se	Na_2_S	NaBH_4_	–	373	4	SPS	≈60	773	5	28
Sn_1−_ *_x_*Cu*_x_*Se	H_2_O	SnCl_2_·2H_2_O	Se	CuCl	–	NaOH	403	36	SPS	50	693	7	49
Sn_1−_ *_x_*Pb*_x_*Se	H_2_O	SnCl_2_·2H_2_O	Se	PbCl_2_	–	NaOH	403	36	SPS	50	693	7	23
Sn_0.95−_ *_x_*Pb*_x_*Se	H_2_O	SnCl_2_·2H_2_O	Se	PbCl_2_	–	NaOH	403	36	SPS	50	693	7	27
Sn_0.99−_ *_x_*Pb_0.01_Zn*_x_*Se	H_2_O	SnCl_2_·2H_2_O	Se	PbCl_2_ + ZnCl_2_	–	NaOH	403	36	SPS	50	693	7	16
Sn_0.99_Pb_0.01_Se_1−_ *_x_*S*_x_*	H_2_O	SnCl_2_·2H_2_O	Se	PbCl_2_ + S	–	NaOH	403	36	SPS	50	693	7	177
Sn_0.99_Pb_0.01_Se + Se QDs	H_2_O	SnCl_2_·2H_2_O	Se	PbCl_2_	–	NaOH	403	6	SPS	50	693	7	178
SnSe + Ge	H_2_O	SnCl_2_·2H_2_O	Se	GeI_4_		NaOH	403	36	SPS	40	723	–	19
Ag*_x_*Sn_1−_ *_x_*Se	EG	SnCl_2_	SeO_2_	AgNO_3_	N_2_H_4_	NaOH	413	15	SPS	50	500	5	47
SnSe + Te	EG	SnCl_2_	Se	TeO_2_	NaBH_4_ + PVP + N_2_H_4_	NaOH, KOH	378	2	HP	50	753	10	62
SnSe_1−_ *_x_*Te*_x_*	EG ^#^	SnCl_2_·2H_2_O	Na_2_SeO_3_	Na_2_TeO_3_	–	NaOH	503	6	SPS	40	873	5	60
Sn_1−_ *_x_*Cu*_x_*Se	EG	SnCl_2_·2H_2_O	Na_2_SeO_3_	CuCl_2_	–	NaOH	503	36	SPS	60	900	5	48
Cd‐doped SnSe	EG	SnCl_2_·2H_2_O	Na_2_SeO_3_	CdCl_2_	–	NaOH	503	36	SPS	60	850	5	22
SnSb*_x_*Se_1−2_ *_x_*	EG	SnCl_2_·2H_2_O	Na_2_SeO_3_	Sb_2_O_3_	–	NaOH	503	36	SPS	60	850	5	71
Sn_1−_ *_x_*Bi*_x_*Se	EDA	SnCl_2_	Se	Bi 2‐ethyhexanoate + BiCl_3_	NaBH_4_	NaOH	423	2	SPS	50	773	20	75
Sn_0.94_Bi_0.06_Se	C_6_H_14_ + E	SnCl_4_·5H_2_O	SeO_2_	Bi‐neodecanoate	O + PT + N_2_	–	473	–	SPS	40	723	–	266

## 3 Vacancy Engineering

Compared with conventional melting and mechanical alloying routes, hydrothermal/solvothermal‐based solution methods can realize direct control of Sn and/or Se vacancy concentrations to tune *n*/*p* during synthesis, which can be described as “vacancy engineering.” It is a unique advantage for hydrothermal/solvothermal synthesis. In this section, we discuss the fundamental mechanism of hydrothermal/solvothermal‐based syntheses on the control of Sn/Se vacancies, including kinetic conditions, formation energy, and band manipulation. Based on the discussion, we compare the main thermoelectric performances of SnSe synthesized through traditional melting, hydrothermal, and solvothermal routes to emphasize the advantage of the vacancy engineering from the experimental point of view. The controversy on the difference between experimentally measured composition and calculated results of the carrier density for SnSe with vacancies is also discussed.

### 3.1 Vacancy Physics

#### 3.1.1 Carrier Evaluation

As shown in Table 2, due to different physical parameters (such as Mr, ρ, *T*_m_, *T*_b_, ε, *µ*_d_, and *E* ^T^_N_), different solvent used in solvothermal syntheses should result in different SnSe products with different features caused by the different kinetic conditions, such as morphology and size of crystals, yielding rate, and vacancy concentration, which is one of the key factors to directly tune *n*/*p* and further improve the thermoelectric performance of sintered polycrystalline SnSe. To calculate the carrier density, taking the Sn vacancy for example, in a unit cell of SnSe, there are four Sn atoms and four Se atoms, and the volume of the unit cell *V*_c_ can be calculated by

(49) $$V_{\text{c}}=\;\;a\cdot b\cdot c$$

We choose *a* = 1.137 nm, *b* = 0.419 nm, and *c* = 0.444 nm to achieve a calculated *V*_c_ of ≈2.115 × 10^−22^ cm^3^. If there is a vacancy on Sn site in a unit cell, the composition can be described as Sn_0.75_Se (Sn_1−_ *_x_*Se when *x* = 0.25), the missed Sn atom will contribute to extra 2 holes in the system, making *p* = ≈9.456 × 10^21^ cm^−3^. As shown in Figure 6f, an optimized *p* ≈ 3 × 10^19^ cm^−3^ can result in a peak ZT, that correspond to *x* = 0.0008.

**Figure 7:**
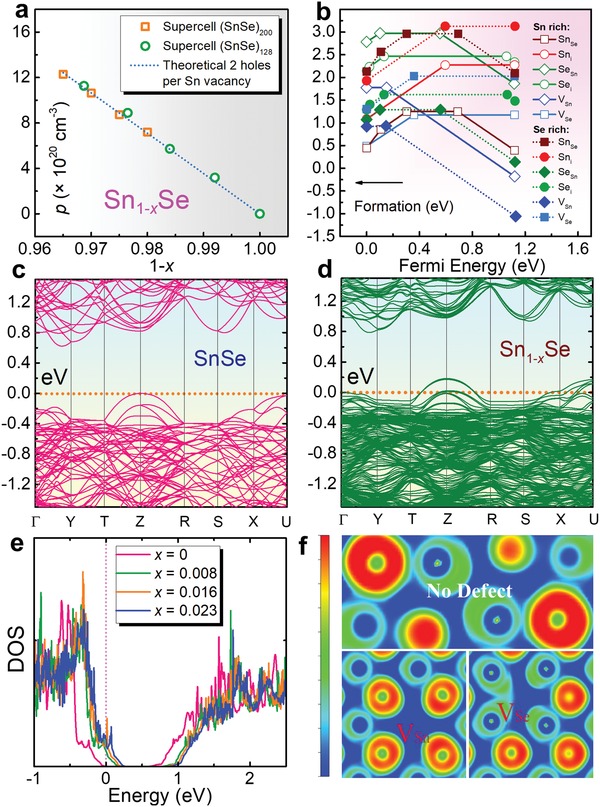
Calculation works focusing on vacancy physics. a) Calculated 1 – *x* dependent *p* for Sn_1−_ *_x_*Se. Reproduced with permission.^197^ Copyright 2019, American Chemical Society. b) Formation energies of point defects in pure SnSe under Se‐rich and Sn‐rich conditions, respectively. Reproduced with permission.^267^ Copyright 2018, American Physical Society. Comparisons of calculated band structures for c) SnSe and d) Sn_1−_ *_x_*Se (here *x* = 0.008). e) Comparisons of DOS for Sn_1−_ *_x_*Se. c–e) Reproduced with permission.^197^ Copyright 2019, American Chemical Society. f) ELF contour maps of normal SnSe, SnSe with Sn and Se vacancies. Reproduced with permission.^271^ Copyright 2018, IOP Publishing.

Advanced computational study based on first principles can calculate the carrier density with a much higher accuracy, according to^197^

(50) $$p\;=\int_{-\infty }^{\text{VBM}}\left(1-f\right)\text{DOS}\left(E\right)\text{d}E$$

(51) $$f\;=\frac{1}{1+\text{exp}\left(\frac{E-E_{\text{F}}}{k_{\text{B}}T}\right)}$$

where VBM is the valence band maxima. The calculated results through DFT with both supercells of 2 × 4 × 4 (256 atoms) and 2 × 5 × 5 (400 atoms) and traditional theoretical evaluation are compared in **Figure** **7**a, from which a typical linear relationship between *p* and 1 − *x* can be seen, which is useful to guide the experimental design for hydrothermal/solvothermal‐based solution route.

#### 3.1.2 Formation Energy

To achieve targeted vacancy concentration, a key point is to adjust the kinetic conditions to meet the requirement of formation energy of these vacancies, simply because all types of point defects including vacancies need energy to form in SnSe matrix. To evaluate the formation potentials of targeted vacancies, first‐principles calculations based on DFT were carried out.^267^, ^268^, ^269^ Figure 7b shows formation energies of all types of point defects in pure SnSe under Se‐rich and Sn‐rich conditions.^267^ For Sn and/or Se vacancies, the calculated results indicate that Sn vacancy (V_Sn_) possess much lower formation energy than Se vacancy (V_Se_), explaining the p‐type electrical transport behavior in pure SnSe. Meanwhile, the results indicate that forming extra Sn vacancies in SnSe matrix is much easier than forming extra Se vacancies, which has been experimentally confirmed.^3^, ^17^, ^25^, ^270^ Besides, the formation of interstitial Sn (Sn_i_) and Se (Se_i_) are much more difficult than that of V_Sn_ and V_Se_, indicating that it is much harder to form interstitial atoms in pure SnSe. In addition, it is of interest to note that the formation energy of Sn substituting Se site (Sn_Se_) is considerably low in Sn‐rich condition, and the formation energy of Se substituting Sn site (Se_Sn_) is also low in Se‐rich condition, indicating that there may be complex composition in real SnSe matrix.

#### 3.1.3 Band Manipulation

Sn/Se vacancies can significantly improve *S^2^σ* by tuning *n*/*p* to their optimized values, which can be explained and guided by the variation of their band structures.^207^, ^213^, ^234^, ^268^, ^269^, ^272^, ^273^, ^274^, ^275^, ^276^, ^277^, ^278^, ^279^, ^280^, ^281^, ^282^, ^283^, ^284^, ^285^, ^286^, ^287^, ^288^, ^289^, ^290^, ^291^, ^292^, ^293^, ^294^, ^295^, ^296^, ^297^, ^298^ Taking Sn vacancy for example, Figure 7c,d compares the calculated band structures for SnSe and Sn_1−_ *_x_*Se (here *x* = 0.008 is used), respectively, and Figure 7e compares DOS based on DFT calculations.^197^ As can be clearly seen, the induced Sn vacancies do not obviously affect the bandgap, but *E*_F_ obviously moves into the valence band, indicating that the Sn vacancy changes SnSe into a degenerate p‐type semiconductor, and in turn resulting in significantly increased *p*.^22^, ^197^ Considering that pristine SnSe possess a low *p* of only ≈2 × 10^17^ cm^−3^,^3^ which is far away from its optimized value of ≈3 × 10^19^ cm^−3^ to achieve a high *S^2^σ*,^3^, ^22^, ^197^ creating more Sn vacancies in SnSe matrix is an effective strategy to improve the thermoelectric performance of pure SnSe.^3^, ^17^, ^22^, ^25^, ^270^, ^299^ For Se vacancies, because their formation energy is much larger than that of Sn vacancies, it is difficult to induce a high concentration of Se vacancies in SnSe matrix to realize high performance n‐type SnSe.^66^

#### 3.1.4 Charge Density

Except the first‐principles‐based computational study discussed above, using electronic localization function (ELF) can also describe the potential charge distribution of SnSe when vacancies are introduced. Figure 7f shows ELF contour maps of SnSe without vacancy (top) and with V_Sn_ (bottom left) and V_Se_ (bottom right), respectively. Compared with the situation with no vacancy, electrons spread over Sn atom for V_Se_, indicating that the extra electrons are attributed to electrons returned back to Sn. Meanwhile, the much higher degree of electron delocalization in the vacancy than the pristine SnSe indicates strong charge transfer, and the contribution of vacancies to σ indicates that proper vacancy design can significantly improve σ. Therefore, securing proper kinetic conditions in hydrothermal/solvothermal synthesis are critical to improve *S* ^2^σ of SnSe‐based thermoelectric materials.

### 3.2 Experimental Verification

#### 3.2.1 Composition Mismatch

To verify the vacancy concentrations in SnSe synthesized through different routes, polycrystalline SnSe fabricated through traditional melting, hydrothermal, and solvothermal routes are compared.^3^, ^24^, ^25^, ^27^ For SnSe fabricated through hydrothermal and solvothermal routes, most of synthesized conditions were same except the solvent, which is water in hydrothermal and EG in solvothermal. Interestingly, the composition of SnSe prepared from traditional melting, hydrothermal, and solvothermal routes were Sn_0.998_Se, Sn_0.992_Se, and Sn_0.981_Se, respectively, evaluated by both the energy‐dispersive spectrometry (EDS) and electron probe microanalyzer (EPMA), although the Sn source and Se source were set as 1:1 during syntheses, indicating that different synthesis routes can result in different Sn vacancy concentrations. The hydrothermal route can result in a much higher Sn vacancy concentration than traditional melting route, derived from the high vapor pressure of water during hydrothermal synthesis in order to lower or meet the vacancy formation energy.^3^, ^25^ Meanwhile, solvothermal route can achieve an even higher Sn vacancy concentration of ≈2%, but the reason is much complex due to the fact that the experimental conditions between water and EG, such as vapor pressure, Mr, ρ, *T*_m_, *T*_b_, ε, *µ*_d_, and *E* ^T^_N_, are all different.

The experimentally measured *p* for Sn_0.998_Se, Sn_0.992_Se, and Sn_0.981_Se are ≈2.4 × 10^17^, ≈1.0 × 10^18^, and ≈1.1 × 10^19^ cm^−3^,^3^, ^24^, ^25^ respectively, indicating significant difference in the *p* values obtained from different synthesis routes. Specially, the *p* achieved in Sn_0.981_Se prepared through a solvothermal route is very close to the calculated best value of ≈3 × 10^19^ cm^−3^ estimated by the SPB model,^24^ indicating that solvothermal route is a promising way to tune *p*. However, it should be noted that even for Sn_0.998_Se fabricated through conventional melting route with a low Sn vacancy concentration of only ≈0.2%, the calculated theoretical value of *p* should be ≈7.6 × 10^19^ cm^−3^, which is much higher than the measured value of only ≈2.4 × 10^17^ cm^−3^, approximately two orders larger than experimental results. There are mainly three reasons accounting for this discrepancy between experimental and calculated results. Frist, considering that even for EPMA that has a higher accuracy than EDS,^25^ there is still ±0.4% error in the measured EPMA results,^25^ thus the precision of experimentally achieved “*x*” value in Sn_1−_ *_x_*Se is limited. Second, the existence of Se vacancies in SnSe matrix is very common in selenides,^66^, ^300^ and the hydrogenation of Sn vacancies is also common seen in selenides,^301^, ^302^ which can neutralize holes and in turn cause experimental errors in both EDS and EPMA due to that H cannot be detected in these techniques. Third, due to the low formation energy of Sn_Se_ and Se_Sn_ as shown in Figure 7b,^267^ substitutions between Sn and Se are commonly seen in the SnSe system. All these phenomena are unavoidable during experiments, resulting in the significant discrepancy between experimental and calculated *p* values.

#### 3.2.2 Electrical Transportation

**Figure 8:**
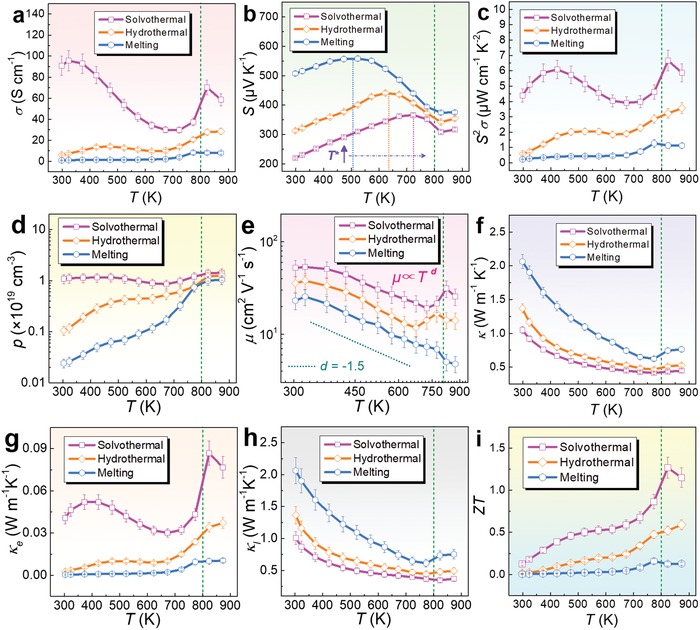
Different solvent on thermoelectric performance of synthesized polycrystalline SnSe. Temperature‐dependent Thermoelectric properties for pure polycrystalline SnSe fabricated via melting, hydrothermal, and solvothermal (EG as solvent) routes,^3^, ^24^, ^25^, ^27^ the properties were all measured along ⊥ direction (perpendicular to sintering pressure): a) σ, b) *S*, c) *S* ^2^σ, d) *p*, e) *µ*, f) κ, g) κ_e_, h) κ_l_, and i) ZT. The green dashed line indicates theoretical phase transition temperature.

To evaluate the influence of Sn vacancies on the thermoelectric performance of SnSe, the main temperature‐dependent properties for Sn_0.998_Se, Sn_0.992_Se, and Sn_0.981_Se along ⊥ direction (perpendicular to sintering pressure) are compared,^22^, ^24^ including σ, *S*, *S* ^2^σ, *p*, μ, κ, κ_l_, and ZT, respectively. **Figure** **8** shows the results, in which the green dashed lines stand for theoretical phase transition temperature at ≈800 K.^86^, ^236^, ^303^, ^304^, ^305^ Figure 8a shows temperature‐dependent σ. Compared with Sn_0.998_Se fabricated through traditional melting routes, Sn_0.981_Se fabricated through solvothermal route has significantly higher σ, derived from extra holes caused by the Sn vacancies, and in turn contributing to higher *p*.^3^, ^25^ Figure 8b shows temperature‐dependent *S*, in which peak *S* values can be achieved at the bipolar‐effect temperature T*. Compared with Sn_0.998_Se fabricated through traditional melting routes, Sn_0.981_Se fabricated through solvothermal route has significantly lower *S*, indicating much higher *p*. Besides, the shift of T* toward a higher *T* also indicates the *p* increase due to the bi‐polar effect.^3^, ^25^ Figure 8c shows the determined *S^2^σ*, from which Sn_0.981_Se fabricated through solvothermal route has the highest *S^2^σ* of ≈6.7 µW cm^−1^ K^−2^ at 823 K due to the fact that its *p* value is much closer to the optimized *p* of 3.0 × 10^19^ cm^−3^ calculated by the SPB model.^306^, ^307^, ^308^

Since *n*/*p* of SnSe is very sensitive to temperature,^3^, ^12^ it should clarify the relationship between *n*/*p* and temperature, so to explore the fundamental mechanism of electrical transport behaviors in SnSe. To achieve this goal, Figure 8d shows *T*‐dependent *p*. With increasing the temperature, thermal excitations can be observed in Sn_0.998_Se fabricated through traditional melting routes and Sn_0.992_Se fabricated through hydrothermal route, contributing to significant *p* improvement to ≈10^19^ cm^−3^ at 823 K.^309^, ^310^ For Sn_0.981_Se fabricated through solvothermal route, its *p* maintained almost constantly with slightly fluctuated for the entire temperature range with high values of ≈10^19^ cm^−3^. Figure 8e shows the corresponding *T*‐dependent μ, which roughly follows μ ∝ *T* ^d^ (power law).^89^, ^311^ It is clear that μ are reduced following μ ∝ *T* ^−1.5^ for all samples, indicating that acoustic‐phonon scattering dominates the scattering mechanism before the phase transition.^89^, ^311^ Meanwhile, although there are extensive vacancies in Sn_0.981_Se fabricated through solvothermal route, which can block and/or scatter carriers and in turn impede the carrier transportation, Sn_0.981_Se still possesses the highest μ because the much higher *p* plays a dominant role in securing a high μ value.

#### 3.2.3 Thermal Transportation

In terms of the thermal transportation, Figure 8f shows the calculated *T*‐dependent κ. As shown in Figure 8f, Sn_0.998_Se fabricated through traditional melting route have much higher κ than SnSe fabricated through solution‐based method for the entire temperature range, even though Sn_0.998_Se possesses much lower κ_e_, as shown in Figure 8g, obtained by κ_e_ = *L·σ·T* via the Wiedemann‐Franz law.^3^ Here *L* of ≈1.5 × 10^−8^ V^2^ K^−2^ was calculated from the SPB model.^306^, ^307^, ^308^ Figure 8h shows the determined *T*‐dependent κ_l_ using κ_l_ = κ – κ_e_. As can be seen, Sn_0.981_Se fabricated through solvothermal route has the lowest κ_l_ (≈0.35 W m^−1^ K^−1^ at 823 K, which is very close to the calculated minimum κ_l_ using the classical Debye‐Cahill model,^239^ from which the calculated κ_l min_ were 0.26, 0.36 and 0.33 W m^−1^ K^−1^ along the *a*‐, *b*‐ and *c*‐axes,^12^, ^277^ respectively), derived from a high concentration of Sn vacancies which can cause larger lattice strains in SnSe matrix. Consequently, it strengthens the phonon scattering and in turn reduce κ_l_. The high κ_l_/κ ratio of >≈70% in all samples indicate that phonon transport is significant for κ in SnSe. Figure 8i shows the determined *T*‐dependent ZT, from which a peak ZT of ≈1.27 at 823 K can be achieved in Sn_0.981_Se fabricated through solvothermal route, indicating that controlling Sn vacancies is critical for securing a high ZT value for polycrystalline SnSe. It should be noted that these results cannot confirm that solvothermal route is better than hydrothermal route for fabricating thermoelectric SnSe because the factors to affect the vacancy concentration is not only the boiling temperature of solvent, so that further studies are still needed to explore all the keys to achieve a high vacancy level.

Recently, it was reported that inducing Sn vacancy by a solvothermal route can lead to a continuous phase transition for Sn_1−_ *_x_*Se, which is also responsible for their high thermoelectric performance.^171^ For the solvothermally synthesized Sn_0.98_Se with inducing ≈2% Sn vacancies, a much lower phase transition temperature of ≈573 K can be achieved, and a continuous phase transition from ≈573 to ≈843 K can be observed by high‐temperature X‐ray diffraction (XRD) and in situ high‐voltage transmission electron microscopy (HVTEM), leading to a high ZT of ≈1.4 at 823 K where α‐SnSe and β‐SnSe coexisted.^171^ Considering that β‐SnSe with a *Cmcm* space group possess much higher thermoelectric performance than α‐SnSe with a *Pnma* space group due to the different band structures with different bandgaps (≈0.9 eV for α‐SnSe and ≈0.45 eV for β‐SnSe),^3^ the continuous phase transition can definitely benefit the high ZT in a broad temperature range for Sn_1−_ *_x_*Se. DFT calculations also reveal the origin to be the suppression of bipolar thermal conduction in the *Cmcm* phase of Sn_0.98_Se due to the enlarged bandgap.^171^ These results indicate that phase‐related designs are critical for realizing high thermoelectric performance in SnSe‐based thermoelectric materials.

#### 3.2.4 Mechanical Property

Although the doping with alkaline metals Na and/or K achieved by traditional melting routes can also tune *p* and in turn achieve high ZTs in SnSe,^29^, ^33^, ^42^ much weak mechanical properties are often resulted in doped alkaline metals, that limits the applications of polycrystalline SnSe in real thermoelectric devices, mainly caused by the high activities of these alkaline metals.^3^ However, solvothermal is a good choice to meet the goal of simultaneously improving the thermoelectric and mechanical properties. It was reported that at a strain rate of 2.5 × 10^−4^ s^−1^, competitive compressive strength of ≈52.1 and ≈77.0 MPa can be achieved along the ⊥ and // directions for solvothermally synthesized Sn_1−_ *_x_*Se (*x* = 0.025),^170^ respectively, and both of which are very competitive with reported record value of 74.4 MPa achieved by a combustion method.^95^ This value is also comparable to the other commercial thermoelectric materials, such as PbTe and Bi_2_Te_3_.^312^, ^313^ Such outstanding mechanical performance are mainly derived from the Sn vacancy induced dispersion hardening for the grains,^314^, ^315^, ^316^ which is similar to the strengthening mechanism of traditional metals and alloys.^314^, ^315^, ^316^

It should be noted that the compressive strength measured along the ⊥ direction was lower than that measured along the // direction. This is because that along the ⊥ direction, the pellet was easier to crack along the textured grains due to the most significant {100} surfaces of SnSe crystals. At the same time, the grains may also be easier to crack due to the weak van der Waals forces between adjacent Sn–Se layers.^209^ In fact, according to the famous Hall–Petch relationship^317^

(52) $$\delta =\delta_{0}+Kg^{-1/2}$$

where δ is the yield strength of polycrystalline materials, δ_0_ is the yield strength of a single crystal, *g* is the average grain size, and *K* is a constant;^317^ a higher mechanical property may be further achieved when reducing the grain size of SnSe pellets. In this regard, solution method is an especially good choice because it can conveniently achieve the morphology control of synthesized SnSe crystals during synthesis by adjusting corresponding synthesis parameters, contributing to a much smaller *g* in sintered polycrystalline bulks and in turn, a higher δ. However, such a grain refinement may reduce the anisotropy and harm the thermoelectric performance of polycrystalline SnSe along specific directions. Therefore, a balance of thermoelectric and mechanical properties is need for securing a practical candidate.

## 4 Morphology Control

SnSe has a typical orthorhombic and layered crystal structure with strong anisotropy.^3^, ^12^ Since an ideal SnSe‐based thermoelectric material should be polycrystalline SnSe composed by grains with single‐crystal‐like anisotropy and optimized *n*/*p*,^3^ it is necessary to strengthen their anisotropy along the *b*‐ or *c*‐directions. However, traditional fabrication techniques such as melting and mechanical alloying are difficult to achieve this goal due to the impossibility of morphology control in fabricated SnSe products.^3^ On the other hand, hydrothermal/solvothermal‐based solution methods can conveniently control the morphology and/or size of synthesized products by appropriately adjusting the synthesis parameters, thus can be treated as promising routes to achieve high thermoelectric performance in polycrystalline SnSe along specific directions. Besides, to meet the requirement of 1D or 2D thermoelectric microdevices, solvothermal route is also the key solution to achieve this goal by controlling the crystallization type from plate‐like to belt‐like, thus allowing the full potential for applying for the microdevices as thermoelectric generators.

### 4.1 Crystal Growth

#### 4.1.1 Size Control

**Figure 9:**
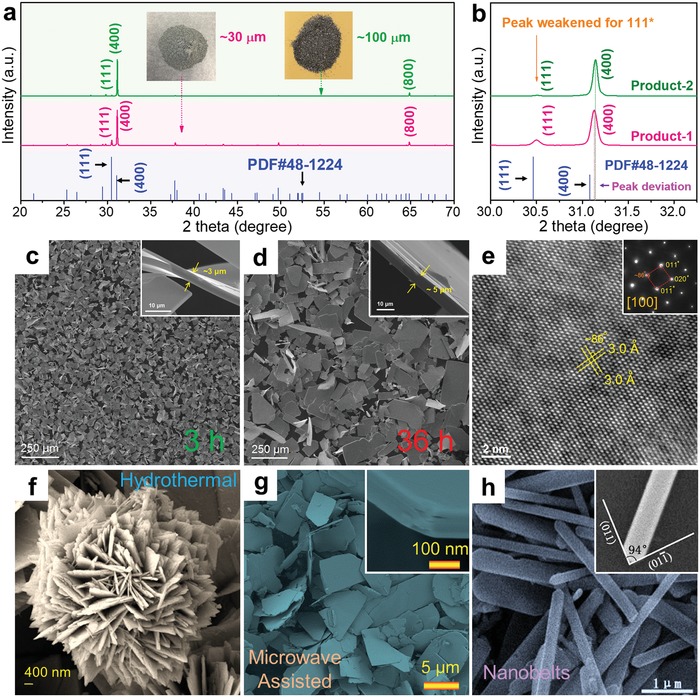
Characterizations on aqueously synthesized SnSe crystals by morphology controlling. a) XRD pattern of solvothermally synthesized SnSe crystals (pink line for products with an average size of ≈30 µm and green line for products with an average size of ≈100 µm) with inserted optical image of synthesized microplates. b) Magnified XRD pattern to see the peak deviation and variation. SEM images of SnSe microplates with inserted SEM images show typical plates for c) products with an average size of ≈30 µm and d) products with an average size of ≈100 µm, respectively. e) HRTEM image and SAED pattern taken from the microplates. a–e) Reproduced with permission.^25^ Copyright 2018, Elsevier. f) Flower‐like SnSe nanoplates by hydrothermal route. Reproduced with permission.^260^ Copyright 2018, American Chemical Society. g) Ultrathin SnSe nanoplates by microwave‐assisted solvothermal route. Reproduced with permission.^60^ Copyright 2017, Royal Society of Chemistry. h) SEM image of SnSe nanobelts by solvothermal route using the amine‐based solvent molecular template with inset showing an individual SnSe nanobelt. Reproduced with permission.^90^ Copyright 2017, Elsevier.

To realize a high anisotropy in the sintered polycrystalline SnSe bulks, to fabricate plate‐like SnSe crystals with a large average size become essential, indicating that the morphology of the fabricated SnSe should be controlled by carefully selecting precursors and their concentrations to avoid suppression of the {001} and {010} planes with high surface energies in SnSe, based on the Bravais law.^47^, ^166^, ^318^, ^319^ Generally, there are many strategies to achieve this goal, such as reducing the amounts of precursors to ensure sufficient space for crystal growth, using appropriate surfactants and/or catalysts to secure complete crystallizations, and just prolong the synthesis time, which is one of the most direct and convenient way to achieve SnSe crystals with a considerable average size.^25^ To exemplify, **Figure** **9**a shows the XRD pattern of solvothermally synthesized SnSe crystals with inset being optical image of synthesized microplates,^25^ the pink line is for crystals with an average size of ≈30 µm fabricated by a 3 h synthesis, and green line is for crystals with an average size of ≈100 µm fabricated by a 36 h synthesis, respectively. It is clear that both crystals show features of metallic luster, and all diffraction peaks can be exclusively indexed as the orthorhombic structured α‐SnSe phase (Standard Identification Card, JCPDS 48‐1224).^25^ Meanwhile, both crystals show significant 400* diffraction peaks, indicating the nature of microplates with significant {100} surfaces, but the 111* diffraction peak from crystals with an average size of ≈100 µm is slightly weaker than that from crystals with an average size of ≈30 µm, indicating a higher anisotropy in synthesized crystals and larger crystal size having more {100} surfaces. Figure 9b shows magnified XRD pattern,^25^ in which both 400* and 111* diffraction peaks shift toward a higher 2θ, indicating a shrink of the unit cell derived from the Sn vacancies in SnSe matrix.

To further study the anisotropy in synthesized SnSe crystals, scanning electron microscopy (SEM) was investigated. Figure 9c,d shows SEM images of SnSe products with average sizes of ≈30 and ≈100 µm,^25^ respectively. All the crystals are plate‐like. The plate‐like morphology explains the significant 400* diffraction peaks found in XRD results found in Figure 9a, which make other peaks weak. Meanwhile, microplates with an average size of ≈100 µm have a much higher dimension size, explaining the weaker 111* diffraction peak found in XRD results. The inset SEM images show typical microplates from a size view, from which microplates with an average size of ≈100 µm have a larger thickness, indicating more complete crystallizations. Both microplates have the same crystal structures, confirmed by the high‐resolution transmission electron microscopy (HRTEM) image with inset corresponding SAED pattern, as shown in Figure 9e. Both HRTEM image and SAED pattern indicate a typical orthorhombic structure of α‐SnSe from the view direction of [100], confirming the most significant surfaces of SnSe microplates are {100}.

#### 4.1.2 Crystallization Type

In some situations, SnSe crystals with a small size are needed, such as using them as the 2D fillers for flexible thermoelectric generators, fabricating microdevices as components, and further improving the mechanical properties of sintered bulks without concerning the performance. In this case, solvothermal route is one of the key solutions to achieve these goals by simply controlling the synthesis parameters, thus possesses full potentials and has attracted increasing attentions in recent years. As discussed in Section 4.1.1, reducing the synthesis time is one of the effective strategies to achieve SnSe crystals with a small average size. However, simply reducing the synthesis time has its own limitations due to the fact that it cannot further reduce the crystal size due to the specific kinetic conditions. In this situation, further modification of the kinetic conditions is needed, and the appropriate selection of solvent is one of the solutions to achieve this goal. Figure 9f shows an SEM image of SnSe nanoplates synthesized by a typical hydrothermal route,^260^ in which the nanoplates have very small sizes, and are agglomerating to form a flower‐like morphology.^320^ Previous studies indicate that the flower‐like morphology prefers to form when the vapor pressure is lower than the standard value (such as the synthesis temperature does not reach the boiling temperature of solvent),^89^ and increasing the precursors amounts can achieve the similar results since there is no enough space for the crystal growth inside the autoclaves.

To achieve SnSe crystals with a much small size, microwave‐assisted solvothermal route is also an effective strategy, as discussed in Section 2.1.3. To exemplify, Figure 9g shows ultrathin SnSe nanoplates synthesized via microwave‐assisted solvothermal route,^60^ and the inset shows one SnSe nanoplate from a side view. It is clear that the synthesized nanoplates have an average size of ≈5 µm with a typical thickness of only 100 nm, that possess full potentials for applying to 2D flexible thermoelectric generators. Meanwhile, there is no agglomeration found in the synthesized nanoplates, which is derived from the homogeneous heating conditions via microwave‐assistance.

In some conditions, 1D SnSe crystals, such as belts, wires, and rods, are needed as microdevices for thermoelectric generation, and hydrothermal/solvothermal routes can achieve this goal by adjusting appropriate synthesis parameters and/or adding templates into the solutions, which can guide the crystal growth of SnSe along specific directions to form 1D SnSe crystals. One typical case can be found in Figure 9h, from which typical SnSe nanobelts can be seen in the SEM image, fabricated by a typical solvothermal route using the amine‐based solvent molecular template.^90^ It is clear that the synthesized nanobelts have uniform size and obvious crystal information. The inset SEM image shows that the measured angle at the end of nanobelt is ≈94°, fitting well with the theoretical angle of (011) and (01$\bar{1}$).^3^ These results indicate that solvothermal route is a promising strategy of realizing different morphology of SnSe for their ultimate applications in microsized thermoelectric devices.

### 4.2 Anisotropy

#### 4.2.1 Anisotropy Strengthening

**Figure 10:**
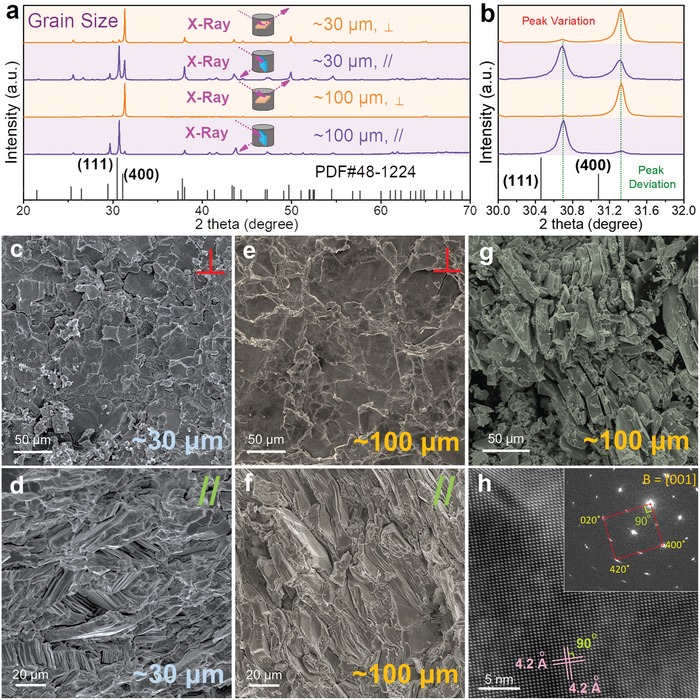
Characterizations on polycrystalline SnSe sintered from solvothermally synthesized SnSe crystals with different size. a) XRD patterns of sintered SnSe pellets with different average grain sizes (≈30 µm and ≈100 µm) measured along both the ⊥ (orange lines) and // (purple lines) directions. b) Magnified XRD patterns to see the peak deviation and variation. SEM images of pellets with an average grain size of ≈30 µm fractured from c) the ⊥ direction and d) the // directions, respectively. SEM images of pellets with an average grain size of ≈100 µm fractured from e) the ⊥ direction and f) the // directions, respectively. a–f) Reproduced with permission.^25^ Copyright 2018, Elsevier. g) SEM image of a cracked pellet by grinding. Reproduced with permission.^170^ Copyright 2019, Wiley. h) HRTEM image and inset SAED pattern of SnSe pellets viewed along the *c*‐axis. Reproduced with permission.^25^ Copyright 2018, Elsevier.

As confirmed by XRD and SEM results shown in Figure 9a–d, there are strong anisotropy in synthesized SnSe microplates through solvothermal method. To confirm such strong anisotropy maintained in the sintered polycrystalline bulks, XRD investigations were often used. **Figure** **10**a shows XRD profiles for samples cut along two orthogonal directions from the sintered pellets with average grain sizes of ≈30 and ≈100 µm, respectively.^25^ The samples have very high relative mass densities of ≈98.5% and ≈98.8% for samples with average grain sizes of ≈30 and ≈100 µm, respectively, derived from the less grain boundaries and interspaces in these sintered pellets compared with traditional melting routes, which can show their intrinsic thermoelectric performance. From Figure 10a, it is clear that both the samples cut along the ⊥ direction show strong 400* diffraction peaks, similar to the XRD pattern taken from the microplates; in contrast, both the samples cut along the // direction shows weak 400* diffraction peaks but strong 111* diffraction peaks, indicating significant anisotropy in the sintered pellets. Figure 10b shows magnified XRD pattern to see the peak deviation and variation of both 400* and 111*,^25^ from which it is clear that samples with an average grain size of ≈100 µm have much weaker 111* peaks along the ⊥ direction, indicating higher anisotropy in these samples compared with their counterparts with an average grain size of ≈30 µm.

In terms of SEM investigations. Figure 10c,d shows SEM images of samples with an average grain size of ≈30 µm fractured from the ⊥ and // directions to the SPS pressure, respectively, and Figure 10e,f shows SEM images of pellets with an average grain size of ≈100 µm fractured from the ⊥ and // directions, respectively. It is clear that distinct fracture features can be found, confirming that the sintered pellets contain a preferred orientation. Meanwhile, larger grain size was observed in Figure 10e,f, and the much more distinct fracture features confirm the sintered pellets containing a much more strengthened anisotropy, fitting well with the XRD results shown in Figure 10a,b. Figure 10g shows an SEM image of a cracked pellet by grinding these pellets into powders, from which the separated SnSe grains still kept an obvious orientation, indicating the considerable anisotropy in sintered SnSe pellets. However, similar to the SnSe microplates, both pellets have the same crystal structures, Figure 10h shows an HRTEM image with inset corresponding SAED pattern, confirming a typical orthorhombic structure of α‐SnSe viewed along the [001] direction.

#### 4.2.2 Performance

**Figure 11:**
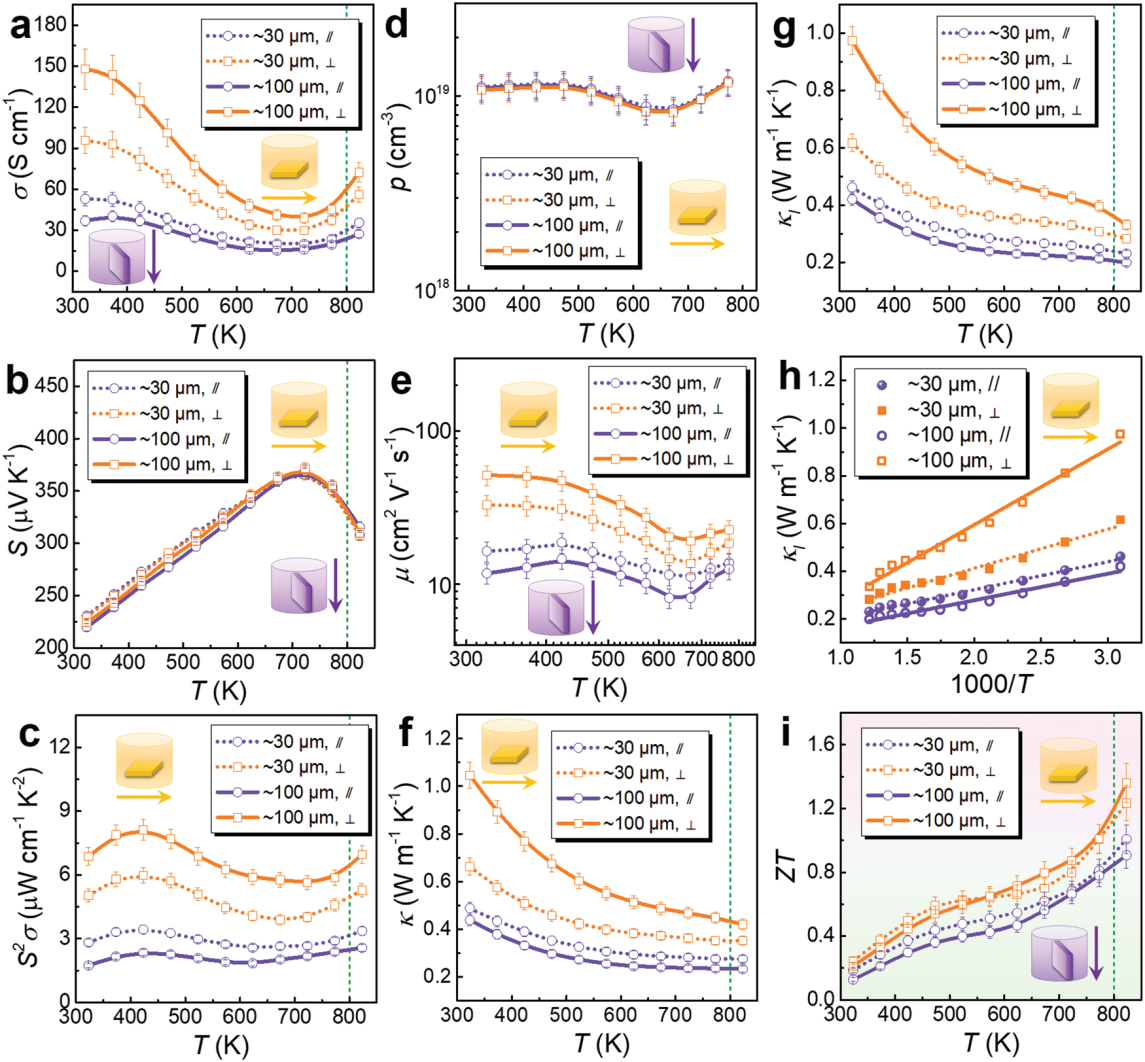
Grain size and anisotropy on thermoelectric performance of solvothermally synthesized polycrystalline SnSe. Thermoelectric properties for polycrystalline SnSe with different grain size along both ⊥ (perpendicular to sintering pressure) and // (parallel to sintering pressure) directions: a) *T*‐dependent σ, b) *T*‐dependent *S*, c) *T*‐dependent *S* ^2^σ, d) *T*‐dependent *p*, e) *T*‐dependent *µ*, f) *T*‐dependent κ, g) *T*‐dependent κ_l_, h) 1000/*T*‐dependent κ_l_, and i) *T*‐dependent ZT. Reproduced with permission.^25^ Copyright 2018, Elsevier.

To evaluate the anisotropy on thermoelectric performance of sintered SnSe pellets fabricated through solvothermal routes, **Figure** **11** shows main *T*‐dependent thermoelectric properties for polycrystalline SnSe with different grain sizes along both the ⊥ and // directions,^25^ including σ, *S*, *S* ^2^σ, *p*, *µ*, κ, κ_l_, 1000/*T*‐dependent κ_l_, and ZT, respectively. Figure 11a shows measured σ_⊥_ and σ_//_. The distinct plots of σ_⊥_ and σ_//_ indicate a strong preferred orientation with σ_⊥_ > σ_//_ over the entire temperature range. Since σ_//_ reflects the conductivity parallel to the *a‐*axis where carriers need to overcome the van der Waals forces between the stacked Sn–Se layers to participate the transportation,^12^ low σ_//_ values are expected. Besides, stronger anisotropy in the pellets with ≈100 µm average grain size can result in a large discrepancy between σ_⊥_ and σ_//_, indicating the strengthen of anisotropy can contribute to high σ along specific directions. Figure 11b shows the measured *S*_//_ and *S*_⊥_,^25^ in which no obvious difference between *S*_//_ and *S*_⊥_ can be found, indicating that *S* is not affected by the preferred orientation to a great extent.^12^ Figure 11c shows the determined (*S^2^σ*)_//_ and (*S^2^σ*)_⊥_,^25^ from which distinct plots of (*S^2^σ*)_//_ and (*S^2^σ*)_⊥_ can be seen, derived from the distinct σ_⊥_ and σ_//_.

To demonstrate the impact of anisotropy on electric transport behaviors of SnSe pellets, Figure 11d plots the measured *p*_//_ and *p*_⊥_,^25^ in which *p*_⊥_ and *p*_//_ are almost same, indicating that *p* has less dependence on anisotropy, similar to *S*. Figure 11e shows determined *µ*_//_ and *µ*_⊥_.^25^ Similar to σ, the distinct plots of *µ*_//_ and *µ*_⊥_ indicate a strong preferred orientation with *µ*_//_ < *µ*_⊥_ over the entire temperature range. For the anisotropy on thermal transport behaviors, Figure 11f plots the achieved κ_//_ and κ_⊥_,^25^ in which the distinct plots of κ_//_ and κ_⊥_ indicate a strong preferred orientation with κ_//_ < κ_⊥_ over the entire temperature range. Figure 11g plots κ_l⊥_ and κ_l//_ for both pellets,^25^ which have the similar results to κ_//_ and κ_⊥_ due to the fact that κ_l_ reflect the nature of crystal structure of SnSe, which is the key reason to cause such a high anisotropy. Figure 11h shows the plots of κ_l//_ and κ_l⊥_ as a function of 1000/*T* for both pellets, form which the linear relationships indicate that the phonon scattering is dominated by the Umklapp phonon scattering rather than the grain size.^21^, ^321^ Figure 11i presents ZT_//_ and ZT_⊥_,^25^ indicating that the strengthening of anisotropy by increase the grain size can achieve a high peak ZT_⊥_ of ≈1.36 at 823 K along specific directions. Thus, strengthening the anisotropy of sintered polycrystalline bulk materials through morphology control through solvothermal routes is an effective strategy to achieve high thermoelectric performance of SnSe.

## 5 Doping

Similar to conventional melting route, hydrothermal/solvothermal‐based solution routes can realize both p‐type and n‐type doping in SnSe by adding various dopants during syntheses. However, there are mainly two distinct features in aqueously induced doping, namely raising the doping limitation (solubility of dopant in SnSe system), and realizing impossible doping through traditional melting route, both need appropriate kinetic conditions to realize during synthesis. Based on these two distinct features, there are more potentials for the band engineering design for polycrystalline SnSe through hydrothermal/solvothermal‐based solution methods.

### 5.1 p‐Type Doping

#### 5.1.1 Breakthrough in Doping Limit

Doping is defined as foreign atoms substitute on Sn and/or Se sites as heteroatoms or interstitial atoms in Sn–Se layers. These foreign atoms provide extra holes or electrons in the system, strengthening the pristine carrier density of p‐type pristine SnSe or making pristine SnSe be n‐type. Strictly speaking, the pure SnSe with high Sn/Se vacancies can also be considered as “self‐doping” because the dopant are vacancies. As shown in Figure 6f, a high *p* of ≈3.0 × 10^19^ cm^−3^ can contribute to a peak ZT value of ≈1.85 in pure SnSe through appropriate structural design and vacancy engineering. However, in order to achieve higher peak ZT (such as ZT > 2), it needs to make a breakthrough on the limitation of ZT by modifying the band structure, especially for the bandgap that directly determine the semiconductor behavior of SnSe. To achieve this goal, doping with foreign atoms is a good choice.

**Figure 12:**
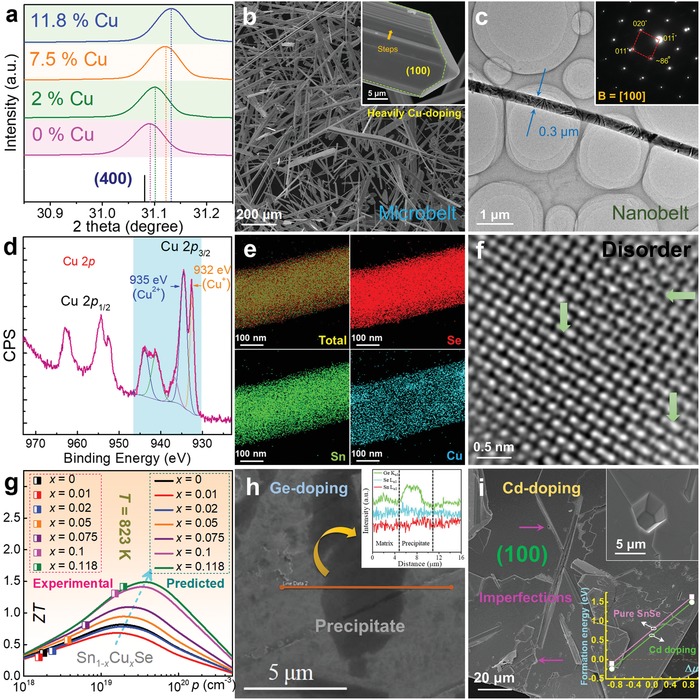
Characterizations on p‐type doping behaviors in aqueously synthesized SnSe. a) Magnified XRD patterns of solvothermally Cu‐doped SnSe products with different doping concentration *x* for Sn_1−_ *_x_*Cu*_x_*Se. b) SEM image of solvothermally synthesized Cu‐doped SnSe microbelts with magnified SEM image inset showing part of a typical microbelt. c) TEM image of a typical microbelt with corresponding SAED pattern inset. d) High‐resolution XPS spectrum for Cu 2p. e) EDS maps of a microbelt. f) STEM‐HAADF image of microbelt to show the disordered arrangement of atoms. g) Comparison of experimental ZTs with predicted plots for Cu‐doped SnSe pellets. a–g) Reproduced under the terms of the Creative Commons Attribution 3.0 Unported Licence.^48^ Copyright 2018, Royal Society of Chemistry. h) SEM image of precipitates in SnSe induced by Ge‐doping with an EDS scan line inset. Reproduced with permission.^19^ Copyright 2019, American Chemical Society. i) SEM image of 3% Cd‐doped SnSe microplates with magnified SEM image inset showing a regular‐shaped crystal imperfection and Sn vacancy formation energy inset showing its reduction by Cd‐doping. Reproduced with permission.^22^ Copyright 2019, Wiley.

SnSe has been demonstrated as a doping‐friendly semiconductor, such as alkali metals (Li,^34^ Na,^29^, ^32^, ^33^, ^34^, ^36^, ^37^, ^38^ and K^29^, ^34^, ^42^) and I‐B group metals (Cu^51^, ^53^, ^322^ and Ag^44^, ^45^, ^46^, ^47^, ^112^) for p‐type doping, and halogens (Cl,^68^, ^73^ Br,^72^ and I^76^) and V‐A group metals (Bi^73^ and Sb^71^) for n‐type doping. For doping in SnSe by a solvothermal route, one of the distinct features from traditional melting route is that solvothermal route can achieve a much higher doping limit (solubility of dopant in SnSe). The breakthrough of doping limit can in turn enhance the thermoelectric performance of polycrystalline SnSe, and heavily Cu‐doped SnSe (Sn_1−_ *_x_*Cu*_x_*Se) is a typical case. **Figure** **12**a shows the magnified XRD results of Sn_1−_ *_x_*Cu*_x_*Se synthesized by a typical solvothermal route using CuO as the Cu source.^48^ The doping limit of Cu in SnSe was enhanced to ≈11.8% determined by both EDS and EPMA,^48^ which is a considerably high value that is difficult to be realized by the traditional melting route.^49^ The fundamental reasons for the raising of doping limit can be attributed to the modified kinetic conditions during solvothermal synthesis, in which the solvent and vapor pressure should be two of the key factors. As shown in Figure 12a, with increasing the doping concentration, the 400* diffraction peaks of Cu‐doped SnSe significantly shifts toward higher 2θ, derived from the shrinkage of the unit cell by inducing Cu into SnSe matrix. Figure 12b shows corresponding SEM image of heavily Cu‐doped SnSe crystals with the doping concentration of 11.8%, from which the synthesized crystals are in the form of microbelts rather than microplates for pure SnSe as shown in Figure 9c,d. The variation of crystal morphology mainly comes from the kinetic conditions change when Cu dopants were introduced into the synthesis, which guide the crystal growth direction for specific surfaces due to the change of surface formation energy,^48^ acted as a template discussed in Figure 9h. Thus, solvothermally doping can be treated as another effective strategy to achieve 1D SnSe crystals, which has full potentials for the applications of microsized thermoelectric devices. Similar results were reported in Ag‐doped SnSe,^47^ from which the doped Ag can change the morphology of SnSe crystals from nanoplates to nanorods. The inset in Figure 12b shows an enlarged SEM image, and shows stepped surfaces parallel to the axial direction of a heavily Cu‐doped SnSe microbelt, caused by the irregular stacking of Sn–Se thinner belts.^48^ Occasionally, some crystals with a much smaller size can be found, as shown in the TEM image in Figure 12c, from which a SnSe nanobelt with a width of only ≈300 nm can be seen. The inset corresponding SAED pattern indicates that the nanobelt has a typical orthorhombic structure viewed along the [100] direction.^48^

To demonstrate the unique doping behavior of Cu in SnSe system through a solvothermal route, Figure 12d shows a high‐resolution X‐ray photoelectron spectroscopy (XPS) spectrum for Cu 2p,^48^ in which strong peaks corresponding to Cu 2p_3/2_ were observed at ≈933 eV, indicating the successful Cu‐doping in SnSe. Interestingly, there were two valence states for Cu ions (Cu^+^ for peak at 932 eV and Cu^2+^ for peak at 935 eV) in SnSe, which is a new finding in the doping behavior of Cu. Figure 12e shows EDS maps for a section of heavily Cu‐doped SnSe nanobelt,^48^ in which all the elements are well distributed, indicating the success doping of Cu in SnSe system. The local nonuniformity of Cu can also be seen, which is also a new finding is also in the Cu‐doping behavior. Figure 12f shows a high‐resolution Cs‐HAADF‐STEM image of heavily Cu‐doped SnSe nanobelt viewed along the *a*‐axis,^48^ from which disordered atom arrangement can be clearly seen as arrows indicate. This phenomenon may come from the local lattice bent by heavily Cu‐doping and/or interstitial Cu atoms, which is an exciting result containing potential new physics in the system.

Figure 12g shows a comparison of experimental ZTs at 823 K with predicted values by SPB model‐based calculations.^306^, ^307^, ^308^, ^323^ It is clear that with increasing the Cu doping level, the measured *p* values are closer to the predicted value (≈3 × 10^19^ cm^−3^), resulting in a peak ZT of ≈1.5, indicating that there are still room for achieving higher ZT if further improving the doping limit of Cu in SnSe. Thus, further studies are needed to explore the best kinetic conditions during solvothermal synthesis to achieve a best doping concentration.

It should be noted that although the appropriate design for hydrothermal/solvothermal synthesis can improve the doping limit, secondary phase may be generated in the solution, which may affect the purity of sintered polycrystalline SnSe bulks. Figure 12h shows a typical case, in which an SEM image shows precipitates in hydrothermally synthesized SnSe induced by Ge‐doping.^19^ The EDS scan line inset clearly shows the Ge‐rich regions in the precipitates.^19^ The induced precipitates may influence on thermoelectric performance in various ways, from which the mechanisms are historically complex. Fortunately, for advanced solution routes, because the formed secondary phase has a much smaller size than SnSe crystals, they can be effectively removed through ultrasonic separation and centrifuging technique after the synthesis,^22^, ^48^, ^71^ which is one of the unique advantages for aqueous synthesis routes compared with traditional melting route, from which the formed secondary phases are hard to be totally removed.

#### 5.1.2 Realizing New Doping

For hydrothermal/solvothermal‐based solution methods, except the advantage of raising the doping limit in SnSe, another unique characteristic is to realize impossible doping in traditional melting routes, achieved by adjusting appropriate kinetic conditions during syntheses. One typical case is the Cd‐doping, which have not been achieved by traditional melting route yet. However, through appropriate solvothermal synthesis design, Cd‐doped SnSe have been achieved with a doping limit of ≈2.3%. Interestingly, the doped Cd in SnSe matrix can further reduce the Sn vacancy formation energy from 1.63 to 1.50 eV confirmed by first principle‐based DFT calculations,^22^ as shown in the inset of Figure 12i, benefitted from the different atomic/ionic size of Cd/Cd^2+^ (0.166/0.095 nm) compared to Sn/Sn^2+^ (0.151/0.112 nm).^324^ The reduction of Sn vacancy formation energy can result in a high cation vacancy of ≈2.9% in SnSe, contributing to an appropriate *p* of ≈2.6 × 10^19^ cm^−3^, which is very close to the best value of ≈3 × 10^19^ cm^−3^ calculated using a SPB model, and in turn an improved *S^2^σ* of ≈6.9 µW cm^−1^ K^−2^ at 823 K,^22^ indicating that solvothermally Cd‐doping can effectively improve the electrical transport performance of polycrystalline SnSe.

To illustrate effect of Cd‐doping on SnSe crystal growth, Figure 12i shows a typical SEM image of a SnSe microplate with a (100) surface through solvothermally Cd‐doping, in which pores and slight crystal bent can be seen.^71^ A typical pore is shown in the inset of Figure 12i in a magnified SEM image, likely caused by the Cd‐doping. These crystal imperfections indicate that doping with foreign atoms can result in different crystal growth of SnSe, which need further clarification in terms of their mechanism behand this novel doping phenomenon.

#### 5.1.3 Band Modulation

**Figure 13:**
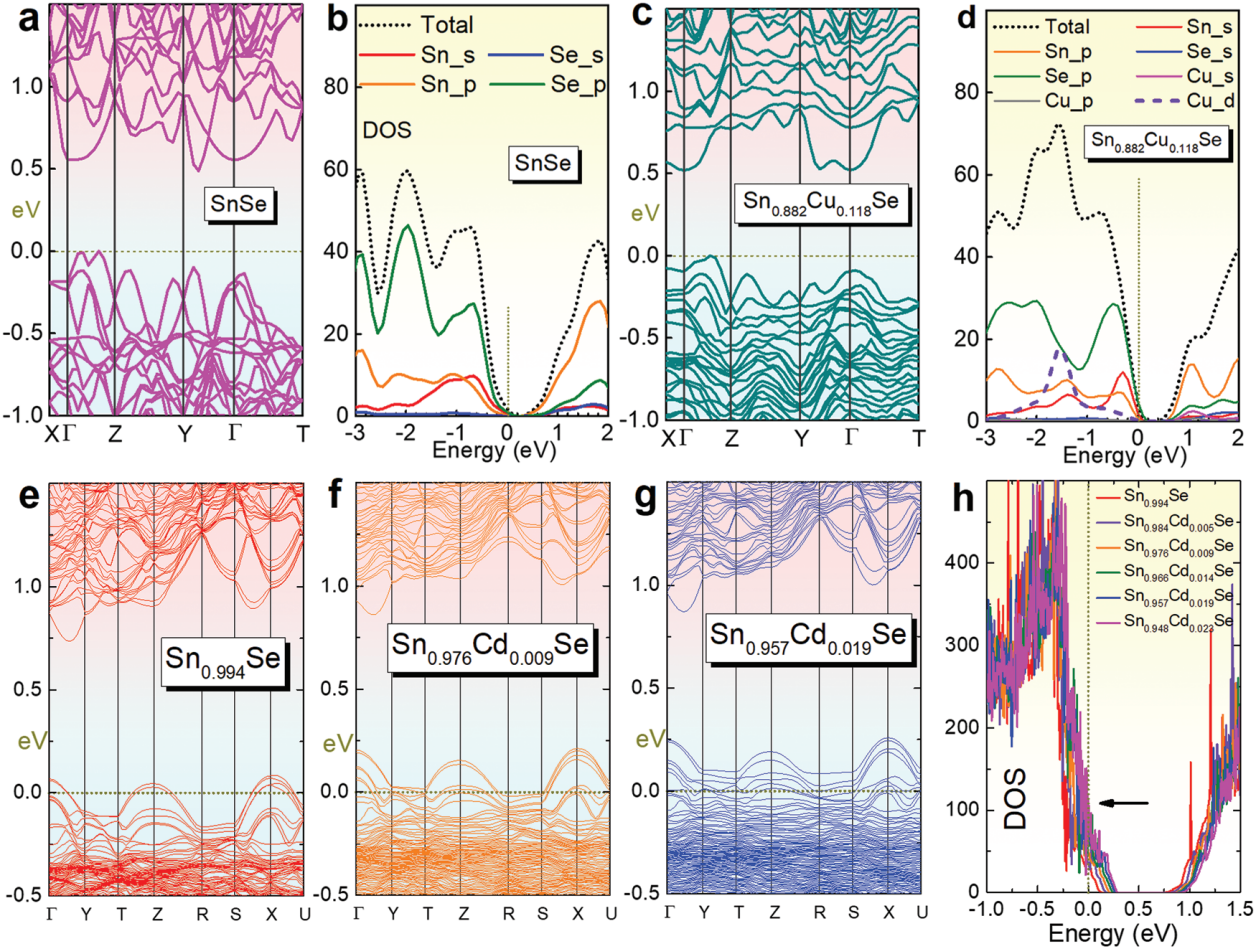
Solvothermal‐driven band manipulations. Calculated a) band structure and b) DOS of SnSe. Calculated c) band structure and d) DOS of Sn_0.882_Cu_0.118_Se. a–d) Reproduced under the terms of the Creative Commons Attribution 3.0 Unported Licence.^48^ Copyright 2018, Royal Society of Chemistry. Calculated band structures of e) Sn_0.994_Se, f) Sn_0.976_Cd_0.009_Se, and g) Sn_0.957_Cd_0.019_Se, and h) comparisons of DOS of Sn_0.994_Se, Sn_0.984_Cd_0.005_Se, Sn_0.976_Cd_0.009_Se, Sn_0.966_Cd_0.014_Se, Sn_0.957_Cd_0.019_Se, and Sn_0.948_Cd_0.023_Se, respectively. e–h) Reproduced with permission.^22^ Copyright 2019, Wiley.

To guide the novel doping in SnSe system through hydrothermal/solvothermal routes, the understanding of band modulation is needed. Taking heavily Cu‐doping in SnSe for an example, **Figure** **13**a,b shows calculated band structure and DOS of pure SnSe,^48^ and Figure 13c,d shows calculated band structure and DOS of Sn_0.882_Cu_0.118_Se,^48^ respectively. The VBM are both pinned to 0 eV in energy. For pure SnSe, Figure 13a shows two distinct conduction band minima (CBM) around Y and at the Γ points of the Brillouin zone, respectively. For the valence band, six VBM can be clearly depicted, with two principal ones lying along the Γ‐Z line. For heavily Cu‐doped SnSe, Figure 13c also shows two distinct CBM around Y and at the Γ points of the Brillouin zone. However, for the valence band, the VBM are not as sharp as pure SnSe, and potential band convergence of multiple‐valences was observed after heavily Cu‐doping, which is responsible for the enhanced *S^2^σ*.^3^, ^60^ Besides, through the calculated DOS of SnSe before and after heavily Cu‐doping, it is clear that the doped Cu (mainly by Cu_d) enhance the DOS at valence bands, indicating the increase of *p*, agreeing with the experimental results shown in Figure 12g. Therefore, the heavy Cu‐doping can significantly improve *p* in SnSe and result in an enhanced *S* ^2^σ.^48^

Another typical case is the solvothermally Cd‐doping. Figure 13e–g shows the calculated band structures ofSn_0.994_Se,^22^ Sn_0.976_Cd_0.009_Se,^22^ and Sn_0.957_Cd_0.019_Se,^22^ respectively, and Figure 13h compares the DOS of Sn_0.994_Se, Sn_0.984_Cd_0.005_Se, Sn_0.976_Cd_0.009_Se, Sn_0.966_Cd_0.014_Se, Sn_0.957_Cd_0.019_Se, and Sn_0.948_Cd_0.023_Se,^22^ respectively. As can be seen, the band structures and DOS are almost identical with and without Cd replacing Sn, due to the same valence state of Cd^2+^ and Sn^2+^. However, with increasing the Cd doping concentration, the Fermi level moves into the valence band, making the material a degenerate semiconductor, similar to the results shown in Figure 7d,e caused by vacancy engineering. This is because the substituting of Cd on Sn sites can result in a reduction of Sn vacancy formation energy, as shown in Figure 12i, which further improve the cation vacancy concentration. Consequently, exploring the band modulation is an effective approach to guide the solvothermal synthesis design and explain the physical reasons behand the novel solvothermal doping behaviors in SnSe.

### 5.2 n‐Type Doping

#### 5.2.1 Pnictogen

Compared with p‐type doping, to realize n‐type doping is historically difficult in SnSe because pristine SnSe is a natural p‐type semiconductor, derived from the much lower Sn vacancy formation energy than Se in SnSe system, as shown in Figure 7b. To realize n‐type doping in SnSe, it is necessary to break the Sn–Se bonding and substituting Sn sites with +3 valence state dopants and/or Se sites with −1 valence state dopants, and solvothermal route is one of the effective ways to achieve this goal, derived from the adjustable kinetic conditions such as solvent, pH, and vapor pressure, which all play significant roles in meeting the required energies to realize n‐type doping.

**Figure 14:**
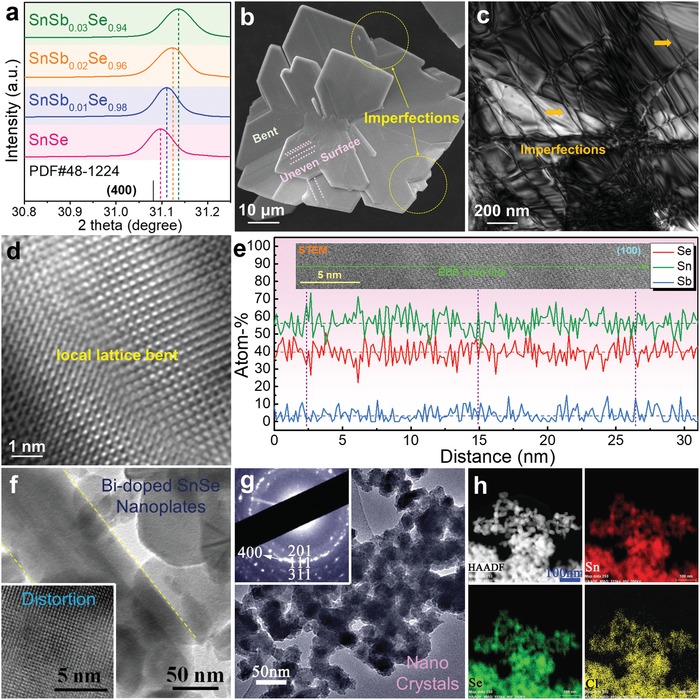
Characterizations on n‐type doping behaviors in aqueously synthesized SnSe. a) XRD patterns (enlarged 400* peaks) of the synthesized SnSb*_x_*Se_1−2_ *_x_* products with different *x* values. b) SEM image of one SnSb_0.03_Se_0.94_ microplate with circled imperfections and crystal bent. c) TEM image of a typical SnSb_0.03_Se_0.94_ plate. d) Corresponding HRTEM image showing the local lattice bent. e) EDS scan line taken from a Cs‐STEM‐HAADF image (inset) viewed along the *a*‐axis. a–e) Reproduced with permission.^71^ Copyright 2018, Wiley. f) TEM image of Sn_1−_ *_x_*Bi*_x_*Se nanoplates with inset HRTEM image showing lattice distortion. Reproduced with permission.^75^ Copyright 2018, American Chemical Society. g) TEM image of Cl‐doped SnSe nanoparticles with corresponding SAED pattern inset. h) Corresponding EDS maps. g,h) Reproduced with permission.^68^ Copyright 2017, Wiley.

**Figure** **14**a shows a magnified XRD pattern of Sb‐doped SnSe products synthesized through a typical solvothermal route using Sb_2_O_3_ as Sb source.^71^ Interestingly, by adding 1%, 2%, and 3% Sb sources in the synthesis, the ratios of Sn, Sb, and Se in synthesized SnSe products were 1:0.01:0.98, 1:0.02:0.96, and 1:0.03:0.94, respectively, determined by both EDS and EPMA,^71^ indicating that the real compositions roughly followed SnSb*_x_*Se_1−2_ *_x_*.^71^ These results suggest that the induced Sb should substitute Se sites rather than Sn sites, resulting in extra Se vacancies in SnSe system due to the different atomic sizes between Se (0.103 nm) and Sb (0.133 nm). From Figure 14a, with increasing the doping concentration, the 400* diffraction peaks significantly shifts toward higher 2θ, derived from the shrinkage of the unit cell by inducing Se vacancies into SnSe matrix due to the Sb doping.^71^

To further study the unique Sb‐doping behaviors in SnSe through solvothermal route, Figure 14b shows a typical SEM image of a SnSb_0.03_Se_0.94_ microplate,^71^ in which crystal imperfection can be seen, including stepped surfaces, breaches, and crystal bent, which should be derived from the Sb‐doping. Figure 14c shows a TEM image of another SnSb_0.03_Se_0.94_ plate,^71^ in which a dislocation network approximately parallel to the {100} surface with averaged spacing of 100 nm is seen, mainly derived from the local lattice distortions with significant strains caused by Sb‐doping. Figure 14d shows an HRTEM image taken from the highly strained area, showing the local lattice bent. Figure 14e shows EDS scan line taken from a Cs‐corrected STEM‐HAADF image (inset) viewed along the *a*‐axis.^71^ Although the Sn and Se signals fluctuate, they follow opposite trend. For Sb, the statistical measurement suggests that more than 70% of Sb peaks/valleys have the same trend as Se, and the calculated average at% values in this case for Sn, Se, and Sb are 56.4%, 39.7%, and 3.9%, respectively, all indicating the substitution nature of Sb in Se sites and Se vacancies.

In fact, Sb is a typical amphoteric metal and can show +3 and −3 valence states in different situations,^325^, ^326^ which is different from Bi since Bi often shows traditional metal behaviors.^15^, ^75^ Therefore, Sb may present different doping behaviors from Bi, and act as Sb^3−^ in SnSe system when appropriate kinetic conditions are achieved through solvothermal synthesis. As a typical V‐A group metals, for Bi‐doping, Sn_1−_ *_x_*Bi*_x_*Se crystals (*x* < 0.04%) can be achieved through a facile solution method when EDA was used as solvent,^75^ as shown in TEM images in Figure 14f, from which Bi‐doped SnSe nanoplates can be seen with significant lattice distortions as inset HRTEM shows, similar to the doping behavior of Sb in SnSe. Thus, there is full potentials for realizing n‐type doping in SnSe by appropriate solvothermal synthesis design.

#### 5.2.2 Halogen

Except pnictogens (V‐A group metals such as Sb and Bi), halogens such as Cl, Br, and I from VII‐A group are also good candidates to realize n‐type doping in SnSe through a solution‐based synthesis. Figure 14g shows a typical TEM image of Cl‐doped SnSe nanocrystals with corresponding SAED pattern inset,^68^ synthesized through a facile solution route, which exploited the nucleophilic nature of the halide anion and the electrophilicity of coordinately unsaturated metal cations at the nanoparticle surface, coupled with the acidic conditions that promote the formation of metal–halide bonds by ligand replacement.^68^ Figure 14h shows EDS maps for Sn (red), Cl (yellow), and Se (green), respectively, indicating a homogeneous doping of Cl. These results indicate that halogens can be successfully doped into SnSe system through appropriate design on solution‐based synthesis.

## 6 Defect Engineering

Inducing nanosized crystal imperfections can result in considerable strains in the lattice, which can significantly scatter the phonons with different frequencies and in turn reduce the κ_l_, thus has been treated as one of the most important strategies to enhance the thermoelectric performance of SnSe. Hydrothermal/solvothermal‐based solution methods can achieve this goal by appropriate adjusting the kinetic conditions during synthesis, thus is very promising with full potentials for achieving low κ_l_. Besides, compared with traditional melting and mechanical alloying routes, hydrothermal/solvothermal synthesis can realize unique “local nanodefect engineering,” which can result in significant local lattice distortions with high‐density strain fields, thus is a power tool to achieve ultralow κ_l_.

### 6.1 Strain in Lattice

**Figure 15:**
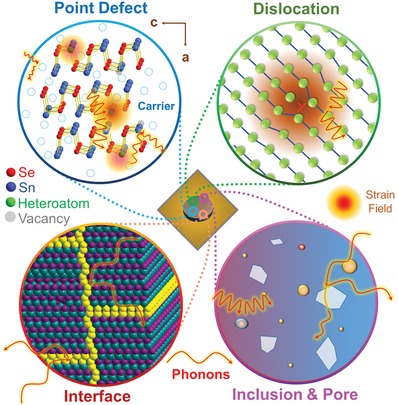
Illustration of potential phonon scattering sources in SnSe, including point defects,^22^ dislocations,^3^ interfaces,^3^ inclusions,^22^ and pores.^22^ Reproduced with permission.^22^ Copyright 2019, Wiley; and reproduced with permission.^3^ Copyright 2018, Elsevier.

As discussed in Section 2.2.6, inducing multidimensional nanodefects in SnSe matrix is critical for secure a low κ_l_. This is because these nanodefects can result in strain fields at a nanoscale, and phonons with different frequencies can be effectively scattered at these strain fields due to the nanosize effect, which explains why only nanosized crystal defects can achieve the goal of reducing κ_l_. For SnSe, there are mainly four types of nanodefects in the matrix, as shown in **Figure** **15**. The 0D nanodefects are mainly point defects, including Sn/Se vacancies and heteroatoms either substituting Sn/Se sites or act as interstitial atoms. Because Sn/Se vacancies and heteroatoms can simultaneously improve *S^2^σ* by tuning appropriate *n* and/or *p* via band engineering and reduce κ_l_ by strengthening the anharmonic bonding and phonon scattering at the strain fields caused by the lattice distortion via inducing these point defects,^3^, ^5^, ^327^ 0D nanodefects have been treated as a fundamental strategy to enhance the thermoelectric performance of SnSe and paid significant attentions in recent years.^3^, ^5^, ^327^ The 1D nanodefects are mainly dislocations, including edge dislocations and screw dislocations. Dislocations can be treated as a linear arranged point defects in SnSe matrix, thus play a significant role in causing considerable strain fields by lattice distortions near these dislocations, strengthening the phonon scattering and in turn reducing κ_l_.^3^, ^5^, ^327^ The 2D nanodefects are mainly interfaces, including grain boundaries, heterojunctions, and stacking faults. These interfaces are commonly composed and/or embedded by high density dislocations, thus play a critical role in contributing to mass strain fields by lattice distortions near grain boundaries, strengthening the phonon scattering and in turn reducing κ_l_.^3^, ^5^, ^327^ Besides, inducing 3D nanodefects such as nanoprecipitates is also an effective way to improve the thermoelectric performance of polycrystalline SnSe by enhancing the electrical transport properties via energy filtering effect and/or reducing the κ_l_ via strengthening the phonon scattering at the strain fields induced by these nanoinclusions, and hydrothermal/solvothermal routes are highly potential routes to achieve this goal.

### 6.2 Scattering Source

#### 6.2.1 Point Defect

**Figure 16:**
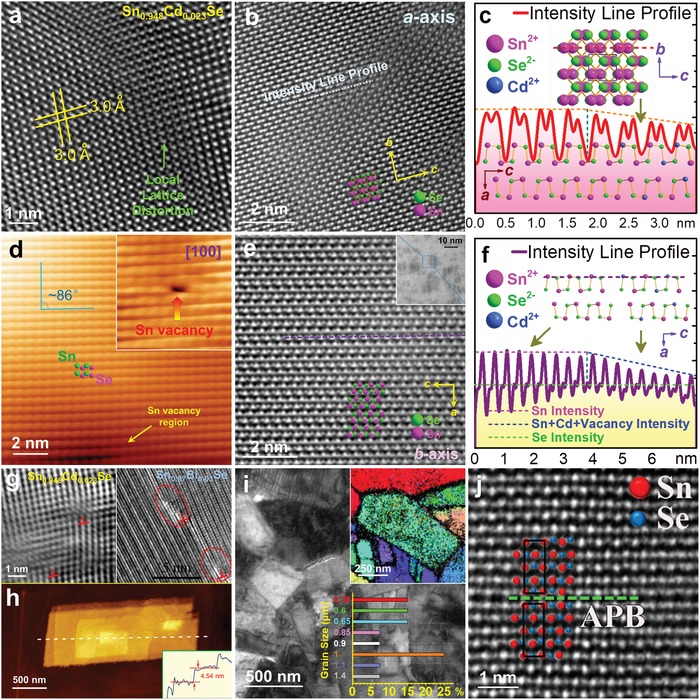
Characterizations on multidimensional crystal imperfections in aqueously synthesized SnSe. a) HRTEM image of Sn_0.948_Cd_0.023_Se microplate showing the localized lattice distortion derived from the point defects via Cd‐doping. b) Corresponding high‐resolution Cs‐STEM HAADF images viewed along the *a*‐axis to see the contrast difference. c) Corresponding intensity line profile taken from (b). d) High‐resolution STM image of pure SnSe microplate surface showing Sn vacancy region and a typical Sn vacancy in the outermost Sn–Se layer inset. e) High‐resolution Cs‐STEM HAADF image of Sn_0.948_Cd_0.023_Se viewed along the *b*‐axis to see the contrast difference, the contrast difference can be clear seen in low‐magnification Cs‐STEM HAADF image inset. f) Corresponding intensity line profile taken from (e). a–f) Reproduced with permission.^22^ Copyright 2019, Wiley. g) Typical dislocations caused by induced heteroatoms, including Sn_0.948_Cd_0.023_Se and Sn_0.97_Bi_0.03_Se, respectively. Reproduced with permission.^75^ Copyright 2018, American Chemical Society. h) AFM image of screw dislocation in SnSe nanoplate with measured step height inset. Reproduced with permission.^257^ Copyright 2016, American Chemical Society. i) TEM image of SnSe mesoscale grains and boundaries with EBSD image inset showing the orientation difference between grains and bar chart inset showing grain size distribution. Reproduced with permission.^23^ Copyright 2016, American Chemical Society. j) High‐resolution Cs‐STEM HAADF image of Sn_0.93_Pb_0.02_Se pellet viewed along the *c*‐axis showing an antiphase boundary (APB). Reproduced with permission.^27^ Copyright 2018, American Chemical Society.

As discussed in Section 6.1, point defects such as vacancies and heteroatoms are effective sources to provide nanosized strain fields for phonon scattering, and hydrothermal/solvothermal routes are good choices to introduce various types of point defects into the SnSe matrix through appropriately adjusting kinetic conditions during synthesis, which can be described as “local nanodefect engineering” and is a unique characteristic compared with traditional melting and mechanical alloying route. **Figure** **16**a shows a typical HRTEM image of Sn_0.948_Cd_0.023_Se microplate,^22^ from which localized lattice distortion derived from the point defects via Cd‐doping can be clearly seen. The point defects include two parts, namely Sn vacancies and Cd substituting Sn sites as heteroatoms, both contributing to the local lattice distortion. Figure 16b shows corresponding Cs‐STEM‐HAADF image taken along the *a‐*axis,^22^ from which the overlays in a normal area show axes and Se atoms in green and Sn atoms in purple. It is clear that a nonuniform structural contrast can be seen, suggesting a typical lattice distortion, likely derived from the local elemental variation.^48^, ^71^ Figure 16c shows the intensity line profile taken along the dashed red line in Figure 16b and shows different peak intensities between different areas,^22^ reflecting the local compositional variations such as Cd replacement and cation vacancies. In fact, solvothermally synthesized SnSe crystals doped with Sb and Bi also exhibited similar local lattice distortions, as shown in Figure 14d,f, indicating the strategy of “local nanodefect engineering” achieved by solvothermal‐based solution route has extensive applicability and practical significance.

To directly observe point defects such as Sn vacancies, scanning tunneling microscopy (STM) was investigated. Figure 16d shows a typical high‐resolution STM image of SnSe microplate surface indexed by relaxed structure models, synthesized via a solvothermal route. In these models, the green and pink balls represent Sn and Se atoms, respectively, and the indexed angle of ≈86° indicates the view direction of [100]. It is clear to see a Sn vacancy induced “sink” on the surface of microplate, derived from the Sn vacancy in the SnSe matrix much close to the surface, resulting in a local lattice distortion with strain field around. Occasionally, Sn vacancies can be directly observed when they are existed in the first layer of Sn–Se (the outermost layer on the surface of microplate), and one typical case is shown in the inset STM image in Figure 16d. Except STM, Cs‐STEM is also a powerful tool to directly observe point defects. Figure 16e shows a high‐resolution Cs‐STEM HAADF image of Sn_0.948_Cd_0.023_Se viewed along the *b*‐axis,^22^ in which the overlays in a normal area show axes and Se atoms in green and Sn atoms in purple. It is clear that a nonuniform contrast can be seen, suggesting local elemental variation,^22^ and the contrast difference can be clear seen in corresponding low‐magnification Cs‐STEM‐HAADF image as inset, described as nanoscale dark domains or “local vacancy domains.” Figure 16f shows intensity line profile taken from Figure 16e,^22^ from which the peak intensities for Se sites keep stable for the entire range, but different peak intensities for Sn sites between normal and dark areas, indicating local elemental variation such as Cd replacing Sn and/or cation vacancies, which can effectively scatter the high‐frequency phonons and in turn contribute to a low κ_l_.^3^, ^48^, ^71^

#### 6.2.2 Dislocation

Similar to point defects, dislocations can lead to significant lattice distortions with strain fields around both the dislocation cores and lines, contributing to a strengthening of phonon scattering and in turn reduce κ_l_.^3^ Computational works indicated that dislocations target to scatter the mid‐frequency phonons which occupy most of the phonons during transportation,^3^ thus play a dominant role in reducing κ_l_.^3^ Generally, inducing point defects can result in dense dislocations, thus hydrothermal/solvothermal routes can be treated as promising strategies to produce dislocations by inducing intensive point defects such as vacancies and/or heteroatoms.

Figure 16g shows two typical cases by HRTEM images, namely Sn_0.948_Cd_0.023_Se^22^ and Sn_0.97_Bi_0.03_Se,^75^ respectively, both synthesized via solution‐based syntheses. For the Cd‐doped SnSe microplates, the edge dislocation was found from a view direction of [100], indicating that the dislocation is parallel to the Sn–Se layers. Significant lattice distortion can be seen around the dislocation, which can effectively scatter the phonons and lead to a reduction of κ_l_. For the Bi‐doped SnSe nanoplates, a wall of edge dislocations can be seen, which compose a typical small‐angle grain boundary. Figure 16h shows an atomic force microscopy (AFM) image of a typical screw dislocation in SnSe nanoplate,^257^ synthesized via a typical solution route. It can be seen that the nanoplate has a single helical pattern and a helical core located at the center, making it like pyramids, derived from its orthorhombic crystal nature. Each basal plane has a rectangular shape and gradually shrinks to the center summit layer by layer when spiraling up, thus the as‐synthesized SnSe nanoplates can be described as screw dislocation‐driven (SDD) growth.^257^ The measured step height of ≈4.54 nm via AFM is shown in the inset figure,^257^ indicating that the thin flakes are approximately 4 layers due to the thickness of the monolayer SnSe being approximately 1 nm.^328^ Even though the strains caused by screw dislocations are much lower than those by edge dislocations confirmed by computational studies,^329^ it is still a good candidate to produce appropriate lattice distortion and benefit to reduce κ_l_.

#### 6.2.3 Interface

Compared with SnSe single crystals, polycrystalline SnSe has interfaces such as grain boundaries in their structures, which can be treated as a “treasury” because the interfaces are the places that high‐density point defects and dislocations (mainly edge dislocations) prior to exist, thus play significant roles in producing massive strain fields around interfaces to strengthen the phonon scattering and in turn reduce κ_l_. Figure 16i shows a typical TEM image of SnSe mesoscale grains, fabricated via a typical hydrothermal route.^23^ The grain boundaries can be clearly seen with a high density. The inset bar chart shows grain size distribution,^23^ and the inset electron backscatter diffraction (EBSD) image shows the orientation difference between grains. Generally, with decreasing the grain size, the grains are inclined to have random orientations, derived from the anisotropy nature of SnSe crystals.

Because SnSe has a typical orthorhombic crystal structure, antiphase boundaries (APB) can be found in SnSe crystals when doping with foreign atoms under specific conditions, which are typical 2D crystal defect described as the disordering of crystallographic planes. APB have considerable potentials for strengthening the phonon scatterings, leading to reduction of κ_l_,^65^, ^330^ similar to the contributions of grain boundaries and/or heterojunctions. Figure 16j shows a high‐resolution Cs‐STEM HAADF image of Sn_0.93_Pb_0.02_Se pellet viewed along the *c*‐axis, synthesized via a typical hydrothermal route.^27^ It is clear that a typical antiphase domain boundary can be seen, which disrupt the normal arrangement of Sn–Se layers along the *a*‐axis, similar to the stacking faults. All these 2D nanosized crystal defects can strengthen the phonon scattering at induced strain fields, contributing to reducing κ_l_. It should be noticed that reducing the grain size (grain refinement) may also weaken the anisotropy of SnSe; however, applying high pressure during sintering process has been demonstrated as an effective way to improve the anisotropy,^61^, ^235^ thus the grain refinement is still a good strategy to achieve high thermoelectric performance in polycrystalline SnSe along both directions.

#### 6.2.4 Inclusion

The contributions of inclusion, especially nanoprecipitates, mainly include two parts. For one thing, the inducing nanoprecipitates can improve the thermoelectric performance of polycrystalline SnSe by enhancing the electrical transport properties via either energy filtering effect or providing extra electrons or holes to improve *n*/*p*; for another, these nanoprecipitates can produce significant strain fields around either the interfaces (heterojunctions) between nanoprecipitate lattice and SnSe matrix or nanoprecipitates themselves, making inducing nanoprecipitates an effective strategy to simultaneously enhance the electrical transport properties and reduce κ_l_, leading to high ZTs in polycrystalline SnSe. To achieve this goal, hydrothermal/solvothermal‐based solution routes are good choices because it can realize simultaneously synthesizing more than two different phases via controlling the size of secondary phases as nanoprecipitates by aligning kinetic conditions of different phases.

**Figure 17:**
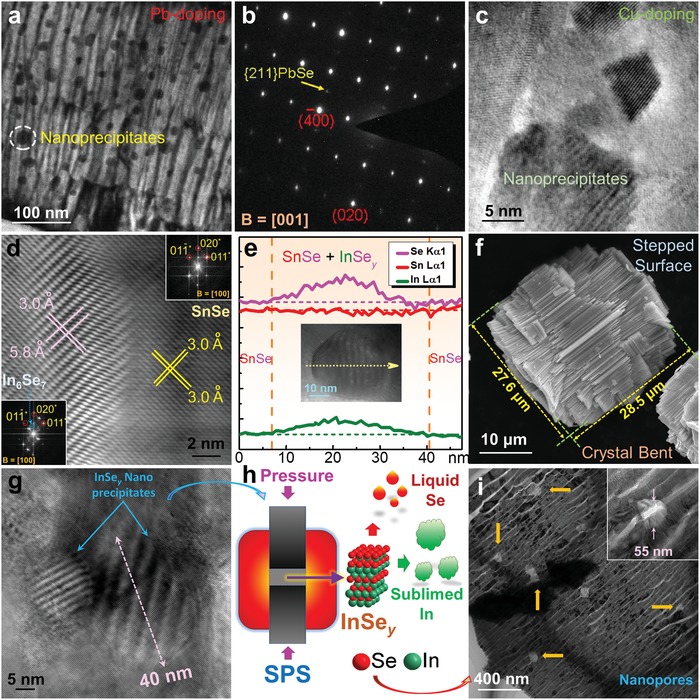
Characterizations on aqueously induced inclusions in SnSe. a) TEM image of PbSe nanoprecipitates in hydrothermally synthesized SnSe. b) Corresponding SAED pattern. a,b) Reproduced with permission.^23^ Copyright 2016, American Chemical Society. c) HRTEM image of Cu‐rich nanoprecipitates in hydrothermally synthesized SnSe. Reproduced with permission.^49^ Copyright 2018, Elsevier. d) Interface between InSe*_y_* nanoprecipitates and SnSe matrix fabricated via a solvothermal route. e) EDS scan line on an InSe*_y_* nanoprecipitate. f) SEM image of SnSe microplates to show the morphology variation by inducing InSe*_y_* nanoprecipitates. g) HRTEM image of two typical InSe*_y_* nanoprecipitates to show their typical sizes. h) illustration of a nanoporosity design. i) TEM image of nanopores in SnSe matrix derived from the decomposition of InSe*_y_* nanoprecipitates during SPS, a typical nanopore is shown in the magnified TEM image inset. d–i) Reproduced with permission.^24^ Copyright 2018, American Chemical Society.

**Figure** **17**a shows a typical TEM image of PbSe nanoprecipitates in hydrothermally synthesized SnSe.^23^ As can be seen, the synthesized PbSe nanoprecipitates are well‐distributed in SnSe matrix with an average size of only ≈10 nm, which are ideal phonon scattering sources. Besides, PbSe is a typical p‐type semiconductor with a much lower direct bandgap of ≈0.27 eV^331^ than SnSe of ≈0.90 eV,^3^ thus can further improve the electrical transport properties of SnSe. Figure 17b shows the corresponding SAED pattern, viewed along the [001] direction of SnSe. Extra pattern with a much weak intensity can be indexed as cubic phase PbSe, indicating that SAED is a useful tool to distinguish secondary phases from SnSe matrix.

Through the good alignment of kinetic conditions for SnSe and secondary phases, nanoprecipitates can be successfully achieved. However, it is not the unique way to produce nanoprecipitates, which can also be achieved when the doping is over the solubility in SnSe, and one typical case can be seen in Figure 17c, from which an HRTEM image of Cu‐rich nanoprecipitates in hydrothermally synthesized SnSe can be seen.^49^ In this case the Cu source used in the synthesis is only <4%, and the doping limit can be further improved to ≈11.8% when the solvent is EG with adjusted synthesis parameters, indicating a higher doping limit can be achieved when the kinetic conditions were appropriately adjusted.

#### 6.2.5 Pore

Pores are special inclusions in SnSe matrix because they are completely hollow. As discussed in Section 2.2.6, inducing pores in SnSe is an effective way to further reduce κ_l_. This is because that both strengthened phonon scattering at the boundaries of pores and thermal radiation in these pores contribute to reduce the κ_l_.^255^ Calculations indicated that the pores can contribute to a reduction of κ_l_ only when the size of pores is reduced to a certain value, and for SnSe the κ_l_ can be significantly reduced when the pores are within an average size of <50 nm.^24^ If the κ_l_ is 0.4 W m^−1^ K^−1^ for polycrystalline SnSe with a relative mass density of 100%, the induced 2% and 5% volume nanopores with an average size of 40 nm can further reduced κ_l_ to 0.3 and 0.2 W m^−1^ K^−1^, thus to design an appropriate nanoporosity structure in SnSe is critical for further improve the thermoelectric performance of SnSe.

To illustrate the nanoporosity design in polycrystalline SnSe, Figure 17d–i shows a typical case. Indium selenides (InSe*_y_*) nanoprecipitates were induced into the matrix of as‐synthesized SnSe microplates via a solvothermal route, and the nanopores in the sintered pellets were achieved via the decompositions of these nanoprecipitates during the sintering process, which is a new concept to achieve nanoporous structure in thermoelectric materials. Figure 17d shows an HRTEM image of a typical boundary between SnSe and induced InSe*_y_* nanoprecipitate with corresponding FFT patterns inset.^24^ It is clear that the lattice of the InSe*_y_* is different from that of SnSe matrix, the corresponding FFT pattern of InSe*_y_* shows extra spots from standard SnSe, which is identified as In_6_Se_7_.^24^ Figure 17e shows the EDS line scans of Se, Sn, and In across a single InSe*_y_* nanoprecipitate, indicating the existence of InSe*_y_*.^24^ The existence of InSe*_y_* nanoprecipitates can also result in significant crystal imperfections, as shown in the SEM image in Figure 17f,^24^ from which considerable surface steps with crystal bent can be seen, mainly derived from the cosynthesized InSe*_y_* nanoprecipitates which impede the crystal growth of SnSe. Figure 17g shows an HRTEM image of two typical InSe*_y_* nanoprecipitates to show their typical sizes of <40 nm.^24^ After the sintering process to decompose these nanoprecipitates under high temperature, high pressure, and high vacuum, as illustrated in Figure 17h,^24^ nanopores can be successfully achieved in polycrystalline SnSe matrix, as shown in TEM image in Figure 17i.^24^ These nanopores can contribute to a high peak ZT of 1.7 ± 0.2 at 823 K, derived from the ultralow κ of 0.24 W m^−1^ K^−1^ achieved at this temperature, indicating that appropriate nanoporosity design can provide a new avenue in achieving high performance in polycrystalline SnSe.

### 6.3 Summary

To provide an overview on the thermoelectric performance of polycrystalline SnSe fabricated via solution‐based syntheses, including traditional aqueous solution, hydrothermal, and solvothermal routes, we comprehensively summarize the reported strategies and properties, including dopant and/or inclusion used during synthesis, semiconductor type, synthesis route, ZT, temperature, σ, *S*, *S* ^2^σ, κ, and *n*/*p*, as shown in **Table** **3**. It is clear that through appropriate design, high thermoelectric performance can be achieved through solution‐based syntheses, which are very competitive compared with traditional melting and mechanical alloying routes, as shown in Figure 1. The achieved properties from other synthesis routes are also provided for comparison.

**Table 3:** A comprehensive summary on the thermoelectric performance of polycrystalline SnSe. Here melting, annealing, mechanical alloying, solid‐state reaction, arc‐melting, aqueous solution, hydrothermal, solvothermal, combustion, electroless plating, spark plasma sintering, hot‐pressing, and cold‐pressing are abbreviated as M, A, MA, SSR, AM, AS, HT, ST, C, EP, SPS, HP, and CP, respectively. The units for *T*, σ, *S*, *S^2^σ*, κ, ρ, and *n*/*p* are K, S cm^−1^, µV K^−1^, µW cm^−1^ K^−2^, W m^−1^ K^−1^, g cm^−3^, and 10^19^ cm^−3^, repsectively. The marked * in *p*/*n* column means the *p*/*n* values were measured at room temperature

Dopant/inclusion	Type	Synthesis	ZT	*T*	σ	*S*	*S* ^2^σ	*Κ*	*n*/*p*	Ref.
Pure	p	ST + SPS	–	300	–	≈160	–	≈1.4	≈1	88
Pure	p	ST + SPS	≈0.6	773	≈28.7	≈339.8	≈3.3	≈0.44	1.02*	89
Pure	p	ST + HP	≈0.8	803	≈16.7	≈346.0	≈2.0	≈0.2	≈1.75	90
Pure	p	AS + HP	–	550	≈35.0	≈338.1	≈4.0	–	–	91
Pure	p	ST + SPS	0.54	550	≈13.9	360	≈1.8	≈0.18	≈0.25*	93
Pure	p	AS + SPS	≈0.47	703	≈30.0	≈300.0	≈2.8	≈0.76	–	172
Pure 2% V_Sn_	p	ST + SPS	1.36	823	72.4	309.9	6.9	0.42	1.48	25
Pure 2% V_Sn_	p	ST + SPS	1.4	823	≈65.0	≈310.0	≈6.5	≈0.38	≈1.5	171
Pure 2% V_Sn_	p	ST + SPS	1.7	823	53.5	307.4	≈5.1	0.24	1.34	24
Pure 2.5% V_Sn_	p	ST + SPS	1.5	823	≈75.0	≈312.5	≈7.4	0.41	≈2.3	170
Pure 5% V_Sn_	p	HT + SPS	2.1	873	≈68.7	≈336.9	≈7.8	≈0.32	≈0.9	17
Pure 10.7% V_Sn_	p	AS + SPS	1.07	885	≈71.9	≈335.7	≈8.1	≈0.66	≈0.48*	26
5% V_Sn_ + 2% Pb	p	HT + SPS	1.4	773	≈30.0	≈321.5	≈3.1	≈0.21	≈0.82	27
10% S	p	AS + SPS	1.16	923	≈50.2	≈333.9	≈5.6	≈0.45	≈0.23*	28
3% Ag	p	ST + SPS	0.8	850	≈90.3	≈266.2	≈6.4	≈0.68	0.9*	47
11.8% Cu	p	ST + SPS	1.41	823	≈55.9	≈315.6	≈5.7	≈0.32	1.95	48
1% Cu	p	HT + SPS	1.2	873	≈35.2	≈310.6	≈3.4	0.2	–	49
1% Zn + 1% Pb	p	HT + SPS	2.2	873	≈50.1	≈328.3	≈5.4	≈0.21	≈0.56*	16
1% Pb + 7% S	p	HT + SPS	1.85	873	≈37.5	≈315.0	≈3.9	≈0.20	0.767*	177
1% Pb + Se QDs	p	HT + SPS	2.0	873	≈32.5	≈425.0	≈5.6	≈0.25	0.686*	178
2.3% Cd + 2.9% V_Sn_	p	ST + SPS	1.7	823	78.8	295.1	6.9	0.33	2.6	22
3% Ge	p	HT + SPS	2.1	873	≈67.9	≈276.5	5.1	≈0.20	4.2*	19
1% Te	p	ST + SPS	1.1	800	≈57.4	≈322.8	≈6.0	≈0.44	≈1*	60
10 mol% Te	p	AS + HP	1.4	790	≈48.1	≈299.8	≈4.3	≈0.25	–	62
1% PbSe	p	HT + SPS	≈1.7	873	≈74.8	≈344.9	≈8.9	≈0.55	0.61*	23
SnSe	p	HT + SPS	1.3	850	≈48.9	≈283.1	≈4.0	≈0.26	0.56*	121
0.6% Cl	n	HT + HP	–	540	≈9.0	≈−264.8	≈0.6	–	0.64*	68
2% Sb + 2% V_Se_	n	ST + SPS	1.1	773	≈39.4	−247.0	≈2.4	≈0.17	3.94	71
3% Bi	n	AS	–	723	≈3.0	≈−387.3	≈0.45	–	<0.01*	75
Pure	p	M + SPS	≈0.5	823	≈31.6	≈355.7	≈4.0	≈0.63	–	78
Pure	p	M + A + SPS	≈0.5	790	≈18.7	≈379.8	≈2.7	≈0.40	≈0.01*	79
Pure	p	M + SPS	–	850	≈66.0	≈300	≈6.0	–	–	80
Pure	p	M + SPS	≈1.0	873	≈49.9	≈408.0	≈8.3	≈0.70	≈0.05*	81
Pure	p	M + HP	1.1	873	≈61.9	≈366.3	≈8.3	≈0.66	0.04*	83
Pure	p	M + HP	0.73	800	≈64.3	≈322.7	≈6.7	≈0.73	<0.01*	84
Pure	p	AM	–	395	–	≈660	–	0.1–0.2	<0.01*	85
Pure	p	AM	–	625	–	1050	–	–	–	86
Pure	p	AM	–	387	≈0.17	≈660.0	≈0.07	≈0.19	–	87
Pure	p	CP + A	≈0.1	772	≈15.3	≈279.8	≈1.2	≈0.75	–	92
Pure	p	MA + SPS	≈0.7	873	≈43.2	≈292.8	3.7	≈0.45	≈0.04*	94
Pure	p	C + SPS	0.5	773	≈25.0	≈368.8	≈3.4	≈0.53	≈0.03*	95
Pure	p	MA + 3D‐Printing	1.7	758	≈22.0	≈340.0	≈2.4	≈0.12	–	332
1% Na	p	M + SPS	0.75	823	≈49.6	≈311.1	4.8	≈0.53	1.0*	32
1% Na	p	M + A + SPS	0.85	800	≈100.4	≈271.5	≈7.4	≈0.50	≈6.5*	33
1% Na	p	M + SPS	≈0.8	800	≈81.2	≈267.2	≈5.8	≈0.50	≈1.5	34
1.5% Na	p	M + MA + HP	≈0.8	773	≈37.9	≈298.8	≈3.4	≈0.33	≈2.1*	37
2% Na	p	SPS	0.87	798	≈56.4	≈288.8	4.7	0.4	3.08*	38
3% Na	p	M + HP	1.3	793	≈149.2	≈253.7	≈9.6	≈0.61	4.34*	40
3% Na	p	SPS	0.82	773	≈65.1	≈280.2	≈5.1	≈0.50	≈2.2	39
0.5% Na + 0.5% Cl	p	SSR + HP	0.84	810	≈79.2	≈228.6	≈4.1	≈0.39	≈3.95*	30
0.5% Na + 0.5% K	p	MA + SPS	1.2	773	≈34.9	≈374.7	≈4.9	0.32	≈7.2*	29
0.5% Na + 1.5% Ag	p	M + A + SPS	0.81	773	≈60.2	≈288.2	≈5.0	0.48	≈2.25*	31
1% Na + 4% Pb	p	M + SPS	≈1.2	773	≈89.4	≈269.7	≈6.5	≈0.45	≈2.8	36
1% Na + 16% Te	p	MA + SPS	0.72	773	≈67.4	≈275.0	≈5.1	≈0.50	–	35
10% S + 3% I	n	M + MA + HP	≈1.0	773	≈10.0	≈−624.5	≈3.9	≈0.30	<0.01*	76
1.5% Cl	p	M	1.1	773	≈25.5	≈399.3	≈4.1	≈0.30	≈0.01*	46
3% Re + 2% Cl + 5% V_Se_	n	M + SPS	1.5	798	≈31.5	≈−430	≈5.8	≈0.30	1.98*	169
0.1% K	p	M + HP	1.11	823	≈58.1	≈281.4	≈4.6	≈0.35	≈0.47*	41
1% K	p	MA + SPS	≈1.1	773	≈18.6	≈421.4	≈3.3	≈0.24	0.92*	42
0.25% As	p	M + HP	≈0.22	723	≈6.0	≈532.9	≈1.7	≈0.56	–	43
8.6% Cu	p	M + HP + A	1.02	300	≈7.5	246	≈0.16	0.4	≈0.11	50
2% Cu	p	M + A + SPS	0.7	773	≈42.4	≈238.6	≈2.4	0.27	18.4*	51
2% Cu	p	M + HP	0.66	813	≈50.0	≈325.6	≈5.3	≈0.65	≈1.6	52
3% Cu	p	M + HP	0.79	823	≈35.0	≈325.1	≈3.7	≈0.39	0.02*	53
Sn_1.005_Se_0.94_Br_0.06_	n	MA + SPS	1.5	783	≈32.0	≈−462.5	≈6.8	0.36	≈0.7	174
10% Br	n	M + HP	1.3	773	≈28.1	≈−400.0	≈4.5	≈0.26	0.93*	69
3% Br	n	M + SPS	1.0	773	≈20.0	≈‐500.0	≈5.0	≈0.36	≈0.006*	179
3% Br + 10% Pb	n	M + SPS	1.2	773	≈35.0	≈−400.0	≈5.6	≈0.37	≈0.02*	70
3% PbBr_2_	n	M + HP	0.54	793	≈36.0	≈−360.0	≈4.7	≈0.72	1.86*	72
0.5% Ag	p	M + EP + SPS	0.85	873	≈60.0	≈310	≈5.6	≈0.59	≈0.1	333
1% Ag	p	M + A + HP	0.6	750	≈45.9	≈344.1	≈5.4	≈0.68	≈0.35*	44
1% Ag	p	M + A + SPS	0.74	823	≈54.8	≈330.9	6.0	≈0.66	1.9*	45
1.5% Ag	p	SSR	1.3	773	≈44.7	≈344.0	≈5.2	≈0.30	≈0.8*	46
1% Ag + 1.5% Ge	p	M + SPS	1.5	793	≈75.0	≈360	≈10.0	≈0.5	0.955*	175
1% Ge	p	M	–	400	–	≈843.2	–	≈0.39	–	55
2% Ge	p	AM	–	773	–	–	–	0.35	–	56
3% Ge	p	M	≈0.15	773	≈3.5	≈377.9	≈0.5	0.27	<0.01*	57
4% Ge	p	M + HP	0.6	823	35.6	≈378.5	5.1	≈0.7	≈0.03*	58
1% In	p	M + HP	0.2	823	≈6.53	≈350.0	≈0.8	≈0.36	≈0.03*	54
1% Zn	p	M + HP	0.96	873	≈74.1	≈328.5	8.0	≈0.73	≈0.45	59
3% Sm	p	M + HP	0.55	823	≈33.6	≈250.0	≈2.1	≈0.32	≈0.01*	63
2% Te	p	HP	–	300	≈9.30	≈331.2	≈1.0	–	–	61
3% Te	p	MA + SPS	≈0.78	823	≈52.5	≈235.0	≈3.4	≈0.3	1.31*	182
6.25% Te	n	M + HP	–	673	≈3.63	≈−276.2	≈0.3	–	≈0.02*	67
10% Te	p	MA + HP	–	550	≈1.6	≈800.0	≈1.0	–	≈0.012*	334
1.0 wt% LaCl_3_	p	MA + SPS	0.55	750	≈15.6	≈350.6	≈1.9	≈0.27	<0.01*	64
0.5% Tl	p	M + HP	0.6	725	≈68.9	≈300.0	≈6.2	≈0.75	–	65
2% V_Se_	n	M + HP	≈0.07	773	≈3.0	≈−380.0	≈0.4	≈0.5	–	66
20% Sb	n	AM	0.3	908	≈100.0	≈−125.0	≈1.0	≈0.46	–	173
0.4% BiCl_3_	n	M + SPS	0.7	793	≈28.9	≈−414.0	≈5.0	≈0.60	1.07*	73
6% Bi	n	M + HP	0.025	723	≈5.5	≈−190.0	≈0.2	≈0.53	–	74
20% Pb + 6% Ti	n	MA + SPS	0.4	773	≈14.8	−450	3.0	≈0.58	<0.01*	77
15% Pb + 5% Cl	n	SSR + MA + A + SPS	1.2	823	≈50.0	≈−362.5	≈6.7	≈0.46	–	176
2.5 vol% C	p	M + HP	1.21	903	≈100.2	≈310.0	9.6	≈0.72	>1.8	96
CNTs + 1.5% Na	p	M + A + SPS	≈0.96	773	≈55.6	≈299.2	≈5.0	≈0.40	≈4*	97
0.2 wt% reduced graphene oxide	p	M + HP	≈0.69	850	≈46.1	≈314.2	≈4.6	≈0.53	–	98
Sn oxide + 1% K	p	MA + SPS	≈1.1	773	≈18.6	≈421.4	≈3.3	≈0.24	0.92*	42
20% SnS	p	MA + SPS	0.64	823	≈26.3	≈332.1	≈2.9	≈0.38	≈0.02*	102
20% SnS	p	M + A + SPS	0.82	823	≈13.8	≈466.9	3.0	≈0.30	≈0.03*	104
10% SnS + 2% Na	p	M + SPS	1.2	793	≈71.0	≈325.0	≈7.5	0.92	4.68*	100
10% SnS + 3% I	n	M + MA + HP	≈1.0	773	≈16.0	≈−500.0	≈4.0	≈0.36	<0.01*	76
15% SnS + 1% Ag	p	M + A + HP	1.67	823	≈25.0	296.4	≈2.2	0.11	0.76*	101
20% SnS + 1 wt% SiC	p	SSR + MA + HP	–	300	≈6.74	≈571.4	≈2.2	–	–	103
20% SnS + 0.5% Ag	p	MA + SPS	≈1.1	823	≈53.4	≈315.1	≈5.3	≈0.40	≈2	105
30% SnS + 3% Na	p	M + HP	1.35	816	≈56.3	≈301.4	≈5.1	≈0.30	≈0.14*	106
SiC	n	M + MA + HP	0.125	300	≈7.3	−581	≈2.5	≈0.60	≈0.58	123
5 vol% K_2_Ti_6_O_13_ whiskers	p	M + HP	0.5	810	≈27.0	≈316.6	≈2.7	≈0.54	–	107
1% Ag_2_S	p	MA + SPS	0.74	773	≈44.3	≈352.0	≈5.5	≈0.58	≈2.43	109
1% Ag_2_S	p	MA + A + SPS	1.13	773	≈44.3	≈315.3	≈4.4	≈0.30	≈0.16*	108
30% AgSbSe_2_	p	M + HP	0.82	842	≈219.98	≈183.08	≈7.4	≈0.75	33.4*	110
Ag_8_SnSe_6_ + 1% Na	p	M + SPS	1.33	773	≈71.2	≈317.0	≈7.2	≈0.42	≈4*	111
10% Sb_2_Se_3_ + 2% Ag	p	M + SPS	0.7	773	≈10.8	≈328.5	≈1.2	≈0.15	4.78*	335
0.5 mol% SnTe + 0.5% Ag	p	M + SPS	1.6	875	≈112.3	≈313.0	≈11.0	0.50	≈0.4*	112
5 wt% Te + 3% Na	p	M + HP	0.8	830	≈89.8	≈274.0	≈6.7	≈0.68	3.51*	113
3.2 wt% MoS_2_ + graphene	p	M + HP	0.98	810	≈70.5	≈258.2	≈4.7	≈0.39	2.49*	114
1.5% MoSe_2_	p	M + SPS	0.5	773	≈21.8	≈428.3	≈4.0	≈0.64	≈2	115
0.15% Cu_2_Se	p	M + SPS	0.51	773	27.3	≈385.0	≈4.0	≈0.58	1.41	117
97% Cu_2_Se	p	M + SPS	1.41	823	≈302.4	≈199.2	12.0	≈0.70	99.3*	118
SnSe_2_ + 2% V_Sn_	p	M + A + SPS	0.61	848	≈48.3	≈308.6	≈4.6	≈0.64	–	116
SnSe_2_ + 6% Cl	n	M + SPS	0.56	773	≈32.0	≈−300.0	≈2.9	≈0.43	≈0.45	122
5% PbSe + 1% Na	p	MA + SPS	≈2.5	773	≈92.9	≈271.7	≈6.9	≈0.20	≈1	13
90% PbSe	n	MA + SPS	1.0	773	≈491.1	≈−183.3	≈16.5	≈1.20	3.5	124
1.5 vol% PbTe	p	M + HP	1.26	880	≈94.7	≈301.2	9.2	≈0.64	1.94	119
Au particles	p	M + SPS	0.72	773	≈20.7	≈380.9	≈3.0	≈0.32	≈0.5	120

## 7 Flexible Generator

Flexible thermoelectric generators are receiving increasing attention due to their capability of directly converting heat into electricity through conformably attaching onto heat sources.^336^, ^337^ Different from solid thermoelectric modules from which the main components are bulk materials, flexible thermoelectric generator with a high flexibility is made of thermoelectric materials with a much smaller average size to ensure the flexibility, and hydrothermal/solvothermal‐based solution routes are key answers to solve this issues through convenient morphology control by adjusting appropriate kinetic conditions during synthesis. The carefully designed nanocrystals synthesized through hydrothermal/solvothermal routes can exhibit significant quantum confinements, which is crucial for improving the thermoelectric performance of flexible generators.

### 7.1 Dimension Evolution

#### 7.1.1 Quantum Confinement

**Figure 18:**
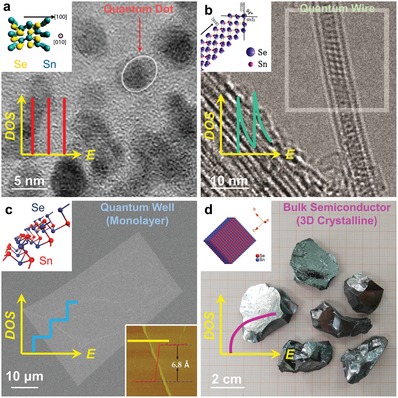
Crystal structures, characterizations, and electronic DOS for multidimensional SnSe nanocrystals: a) quantum dots,^338^, ^339^, ^340^ b) quantum wires,^90^, ^212^, ^338^ c) quantum well (monolayer),^338^, ^341^, ^342^ and d) bulk semiconductors (3D crystals).^24^, ^197^, ^338^ a) Adapted with permission.^339^ Copyright 2011, Elsevier (background); and reproduced with permission.^340^ Copyright 2010, American Chemical Society (inset). b) Adapted with permission.^212^ Copyright 2014, Royal Society of Chemistry (background); and reproduced with permission.^90^ Copyright 2017, Elsevier (inset). c) Adapted with permission.^342^ Copyright 2017, IOP Publishing (background and bottom‐right inset); and reproduced with permission.^341^ Copyright 2015, Royal Society of Chemistry (top‐left inset). d) Adapted with permission.^197^ Copyright 2019, American Chemical Society (background); and reproduced with permission.^24^ Copyright 2018, American Chemical Society (inset).

To achieve a potentially high performance in SnSe‐based flexible thermoelectric generators, the materials should have much low dimensions to ensure the quantum confinement effect.^336^ The quantum confinement refers to when the dimensions of nanocrystals reduce to a certain degree, the nanosized crystals with different sizes can exhibit different bandgap values (normally higher than the larger‐sized crystals) between the conduction band and the valence band, thus have unique electronic properties that differ from larger crystals. The variation of band structures can be simply described from the evolution of DOS, as shown in **Figure** **18**. With decreasing the size of nanoparticles, the quantization of the energy from nanocrystals becomes more obvious, which changes continuous energy bands to discrete energy levels. In other words, when there is at least one dimension of crystals equivalent to the de Broglie wavelength, the motion of electrons in this dimension is limited, the electronic state is quantized, and the continuous energy band is decomposed into discrete energy levels. As a result, a wave function that forms discrete energy levels and standing waves. For thermoelectric materials, such a dimensional reduction at the nanoscale allows new opportunities to quasi‐independently vary *S*, σ, and κ when the length scale is sufficiently small to give rise to quantum‐confinement effects as the number of atoms in any direction (*x*, *y*, or *z*), and potential new physical phenomena may be introduced and these phenomena may also create new opportunities to vary *S*, σ, and κ independently.^338^

#### 7.1.2 Quantum Dot

Quantum dots (QDs) can be described as nanoscale semiconductor particles with a few nanometers in size. Figure 18a shows an HRTEM image of typical SnSe QDs,^338^, ^339^, ^340^ synthesized through a typical solution route by using an electronic accelerator as a radiation source and hexadecyl trimethyl ammonium bromide as a surfactant. The as‐synthesized SnSe QDs have average size of only ≈4 nm with the spherical shape. The QDs exhibited a large direct bandgap of 3.89 eV, greatly blue shifted compared with that of bulk SnSe (≈0.9 eV) due to the significant quantum confinement effect, showing typical blue photoluminescence at ≈420 nm.^339^

Currently, SnSe QDs have been preliminarily applied to sensitized solar cells.^339^, ^343^ For their applications on thermoelectrics, more studied are needed. Nevertheless, SnSe QDs have exhibited amazing bandgap values and high surface activities, thus have full potentials for thermoelectric applications such as flexible thermoelectric generators.

#### 7.1.3 Quantum Wire

Because of the nature of orthorhombic layered structure in SnSe, SnSe quantum wires (QWs) were few reported, and templates are needed during synthesis. Figure 18b shows an HRTEM image of SnSe QWs encapsulated within single walled nanotubes (SWNTs) with diameters below ≈1.4 nm,^212^ from which Sn–Se atom column pairs arranged along a section of an SWNT can be clearly seen. In the case of using tubes as templating, the grown nanostructures are generally polycrystalline, which may affect their properties. Even though the challenge in synthesis restrict the development of SnSe QWs, QWs are still pointed to be very promising for applying to thermoelectrics due to their significant quantum confinement effect and suitable bandgap values of ≈1.40 eV.^344^

#### 7.1.4 Quantum Wall

For SnSe, because of its orthorhombic layered structure, it is much easier to form quantum well, which can be described as monolayer. Figure 18c shows a TEM image of SnSe monolayer,^338^, ^341^, ^342^ the inset AFM image indicates that the thickness of monolayer is only ≈0.68 nm, which is close to the theoretical value of single‐layer SnSe (≈5.749 Å). In fact, SnSe monolayers have been paid significant attentions in recent years, derived from their relative high stability, considerable quantum confinement effect, and high potentials in applying to flexible thermoelectric generators.^3^, ^341^, ^345^, ^346^, ^347^, ^348^, ^349^ Computational works based on DFT indicated that the monolayer is dynamically and thermally stable with a bandgap of 1.28 eV,^341^ and the ZTs can reach 3.27/2.76 along the *b*‐ and *c*‐ directions with optimal *n* at 700 K due to quantum confinement effect.^341^ Other works based on the Boltzmann transport equation also indicated that SnSe monolayer can exhibit high thermoelectric conversion efficiency in p‐type doping, derived from its high *S* ^2^σ and low κ.^348^ These studies indicate that SnSe monolayers are promising candidates for applying to 2D/3D thermoelectrics.

### 7.2 Crystal Design

#### 7.2.1 Particle

**Figure 19:**
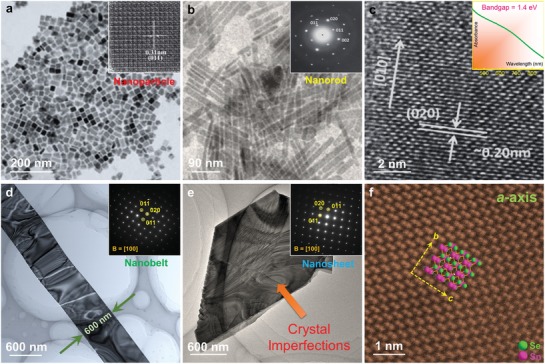
Characterizations on multidimensional SnSe nanocrystals. a) TEM image with HRTEM image inset showing typical SnSe nanoparticles. Reproduced with permission.^350^ Copyright 2011, Royal Society of Chemistry. b) TEM image with SAED pattern inset showing typical SnSe nanorods. c) Corresponding HRTEM image with UV–vis absorption spectrum inset. b,c) Reproduced with permission.^344^ Copyright 2014, J‐STAGE. TEM images with SAED patterns inset showing d) a typical SnSe nanobelt and e) a typical SnSe nanosheet. d) Reproduced under the terms of the Creative Commons Attribution 3.0 Unported Licence.^48^ Copyright 2018, Royal Society of Chemistry. e) Reproduced with permission.^71^ Copyright 2018, Wiley. f) High‐resolution Cs‐STEM‐HAADF image of nanosheets. Reproduced under the terms of the Creative Commons Attribution 3.0 Unported Licence.^48^ Copyright 2018, Royal Society of Chemistry.

**Figure** **19**a shows a TEM image of typical SnSe nanoparticles,^350^ synthesized through a solution route based on oleylamine (OLA) as solvent. The nanocrystals with an average size of ≈20 nm are dispersed uniformly without obvious aggregation. The inset HRTEM image in Figure 19a shows one typical SnSe nanoparticle,^350^ from which the obvious lattice and indexed (011) indicate the crystal nature of SnSe.

For SnSe nanoparticles, when the size is larger than certain values, they can have regular shapes, derived from the orthorhombic crystal nature of SnSe. At the same time, they can exhibit quantum confinement effect due to their small size. Experimental results based on ultraviolet, visible and near infrared wavelengths (UV–vis–NIR) indicated that a facile solution‐phase synthesis of well‐defined SnSe nanocrystals can still exhibit quantum confinement effects with measured direct optical bandgap of 1.71 eV,^351^ indicating these nanocrystals are attractive candidates for incorporation into thermoelectrics.

#### 7.2.2 Rod

Figure 19b shows typical SnSe nanorods fabricated through a typical solution route by using SnCl_2_·2H_2_O and SeO_2_ as precursors and OLA as solvent.^344^ The synthesized SnSe nanorods have an average length of ≈250 nm and width of ≈14 nm. The inset SAED pattern taken from a single rod in Figure 19b indicates the crystalline nature of SnSe. Figure 19c also shows a typical HRTEM of one nanorod, from which clear lattice can be seen, indicating that preferred growth direction of nanorods is along the [010] direction. The UV–vis–NIR results shown in the inset of Figure 19c indicate that a direct bandgap of 1.4 eV can be achieved in SnSe nanorods, which is favorable for applications in thermoelectrics.

#### 7.2.3 Belt

SnSe nanobelts have become the first measurable nanocrystals in thermoelectric SnSe. To fabricate SnSe nanobelts by advanced solution method, there are mainly two designs: one is using appropriate templates to guide the crystal growth of SnSe along specific directions, as shown in Figure 9h,^90^ and another is using appropriate doping and/or adjusting kinetic conditions such as solvent, synthesis temperature, and synthesis time to alter the preferential growth direction and morphology control, as shown in Figure 12c.^48^ Figure 19d shows a TEM image of a section of Cu‐doped SnSe nanobelt, synthesized through a solvothermal route with synthesis time of only 3 h. It is clear that a very thin nanobelt with a typical width of ≈600 nm can be seen. The inset SAED pattern in Figure 19d indicates that the nanobelt has typical orthorhombic crystal structure, viewed along the [100] direction. Such a nanobelt is a good candidate for thermoelectric microdevices and 2D flexible thermoelectric generators, as well as powders for sintering into bulk materials as components in thermoelectric modules, thus has a relatively wide application in thermoelectrics. Considering SnSe nanobelts have much lower superficial areas,^352^ a significant improvement of grain boundary density in the sintered bulks can be achieved, which can strengthen the phonon scattering and in turn reduce κ_l_.^48^

#### 7.2.4 Sheet

Compared with the other three types of nanocrystals, SnSe nanosheets are most promising because the synthesis of SnSe nanosheets with mature techniques is much easier than the syntheses of other SnSe nanocrystals,^353^ making SnSe nanosheets have full potentials as 2D/3D thermoelectric generators.^354^, ^355^ Generally, SnSe nanosheets can be described as multilayered SnSe.^356^, ^357^, ^358^, ^359^ Hydrothermal/Solvothermal‐based solution methods are effective routes to fabricate SnSe nanosheets.^26^, ^259^, ^264^ Different from the other routes, such as vapor deposition, the solution route can achieve SnSe nanosheets with a much higher productivity, which is essential for their applications as thermoelectric generators.

Figure 19e shows a TEM image of a SnSe nanosheet, synthesized through a solvothermal route, in which the synthesized nanosheet is very thin as significant bend contour can be seen. The inset SAED pattern in Figure 19e indicates that the nanosheet has a typical orthorhombic crystal structure, viewed along the [100] direction. Figure 19f also shows corresponding high‐resolution Cs‐corrected STEM‐HAADF image of a nanosheet,^48^, ^71^ viewed along the *a*‐axis. Considering that SnSe nanosheets have a thickness of only few SnSe layers, there is significant nanosize effect in these nanosheets. Experimental results based on UV–vis–NIR indicate that SnSe nanosheets have a direct bandgap of ≈1.1 eV, indicating that SnSe nanosheets possess considerable potentials as thermoelectric candidates, such as flexible thermoelectric generators and precursors for sintering bulk materials.

Except directly fabricating SnSe nanosheets by the solution methods, other convenient approach to achieve SnSe nanosheets is chemical exfoliation technique taken in a solution environment.^354^, ^355^, ^360^, ^361^, ^362^ In a Teflon‐lined autoclave, the bulk SnSe is mixed with a solution containing EG and lithium hydroxide (LiOH). During heating the autoclave, due to the weak Van der Waals forces between SnSe layers, lithium ions (Li^+^) can intercalate into these layers, separate the bulk into nanosheets with only a few layers thick.^361^ Based on this strategy, SnSe nanosheets with a considerable productivity can be achieved, demonstrating the possibility for fabricating 2D flexible thermoelectric generators.

### 7.3 Application

#### 7.3.1 Film

**Figure 20:**
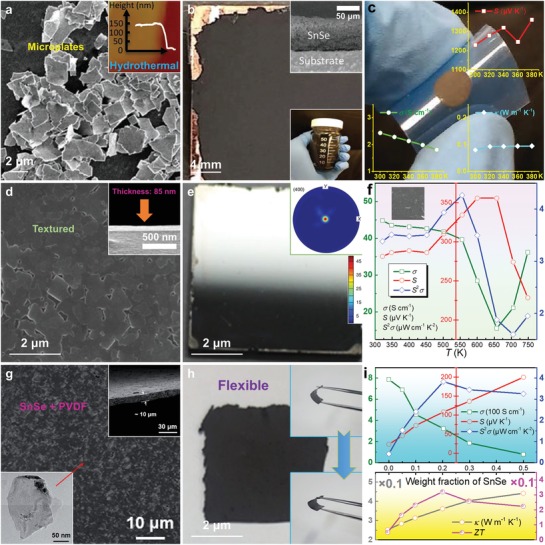
Characterizations and performance for SnSe‐based 2D thermoelectric generators. a) SEM image of hydrothermally synthesized SnSe nanosheets with AFM image inset showing their thickness of ≈150 nm. b) Optical image of scaling up SnSe thin film fabricated by these nanosheets with the packed bed structure of the SnSe film obtained from the scalable solution process (inset as bottom right) and its cross‐section image by SEM (inset as top right). c) *T*‐dependent thermoelectric performance of SnSe thin film, including σ, *S*, and κ. a–c) Reproduced with permission.^189^ Copyright 2019, Wiley. d) SEM image of SnSe thin film fabricated with purified precursor solution showing full coverage of SnSe on the substrate with cross‐sectional SEM image inset showing a uniform thickness of 85 nm. e) Optical image of SnSe thin film with its pole figure of the (400) plane inset showing the pole density in multiples of random distribution. f) *T*‐dependent thermoelectric performance of SnSe thin film, including σ, *S*, and *S* ^2^σ. d–f) Reproduced with permission.^250^ Copyright 2019, Springer Nature. g) SEM images of the CSA‐PANI coated SnSe_0.8_S_0.2_/PVDF composite film with cross‐sectional SEM image inset showing a uniform thickness of ≈10 µm and TEM image of SnSe_0.8_S_0.2_ nanosheets inset as inorganic fillers. h) Optical image of fabricated flexible thermoelectric generator. g,h) Reproduced with permission.^365^ Copyright 2018, Royal Society of Chemistry. i) SnSe amount‐dependent thermoelectric performance of organic‐inorganic composite film (SnSe/PEDOT:PSS), including σ, *S*, *S* ^2^σ, κ and ZT. Reproduced with permission.^360^ Copyright 2016, American Chemical Society.

The design of SnSe nanocrystals is for the fabrication of 2D flexible thermoelectric generators. Historically, there are mainly two ways to achieve this goal, one is fabricate SnSe‐based thin films by drop‐casting techniques and assemble the thin films with flexible substrates, and another is the organic/inorganic composite films from which the SnSe nanocrystals act as fillers. **Figure** **20**a shows an SEM image of hydrothermally synthesized SnSe nanosheets with AFM image inset showing their thickness of ≈150 nm.^189^ These nanosheets are good raw materials to fabricate SnSe thin films. Figure 20b shows an optical image of scaling up SnSe thin film fabricated by these nanosheets with the packed bed structure of the SnSe film obtained from the scalable solution process (inset as bottom right) and its cross‐section SEM image (inset as top right),^189^ from which a thin film of SnSe nanosheets was uniformly deposited using drop casting onto a copper tape substrate over an area of 1 cm^2^. The cross‐section SEM image of the film details the high density of this packed bed structure, which is critical for thermoelectric transportation. Figure 20c shows temperature‐dependent thermoelectric performance of fabricated SnSe thin film,^189^ including σ, *S*, and κ, respectively. Interestingly, giant *S* of >1200 µV K^−1^ and ultralow κ of <0.1 W m^−1^ K^−1^ were achieved for the entire temperature range from 300 to 380 K. The giant *S* with a relatively low σ of <2.5 S cm^−1^ come from a possible carrier filtering effect from the high density of interfaces.^189^ For the ultralow κ, different from the SnSe bulk materials (the κ gradually decrease with increasing the temperature), κ of fabricated SnSe thin film shows slight increase and indicates that the interface boundary thermal conductance dominates the phonon transport in the nanostructures. Such an ultralow κ are mainly caused by the thermally resistive interfaces between nanosheets.^363^ In the thin films, individual SnSe nanosheets are piled together and form multiple interfaces that dominate the thermal resistance.^364^ To realize a certain flexibility, the fabricated SnSe thin film was coated on a polyethylene terephthalate substrate, which exhibit significant flexibility under mechanical bending, as shown in Figure 20c.

To further strengthen the anisotropy and enhance the thermoelectric performance of SnSe thin films, highly textured and hole‐doped SnSe thin films were designed. Figure 20d shows an SEM image of SnSe thin film fabricated with purified precursor using solution route, in which full coverage of SnSe on the substrate can be seen. The inset cross‐sectional SEM image in Figure 20d shows a uniform thickness of ≈85 nm.^250^ Figure 20e shows an optical image of SnSe thin film, showing typical metallic luster. The inset pole figure of the (400) plane in Figure 20e shows the pole density in multiples of random distribution,^250^ indicating a high anisotropy of the thin films. Figure 20f shows temperature‐dependent thermoelectric performance of the SnSe thin film, including σ, *S*, and *S* ^2^σ,^250^ respectively. As can be clearly seen, a high *S* ^2^σ of ≈4.3 µW cm^−1^ K^−2^ was achieved at 550 K, which is competitive to the corresponding bulk materials,^3^ derived from a high σ of ≈39.7 S cm^−1^ and an appropriate *S* of ≈334.1 µV K^−1^ at this temperature, and the hole doping plays a significant role in balancing σ and *S* contributing to high *S* ^2^σ. Besides, the solution process for achieving high performance SnSe thin films is a cost‐effective process. The current SnSe ink solution can be further applied to more practical solution process to fabricate applicable SnSe thick films and flexible thermoelectric generators.

#### 7.3.2 Organic/Inorganic Composite

For SnSe and polymer‐based organic/inorganic composite films, they are particular fascinating because of their intrinsic flexibility, affordability, and low toxicity.^366^ For the organic substrates of composite films, polydimethylsiloxane (PDMS),^357^ polypyrrole,^367^, ^368^ polyvinylidene fluoride (PVDF),^365^ polythiophene,^360^, ^369^ polyaniline (PANI),^370^, ^371^ and poly (3,4‐ethylenedioxythiophene):poly (styrenesulfonate) (PEDOT:PSS)^372^ are promising candidates due to their relatively high σ and intrinsically low κ.^373^, ^374^, ^375^, ^376^, ^377^, ^378^ Figure 20g show an SEM image of the camphorsulfonic acid (CSA)‐doped PANI‐coated SnSe_0.8_S_0.2_/PVDF composite film.^365^ The inset cross‐section SEM image in Figure 20g shows a uniform thickness of ≈10 µm. The inorganic fillers of the composite film are SnSe_0.8_S_0.2_ nanosheets achieved by chemical exfoliation technique taken in a solvothermal environment with EG as solvent, and one as‐synthesized nanosheet is shown in the inset TEM image in Figure 20g.^365^ Figure 20h shows an optical image of fabricated composite film,^365^ the inset images indicate a good flexibility in as‐fabricated film, which is a typical flexible thermoelectric generator. Besides, by introducing a small amount of carbon nanotubes (CNTs) into the composite, the maximum *S* ^2^σ of the CSA‐PANI‐coated SnSe_0.8_S_0.2_‐PVDF/CNT composite film is 2.97 µW cm^−1^ K^−2^ at 400 K, which is significantly higher than that of the CSA‐PANI‐coated SnSe_0.8_S_0.2_‐PVDF composite film without CNTs (1.17 µW cm^−1^ K^−2^ at 400 K).^365^

Among the organic substrates applied to composite film, PEDOT:PSS is one of the most promising polythiophene‐based polymer,^372^ which possesses remarkable high σ.^374^, ^375^, ^376^, ^377^ To illustrate the SnSe‐PEDOT:PSS‐based thermoelectric composite films, Figure 20i shows the SnSe content‐dependent thermoelectric performance, including σ, *S*, *S* ^2^σ, (h) κ, and ZT,^360^ respectively. The SnSe nanosheets acted as fillers were prepared by chemical exfoliation technique taken in a solvothermal environment with EG as solvent. With increasing the SnSe content in the composite films, σ decreases and *S* increases, indicating a balance between SnSe fillers with intrinsic low σ and high *S* and PEDOT:PSS substrates with intrinsic high σ and low *S*, contributing a peak *S* ^2^σ of ≈3.8 µW cm^−1^ K^−2^ in the SnSe‐PEDOT: PSS composites when the SnSe content was 20 wt%. Meanwhile, a low κ of ≈0.36 W m^−1^ K^−1^ can be achieved when the SnSe content was 20 wt%, derived from the intrinsic low κ of SnSe nanosheets, and contributed to a promising peak ZT of ≈0.32, which is very competitive compared with other flexible thermoelectric generators as well as corresponding bulk materials. These results demonstrate that SnSe with intrinsic high *S* and low κ are good candidates for their applications as flexible thermoelectric generators.

#### 7.3.3 Overview

Currently, the research focusing on SnSe‐based 2D and flexible thermoelectric generators is still at preliminary stage, but some promising results have been achieved, and the strategy of advanced solution route is playing a significant role in determining their performance. **Table** **4** provides a summary on the thermoelectric performance of 2D SnSe made through solution‐based routes, from which huge potentials can be seen in further improving the properties of 2D SnSe thermoelectric generators, which represent the future development directions of SnSe‐based thermoelectrics.

**Table 4:** A comprehensive summary on the thermoelectric performance of 2D SnSe via solution‐based routes. Here the carbon nanotubes, hot‐wall epitaxy, vacuum deposition, reactive evaporation, pulsed laser deposition, magnetron sputtering, chemical exfoliation, drop‐casting, and flash evaporation are abbreviated as CNTs, HWE, VD, RE, PLD, MS, CE, DC, and FE, respectively. The units for *T*, σ, *S*, *S^2^σ*, κ, and *n*/*p* are K, S cm^−1^, µV K^−1^, µW cm^−1^ K^−2^, W m^−1^ K^−1^, and 10^19^ cm^−3^, respectively. The marked * in *n*/*p* column means the *n*/*p* values were tested at RT

Description	Type	Synthesis	ZT	*T*	σ	*S*	*S^2^σ*	κ	*n*/*p*	Ref.
20 wt% SnSe nanosheets + PEDOT:PSS	*p*	SSR + MA + CE + HT + DC	0.32	300	≈324.2	≈109.1	≈3.9	≈0.35	120	360
20 wt% SnSe nanosheets + PEDOT:PSS	*p*	SSR + MA + CE + HT + DC	–	300	≈320.0	≈110.0	≈3.9	–	–	362
SnSe_0.97_Te_0.03_ nanosheets + PEDOT:PSS	*p*	SSR + MA + CE + HT + DC	–	–	≈18.5	≈90.0	≈0.15	–	1.73	379
SnSe_0.8_S_0.2_ nanosheets + PANI + CNTs	*p*	SSR + MA + CE + HT + DC	–	400	≈30.0	≈316.0	≈3.0	–	–	365
PEDOT‐coated SnSe_0.97_Te_0.03_ nanosheet + 20 wt% PEDOT:PSS composites	*p*	SSR + CE + HT + DC	0.18	300	≈320.0	≈80.0	≈2.0	≈0.36	–	380
PEDOT‐coated SnSe_1−_ *_x_*Te*_x_* nanosheet + PEDOT:PSS multilayers	*p*	SSR + CE + HT + DC	–	300	≈150.0	≈124.0	2.22	–	–	381
Porous SnSe_0.8_S_0.2_ nanosheets	*p*	AS	0.12	310	≈4.76	≈569.4	≈1.5	≈0.40	≈0.52	361
Hole‐doped SnSe thin film	*p*	AS	–	550	≈40.0	≈350.0	≈4.3	–	≈0.35	250
SnSe thin film	*p*	AS	–	573	0.9	186	–	–	–	382
SnSe thin film	*n*	AS	–	300	≈67.5	≈−90.0	≈0.55	–	–	265
SnSe thin film	*p*	HT	–	300	≈2.4	≈1200.0	≈3.5	≈0.09	–	189
SnSe nanosheets + PDMS	*p*	CVD	–	120	≈0.06	866.0	≈0.04	–	–	357
1% Ag‐doped SnSe thin film	*p*	CVD	–	300	≈5.14	≈370.9	≈0.7	–	≈0.04	383
SnSe thin film	*p*	RE	1.2	42	≈0.11	7863.0	≈7.2	≈0.025	≈0.03	384
SnSe thin film	*p*	PLD	0.45	800	28.3	264	≈2.0	0.35	–	385
SnSe thin film	*p*	PLD	–	343	≈21.3	≈263.4	≈1.5	≈0.19	–	386
SnSe thin film	*p*	PLD	–	673	≈30.0	≈310	≈4.0	–	≈0.7 *	387
Sn_0.85_Ca_0.15_Se thin film	*p*	PLD	–	300	≈13.6	≈367.2	≈1.9	–	≈0.02	388
SnSe thin film	*p*	TE	≈0.055	500	≈1.36	≈287.3	≈0.1	≈0.08	–	389
Sn_1−_ *_x_*Se thin films	*p*	TE	–	425	≈2.84	≈111.1	≈0.04	≈0.78	–	390
SnSe thin film	*n*	TE	–	473	47	≈−159.8	1.2	–	≈0.03 *	391
Sn_0.97_Se thin films	*p*	TE	–	300	≈0.61	–	–	–	≈0.14	392
SnSe thin film	*p*	TE	–	550	≈0.05	–	–	–	–	393
(SnSe)_0.66_(SnSe_2_)_0.34_ thin film	*n*	TE	–	523	≈23.8	−255	≈1.6	–	–	394
2.6 at% Mo‐SnSe multilayer thin film	*p*	MS	–	576	≈8.5	≈220.0	≈0.4	–	≈0.13 *	395
SnSe thin film	*p*	MS	–	575	≈11.0	≈350.0	1.4	–	0.00043 *	396
SnSe thin film	*p*	HWE	–	400	≈5.0	–	–	–	≈1.1	397
SnSe thin film	*p*	HWE	–	400	≈2.5	–	–	–	≈0.01	398
SnSe thin film	*p*	FE	–	512	≈0.1	–	–	–	–	399
SnSe thin film	*p*	FE	–	400	–	–	–	–	≈0.06	400
SnSe thin film	*p*	VD	–	300	–	–	–	–	≈50	401

## 8 Conclusion, Challenge, and Outlook

Compared with traditional melting and mechanical alloying routes, advanced aqueous synthesis, especially hydrothermal/solvothermal‐based solution methods are attracting increasing attentions in recent years, derived from their unique characteristics for improving their thermoelectric performance. Through adjusting appropriate kinetic conditions during synthesis, such as solvents, precursors, amounts, pH adjusters, surfactants, catalysts, temperatures, time, and vapor pressure, various specific strategies can be achieved, including vacancy engineering, morphology controlling for both crystalline size and type, raising the doping limit, realizing impossible doping for both p‐type and n‐type, and inducing local lattice defects to significantly strengthen the phonon scattering, which are all unique features for hydrothermal/solvothermal routes. For SnSe, hydrothermal/solvothermal‐based solution methods are especially suitable for SnSe‐based thermoelectric materials because the thermodynamics of SnSe are well aligned with the kinetic conditions of synthesis, thus there are considerable potentials for materials design. Meanwhile, because SnSe are typical orthorhombic layered structure, hydrothermal/solvothermal synthesis can conveniently realize morphology control of synthesized SnSe crystals to achieve either high thermoelectric performance due to anisotropy strengthening or robust mechanical properties due to grain refinements. A high peak ZT of ≈2.2 can be achieved in polycrystalline SnSe fabricated by a hydrothermal route by 1% Zn and 1% Pb codoping,^16^ and a high average ZT of ≈0.9 can be achieved in polycrystalline SnSe fabricated by a solvothermal route with EG as solvent and Cd‐doping,^22^ both indicating that solvothermal‐based solution methods can achieve significantly competitive thermoelectric performance in polycrystalline SnSe. Besides, benefitted from the advantages of convenient morphology control, hydrothermal/solvothermal synthesis are specifically suitable for fabricating SnSe nanocrystals with various sizes and types, which are good candidates for fabricating 2D and flexible thermoelectric generators. In summary, when hydrothermal/solvothermal meet thermoelectric SnSe, there will be infinite potentials for promoting the development of SnSe‐based thermoelectrics, and hydrothermal/solvothermal methods applied to thermoelectric SnSe can also provide new perspectives as reference for other thermoelectric systems, such as PbTe,^402^ Cu_2_Se/Cu_2_S,^245^, ^403^, ^404^, ^405^, ^406^, ^407^ Bi_2_Te_3_,^242^, ^243^, ^247^, ^408^, ^409^, ^410^, ^411^, ^412^ and SnTe,^413^, ^414^, ^415^, ^416^, ^417^, ^418^ which are also friendly to hydrothermal/solvothermal‐based aqueous solution methods.

It should be noted that there are still some controversies on SnSe‐based thermoelectric materials fabricated by hydrothermal/solvothermal routes. For polycrystalline SnSe bulks synthesized by hydrothermal routes, the main controversies are focusing on their special thermoelectric performance at high temperatures, especially over the phase transition. Reported studies indicated that *S* and σ can simultaneously increase when *T* > 800 K, leading to unpredictable high *S* ^2^σ and in turn high ZT of > 2, which is contradict with fundamental physics. This controversy indicates that there might be very complex physics/chemistry involved in the measured data, which cannot be explained in current knowledge and need further exploring. At the same time, a continuous decrease of κ over the phase transition temperature of 800 K can be seen, which is different from traditional routes as well as solvothermal routes (a sudden rise of κ over ≈800 K). The potential reasons may be that the sintering temperatures of <700 K ware much lower than the temperature to achieve peak ZTs (≈900 K), which may cause considerable microcracks in polycrystalline SnSe by phase transitions, leading to underestimated κ which cannot show the intrinsic thermal transport performance of SnSe at these temperatures. Thus, a critical standard of measurement should be established for achieving reliable measured data.^419^ Furthermore, considering the significant thermal release near the phase transition of ≈800 K, the thermal diffusivity *D* measured at this temperature may be seriously underestimated. Meanwhile, for SnSe which is a strong‐anharmonic material, there is *C*_p_ > *C*_v_ at elevated temperatures but the discrepancy between *C*_p_ and *C*_v_ is offset to the first order by a decrease in the packing density due to thermal expansion. However, this is not the case near the phase transition temperature. Hence, we anticipate that *C*_p_ = *C*_v_ at all temperatures, except at the phase transition temperature, leading the reported ZT value near the phase transition be inaccurate due to inaccurate *C*_v_ and the total κ derived from the measured *C*_p_. Considering that the phase transition can also weaken the mechanical properties,^9^ it is recommended to avoid reporting ZTs too close to ≈800 K.^420^ Previous studies also indicated that special increases of μ can be found close to phase transition,^12^, ^22^, ^24^, ^48^, ^71^ as shown in Figure 8e, indicating that additional scattering mechanisms exist in these systems at these temperatures. More studies are needed to explain such special mobility behaviors near the phase transition temperatures. Last but not least, for hydrothermal/solvothermal synthesized SnSe crystals, some dopants such as alkaline metals Na and K with high chemical activities were difficult to be doped into SnSe system due to the high formation energies of Na^+^ and K^+^.^267^ We have summarized the doping potentials in SnSe under fixed kinetic conditions, as shown in **Table** **5**, including dopants, sources, products, imperfections, dimensions, doping limits, types, and secondary phases. The solvent was EG, the Sn source was SnCl_2_·2H_2_O, the Se source was Na_2_SeO_3_, the pH adjuster was NaOH, the synthesis temperature was at 503 K, the synthesis time was 36 h, and the amount of dopant sources were all 10%. As can be seen, many doping cannot be achieved if the kinetic conditions were not well aligned with the doping thermodynamics, and the producing of secondary phases is also a significant issue when they were hard to be removed from the synthesized SnSe crystals in some cases. More studies are needed to further explore the potentials of hydrothermal/solvothermal synthesis on fabricating thermoelectric SnSe.

**Table 5:** A comprehensive summary for solvothermal doping potentials in SnSe under fixed kinetic conditions. Here the solvent is EG, the Sn source is SnCl_2_·2H_2_O, the Se source is Na_2_SeO_3_, the pH adjuster is NaOH, the synthesis temperature is 503 K, synthesis time is 36 h, and the amount of dopant source is 10%

Dopant	Dopant source	Product	Imperfection	Average size [µm]	Doping limit	Type	Secondary phase
Mn	MnCl_2_	Microplate	Breach	40 × 55 × 5	0	p	MnO
Co	CoCl_2_·6H_2_O	Microplate	Breach, agglomeration	95 × 110 × 10	0	p	CoO
Cu	CuO	Microbelt	Crack, stepped surface, bent	400 × 10 × 5	10.5%	p	Cu_2−_ *_x_*Se
Cu	CuCl_2_	Microbelt	Crack, stepped surface, bent	450 × 10 × 5	11.8%	p	Cu_2−_ *_x_*Se
Zn	ZnCl_2_	Microplate	Breach, contamination	70 × 85 × 9	0	p	ZnO
Ga	Ga_2_O_3_	Microplate	Breach	40 × 50 × 7	0	p	–
As	As_2_O_3_	Microplate	Breach, contamination	90 × 105 × 10	0	p	As_2_O_3_
Br	NaBr	Microplate	Breach, contamination	45 × 60 × 7	1.6%	n	Sn
Br	SnBr_2_	Microplate	Breach, contamination	50 × 65 × 7	1.1%	n	Sn
Sr	SrCl_2_·6H_2_O	Microplate	Breach	55 × 70 × 7	0	p	SrO
Ag	AgNO_3_	Microplate	Breach	50 × 70 × 8	0	p	Ag nanowire
Ag	Ag	Microplate	Breach	40 × 55 × 7	0	p	Ag nanowire
Ag	Ag_2_O	Microplate, microrod	Breach	35 × 45 × 6	0.9%	p	Ag nanowire
Cd	CdCl_2_	Microplate	Breach, crack, hollow, agglomeration	110 × 135 × 9	2.3%	p	CdSe
In	In_2_O_3_	Microplate	Breach, stepped surface, bent, agglomeration	25 × 25 × 3	0	p	In_6_Se_7_, In_4_Se_3_, InSe
In	InCl_3_·4H_2_O	Microplate	Breach, stepped surface, bent, agglomeration	25 × 25 × 3	0	p	In_6_Se_7_, In_4_Se_3_, InSe
Sb	SbCl_3_	Microplate	Breach, crack, stepped surface, bent, agglomeration	40 × 55 × 4	3.0%	n	Sb_2_Se_3_
Sb	Sb_2_O_3_	Microplate	Breach, crack, stepped surface, bent, agglomeration	70 × 85 × 6	3.2%	n	Sb_2_Se_3_
Te	Na_2_TeO_3_	Microplate	Breach, stepped surface	65 × 80 × 9	12.3%	p	SnTe
I	NaI·2H_2_O	Microplate	Breach, contamination	50 × 60 × 8	0.3%	p	Sn
Cs	CsNO_3_	Microplate	Breach	45 × 55 × 7	0	p	–
Cs	CsCl	Microplate	Breach	45 × 55 × 7	0	p	–
Ta	TaCl_5_	Microplate	Breach, contamination	40 × 55 × 7	0	p	Ta_2_O_5_
Bi	Bi(NO)_3_·5H_2_O	Microplate	Breach	60 × 75 × 8	0	p	Bi_2_Se_3_
Bi	BiCl_3_	Microplate	Breach, crack	75 × 90 × 9	0.7%	n	Bi_2_Se_3_
Bi	Bi_2_O_3_	Microplate	Breach, crack	80 × 100 × 10	0.9%	n	Bi_2_Se_3_

**Figure 21:**
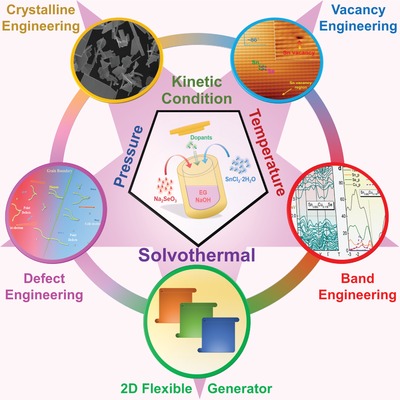
Illustration of outlooks for aqueously synthesized SnSe thermoelectrics.

For the outlook of hydrothermal/solvothermal‐based thermoelectric SnSe, there are mainly five aspects for attentions, as shown in **Figure** **21**:

1. *Optimizing Sn/Se vacancies*: Vacancy engineering is one of the most unique advantages in solution‐based synthesized SnSe because it can simultaneously achieve high thermoelectric performance m tuning *n*/*p* by inducing appropriate vacancies, and robust mechanical properties by vacancy‐induced dispersion strengthening for the grains.^314^, ^315^, ^316^ However, the vacancy concentration in synthesized SnSe still not reach the optimized value. For p‐type SnSe, doping with other elements with stable +2 valence state but different atomic size of Sn is a good choice to reduce the vacancy formation energy, contributing to a more optimized *p*, and Cd‐doped SnSe is a typical case;^22^ for n‐type SnSe, because of the much higher formation energy of V_Se_ than that of V_Sn_, as shown in Figure 7b, achieving Se vacancies in pure SnSe is historically difficult, thus inducing foreign atoms to substitute Se sites to further reduce the formation energy of V_Se_ is a good choice, and Sb‐doped SnSe is a typical case. Considering that the doped Sb can also cause intensive crystal imperfections which seriously impede the electrical transport performance, more suitable candidates should be explored to achieve higher thermoelectric performance of SnSe.
2. *Strengthening anisotropy or grain refinement*: SnSe has a typical orthorhombic layered structure, making it a typical anisotropy semiconductor. To further strengthen high thermoelectric performance in polycrystalline SnSe along specific direction (mainly the ⊥ direction), highly texturing is needed in sintered polycrystalline SnSe, which can be realized by hydrothermal/solvothermal‐based solution methods, derived from the convenient morphology control. However, how to control the SnSe crystal size still need to be addressed, and further exploring appropriate kinetic conditions should be considered. Oppositely, to achieve robust mechanical properties and much balanced thermoelectric performance along different directions for their practical applications in thermoelectric devices, the grain refinement may be needed, and hydrothermal/solvothermal‐based solution routes can also achieve this goal by adjusting corresponding synthesis parameters, and microwave‐assistance is a good choice to achieve nanocrystals with a high productivity within a very short time.
3. *Appropriate doping*: SPB modellings indicate that vacancy engineering can achieve a peak ZT of ≈1.85 at 823 K when *p* is set as ≈3 × 10^19^ cm^−3^ with a reduced κ_l_ of ≈0.2 W cm^−1^ K^−1^.^3^, ^22^ However, to breakthrough the limit of peak ZT, a band manipulation is needed to alter the band structure and/or bandgap of SnSe, and doping with foreign atoms is a good choice to achieve this goal. As shown in Table 5, in order to achieve different doping in SnSe system, complex alignment of kinetic conditions with doping thermodynamics are needed to either breakthrough the doping limits or realize new doping, thus exploring suitable synthesis parameters are recommended.
4. *Balancing the electrical and thermal transport performance*: Hydrothermal/solvothermal‐based solution methods have demonstrated that they can induce various types of nanosized local lattice imperfections to strengthen the phonon scattering at the induced strain fields, which is one of the unique characteristics in synthesized SnSe. However, sometimes such intensive local lattice defects may also scatter the carriers significantly, leading to a much reduced μ and in turn a low σ, such as the case of solvothermally Sb‐doped SnSe.^71^ In this situation, a balancing is important to couple the σ and κ values and in turn achieve high ZT by controlling the density of induced local lattice imperfections by appropriately adjusting the synthesis parameters.
5. *Flexible thermoelectric generators*: As discussed in Section 7.3, the research focusing on SnSe‐based 2D and flexible thermoelectric generators is still at preliminary stage, but hydrothermal/solvothermal‐based solution routes are playing significant roles in improving their performance because it can simultaneously realize morphology control for achieving nanocrystals and tuning *n*/*p* for achieving high performance. Considering that personal‐used flexible thermoelectric devices have much wider applications compared with traditional module‐based thermoelectric devices, the design of 2D and flexible thermoelectric generators represent the directions of future development of thermoelectrics. SnSe and hydrothermal/solvothermal synthesis, however, are two keys to unlock this mysterious treasure box, thus have full potentials and should be paid significant attentions in future studies.

## Conflict of Interest

The authors declare no conflict of interest.
